# Deciphering Asymmetric Induction in Photoredox Catalysis by Chiral Counteranions

**DOI:** 10.1021/acscatal.5c07578

**Published:** 2025-12-19

**Authors:** Lorenzo Baldinelli, Sofia Lerda, Riya Kayal, Frank Neese, Filippo De Angelis, Giovanni Bistoni

**Affiliations:** † Dipartimento di Chimica, Biologia e Biotecnologie, 9309 Università degli Studi di Perugia, Perugia 06123, Italy; ‡ Max-Planck-Institut für Kohlenforschung, Kaiser-Wilhelm Platz 1, Mülheim an der Ruhr 45470, Germany; § Dipartimento di Chimica, Biologia e Biotecnologie, Università degli Studi di Perugia and INSTM, Perugia 06123, Italy; ∥ Computational Laboratory for Hybrid/OrganicPhotovoltaics (CLHYO), Istituto CNR di Scienze e Tecnologie Chimiche “Giulio Natta” (CNR−SCITEC), Perugia 06123, Italy; ⊥ SKKU Institute of Energy Science and Technology (SIEST), Sungkyunkwan University, Suwon 440-746, Korea

**Keywords:** asymmetric catalysis, photoredox processes, chiral ion pairs, reaction mechanism, noncovalent interactions, London dispersion, computational catalysis, DFT

## Abstract

We investigate the origin of stereocontrol in asymmetric counteranion-directed photoredox catalysis (ACPC) using a representative [2 + 2] cycloaddition mediated by a chiral imidodiphosphorimidate (IDPi) counteranion (Science 2023, 379, 494−499). Combining extensive conformational sampling, high-level DFT calculations, and multiscale modeling, we elucidate the mechanism and stereochemical landscape of this transformation. Both enantio- and diastereoselectivity are established in the first C−C bond-forming step: diastereoselectivity arises from intrinsic aryl−aryl interactions within the radical cation−styrene pair, whereas enantioselectivity is imposed by the confined chiral environment of the IDPi counteranion. Although electronically silent during the initial photoinduced single-electron transfer, the counteranion anchors the radical cation and organizes its cycloaddition with styrene. Atomic decomposition of the London dispersion (ADLD) and molecular dispersion potential (MDP) analyses reveal that attractive van der Waals forces, shaped by the steric and electronic architecture of the counteranion, promote reactive prealignment of the substrates and selectively stabilize the transition state, leading to the major product. Extension to substituted styrenes shows that ring substitution reconfigures the noncovalent contact map within the catalyst pocket, reshaping the energetic balance between competing pathways, in line with experiment. These findings provide a unified framework for stereocontrol in chiral ion-pair radical catalysis and offer general strategies for designing asymmetric photoredox transformations.

## Graphical abstract

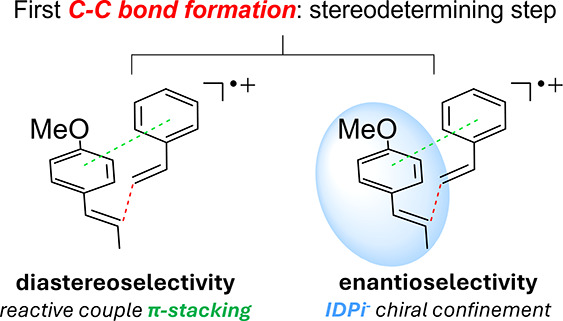

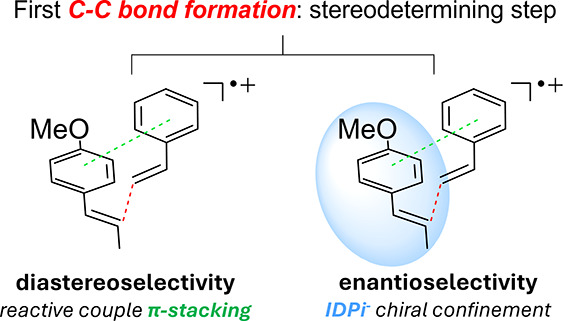

## 1 Introduction

Photoredox catalysis has emerged as a powerful platform for enabling novel reactivity under mild, visible-light conditions, harnessing photon energy to generate highly reactive radicals, species that are often inaccessible or unstable in traditional (two-electron) reaction mechanisms.^1–3^ The use of single-electron transfer (SET) processes to unlock radical reactivity has profoundly expanded the synthetic repertoire, especially with the introduction of transition-metal complexes as photoredox catalysts.^4–6^ These systems, characterized by exceptional photophysical properties and tunability, have catalyzed an array of transformations ranging from C−C couplings to selective functionalizations.^4,7,8^

However, the push toward more sustainable and operationally simple catalysis has fostered increasing interest in metal-free photoredox platforms.^9–11^ Organic photocatalysts have demonstrated broad utility, combining environmental compatibility with redox activity and visible-light responsiveness.^12,13^ However, achieving stereocontrol in such open-shell systems has remained a central challenge.^14,15^ The transient nature and high diffusivity of radical intermediates often preclude direct stereocontrol, and as a result, most successful enantioselective photoredox methods rely on dual catalytic systems, where a photoredox cycle is merged with a second, stereocontrolling catalyst.^16–21^ While effective, these approaches introduce mechanistic complexity and often require careful balancing of two distinct catalytic manifolds. Thus, the development of unified, metal-free platforms capable of promoting both radical generation and asymmetric induction remains a critical objective in the field.

In this context, a major conceptual advance was recently introduced by List and co-workers, who demonstrated that stereocontrol can instead be achieved via asymmetric counteranion-directed photoredox catalysis (ACPC), illustrated in Scheme 1.^22^

**Scheme 1:**
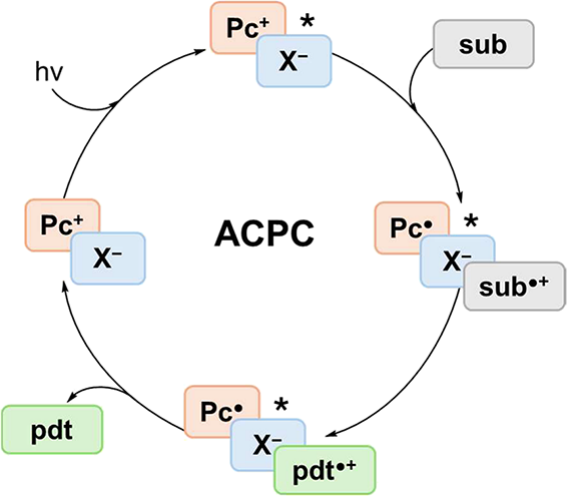
Schematic Overview of Asymmetric Counterion-Directed Photoredox Catalysis (ACPC)^a^

a Upon absorption of photon energy (*h*ν), a photoexcitable cationic photocatalyst (**Pc** ^**+**^) oxidizes the substrate (**sub**) via single-electron transfer (SET), generating a radical cation (**sub** ^**·+**^). Association with a chiral anion (**X** ^**−**^) forms a confined ion pair, which imposes stereocontrol needed for an enantioenriched product (**pdt**).

In this strategy, a photooxidant cation (e.g., pyrylium) is paired directly with a chiral anion, such as imidodiphosphorimidate (IDPi),^23^ forming a confined, catalytically active ion pair. Under visible-light irradiation (blue LEDs) and without metals or second cocatalysts, the cationic photocatalyst oxidizes trans-anethole (or variants where *p*-OMe is replaced by *p*−OH or *p*-OBn) to its radical cation, which then undergoes a [2 + 2] cycloaddition with a styrene partner inside the pocket of the IDPi. Styrenes substituted at various positions of the aromatic ring were also examined. Across this substrate set, the ion-pair catalyst consistently delivers cyclobutanes, as illustrated by the targeted transformation shown in Figure 1a, with high enantio- and diastereocontrol under mild reaction conditions (blue light, ambient temperature, standard organic solvent). List speculated a stepwise pathway in which enantioselectivity is determined during the first C−C bond-forming event and diastereoselectivity arises in a subsequent ring closure. However, either computational or experimental evidence supporting this model remains lacking. Additionally, other important aspects remain open: how the individual components of the ion pair participate in the photoinduced SET event, the modality with which the chiral counteranion shapes stereocontrol, and the extent to which the composition of the coupling partners influences selectivity.

Radical-cation [2 + 2] cycloadditions with electron-rich aryl alkenes have been documented across photoredox, oxidative, and electrochemical manifolds, underscoring the versatility and synthetic utility of this class of transformations.^24–26^ Focusing on systems closely related to those examined here, two computational contributions are particularly instructive for the mechanism and diastereocontrol: Wiest introduced the mechanistic analysis of radical-cation [2 + 2] pathways,^27^ while Paton later provided a detailed dissection of the trans-anethole/trans-β-methylstyrene pair,^28^ closely related to the present system. These contributions traced the stepwise mechanism to cyclobutane and identified the inherent diastereoselectivity of the uncatalyzed substrate pair. What has remained unresolved is how such intrinsic diastereocontrol can be translated into high enantioselectivity. Embedding the radical manifold within the chiral environment of an IDPi counteranion directly addresses this gap, reconciling the substrate-driven bias with the counteranion-enforced selectivity.

From a computational perspective, capturing the interplay between the catalyst architecture and the intrinsic composition of the substrate is especially challenging. Enantioselective outcomes often hinge on subkcal·mol^−1^ energy differences between competing transition states, and in ACPC, the difficulty is magnified by the supramolecular nature of the catalytic ensemble, which involves up to four interacting species (the radical cation donor, the olefin partner, the IDPi, and the photocatalyst). The IDPi alone spans a broad conformational landscape, and its dynamic interplay with radical intermediates generates a challenging energy surface where noncovalent interactions are decisive. Addressing this problem requires state-of-the-art theory that is both scalable and diagnostic: a protocol that treats all components of the supramolecular assembly explicitly, remains computationally feasible without sacrificing accuracy, and resolves how noncovalent forces convert supramolecular complexity into stereocontrol.

Motivated by these open questions and challenges, we investigated the mechanism and stereochemical landscape of this transformation. Our approach combines exhaustive conformational sampling, extensive DFT optimization, and a multilayer embedding strategy that captures the effects of the full catalytic system (including the bulky chiral counteranion) while maintaining high accuracy at the reactive couple formed by the trans-anethole radical cation (**sub** ^**·+**^) and styrene. We show that the confined geometry and dispersion-rich topology of IDPi,^29^ in concert with the structural and electronic features of the reacting partners, cooperatively dictate the experimentally observed stereochemical outcome, governing enantio- and diastereoselectivity through distinct yet complementary interactions. We hope that these insights will not only advance the understanding of stereocontrol in ACPC but also emphasize the opportunity to leverage the synergistic design of catalyst−substrate to enhance selectivity across a broader range of transformations.

## 2 Methods

All quantum chemical calculations were performed with the ORCA suite (development versions 5.0 and 6.1).^30,31^ The conformational space of key intermediates was explored via conformational sampling simulations using CREST at the GFN2-xTB level.^32,33^ Resulting ensembles were iteratively filtered by relative energy and RMSD: at each step, among conformers separated by ≥1.5Å RMSD from the current reference structure, the lowest-energy conformer was retained for DFT reoptimization and designated as the reference for the subsequent iteration, yielding a nonredundant, representative, and tractable subset. The so-selected structures were fully reoptimized at the DFT level (PBE-D3BJ/def2-SVP) with CPCM solvation in CH_2_Cl_2_.^34–38^ Frequency calculations were used to confirm the nature of stationary points and obtain thermochemical corrections at 298.15 K. Final single-point energies were computed with the ωB97X-D3BJ functional^39^ and the def2-QZVP basis set,^40^ using the same solvent model. Both geometry optimizations and energy refinements were carried out using the VeryTightSCF convergence criteria. The combination of PBE-D3BJ/def2-SVP geometries with high-level single-point energy corrections proved effective for accurately describing a range of closely related reactions.^29,41–43^ Open-shell stationary points were treated as triplets when the photocatalyst was included in the system (see discussion in Section 1 of the Results) and as doublets when not included. In addition, the composite protocol was validated through TDDFT calculations (ωB97X-D3BJ/def2-TZVP, NROOTS = 50, with the Tamm−Dancoff Approximation) against the experimental UV−Vis spectrum of **[Pc^+^−X^−^]** (Figure S1); the same setup was subsequently employed for all TDDFT calculations reported in the manuscript, including the electronic difference density between the ground and first singlet excited states of **[Pc** ^**+**^ **−X** ^**−**^ **]** (Figure S2).

To investigate enantioselectivity across the multiple stereochemical pathways, each involving large intermediates and transition states, we employed a multilayer ONIOM (QM:xTB) strategy.^44^ The reactive couple (**sub** ^**·+**^ and styrene) was treated at the PBE-D3BJ/def2-SVP level, the counteranion **X** ^**−**^ was described with GFN2-xTB, and solvation effects were included with the ALPB model.^45^ Transition states were located using NEB-TS and relaxed scans, and confirmed by vibrational analysis.^46^ Electronic energy refinements were performed on the ONIOM-optimized geometries, with the QM region evaluated at the ωB97X-D3BJ/def2-QZVP level, while the remaining region was kept at the xTB level. London dispersion effects on the key transition states were quantified using Atomic Decomposition of London Dispersion (ADLD) analysis.^47,48^ Additionally, molecular dispersion potential (MDP) analysis was carried out using an in-house implementation, as described in ref 49 to rationalize the reactive couple preorganization imposed by **X^−^** within its catalytic pocket.

## 3 Results

### 3.1 Thermodynamic Landscape and Electron Transfer Initiation

We began our study by analyzing the catalytic cycle depicted in Scheme 1.^22^ The robust multistep workflow described in the previous section, encompassing both conformational sampling simulations and full DFT optimizations, was initially used to enable a systematic identification of the lowest-energy intermediates along the reaction pathway (Figure 1).

**Figure 1:**
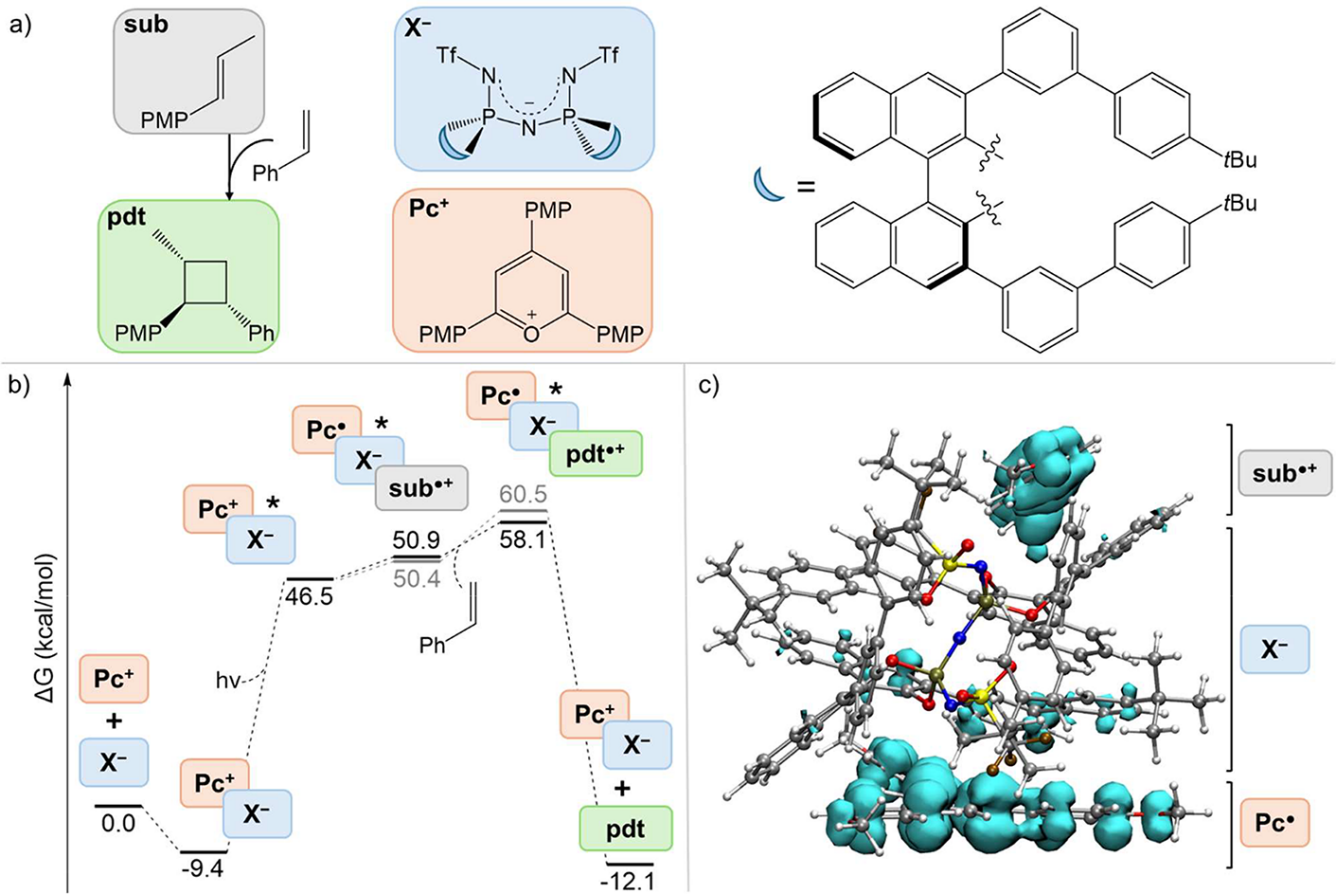
(a) Relevant species of the [2 + 2] photocycloaddition, along with the corresponding label. Abbreviations: PMP, para-methoxyphenyl; Tf, triflyl; Ph, phenyl; tBu, *tert*-butyl. (b) Computed free energy profiles for the two mechanistic models considered: with (gray) and without (black) the neutral radical **Pc^·^** after SET. (c) Spin density (cyan) for the triplet state of the ion pair together with **sub**, showing charge-separated radical character consistent with a photoinduced SET process from **sub** to **Pc** ^**+**^.

The key species considered are listed in Figure 1a. The reaction initiates with the photoexcitation of the chiral ion pair [**Pc** ^**+**^−**X^−^**], which enables a single-electron transfer from the trans-anethole substrate (**sub**) to the photoexcited cation (**Pc** ^**+**^). This step generates the substrate radical cation (**sub^·^** **^+^**) and neutral radical species **Pc^·^**. The **sub^·^** **^+^** species then undergoes a [2 + 2] cycloaddition with the styrene reactant, forming the corresponding radical cation product (**pdt^·^** **^+^**). A second SET event follows, restoring the chiral ion pair and affording the neutral cycloadduct (**pdt**).

Two distinct mechanistic scenarios were considered:

1. The photooxidant (**Pc** ^**+**^) of the chiral ion pair [**Pc** ^**+**^ **−** **X^−^**], after accepting an electron from **sub** and becoming a neutral radical (**Pc^·^**), remains part of the system (gray line in Figure 1b);
2. The now-neutral photooxidant (**Pc^·^**) is excluded from the model (black line in Figure 1b), under the hypothesis that it no longer stabilizes the reactive system.

The results demonstrated that neutral radical photocatalyst **Pc^·^** provides no significant stabilization to the [**Pc^·^**−**X**−**sub^·^** **^+^**] and [**Pc^·^**− **X**−**pdt^·^** **^+^**] complexes, thereby justifying the adoption of the reduced model (black) in all stereochemical analyses that follow.

The full model, including the substrate **sub**, the anion **X^−^**, and the cation **Pc** ^**+**^, was instead crucial for modeling the initial single-electron transfer event, postulated to be triggered by photoexcitation. We began our analysis by computing the electronic difference density between the ground state and the first singlet excited state of [**Pc** ^**+**^ **−X^−^]** within the TDDFT framework (Figure S2). The resulting density redistribution is entirely localized on the photocatalyst cation with no significant contribution from the IDPi anion, confirming that excitation is confined to the **Pc** ^**+**^ moiety. This result corroborates that **X^−^** does not interfere during the photophysical step and instead anchors the resulting **sub^·^** ^**+**^ and directs stereocontrol during the subsequent reaction steps. To assess whether the photoexcited chiral ion pair and styrene could give rise to a charge-separated radical ion pair via electron transfer, as proposed by List and co-workers, we examined the triplet spin state of the full system comprising all three species. This model offers a computationally tractable open-shell reference to explore whether a radical configuration with spatially separated unpaired electrons is accessible under the experimental conditions. Indeed, in the triplet state, we observed a clear spin density split between **sub** and **Pc** (Figure 1c), consistent with the formation of **sub^·^** ^**+**^ and neutral radical **Pc^·^** upon irradiation, validating the foundational assumption that open-shell species are involved following photon excitation. This outcome justifies the use of **sub^·^** ^**+**^ as the entry point for all subsequent ground-state reactivity and stereochemical investigations. Consistent with this assignment, the SET from **sub** to the photoexcited **Pc** ^**+**^ is strongly exergonic (Δ*G*°(SET) = −28.8 kcal·mol^−1^) and features a small Marcus barrier (Δ*G* ^‡^ ≈ 0.3 kcal·mol^−1^; see Section S3), confirming thermodynamic feasibility and a barrier-light step under the conditions employed.^1,50–52^

Finally, we note that although the overall transformation is exergonic, with the product more stable than the reactants, all the reactive intermediates along the catalytic cycle—aside from the initial [**Pc** ^**+**^−**X^−^**] ion pair—are energetically uphill (Figure 1b). Indeed, [**Pc** ^**+**^−**X^−^**] was the only intermediate detected and characterized in the original paper, which serves as a further confirmation of the accuracy of our computational protocol.^22^ Entry into the catalytic cycle is enabled by the formation of **sub^·^** ^**+**^ upon SET, which then proceeds through high-energy intermediates toward the product under the driving force of light. Once formed, the product does not compete with the substrate in the single-electron transfer to the newly photoexcited chiral ion pair owing to the relative alignment of their HOMO energies (see Figure S3). These results lay the necessary groundwork for investigating the stereochemical preferences that emerge in the catalytic cycle.

### 3.2 Origins of Enantio- and Diastereoselectivity

List and co-workers originally hypothesized that stereocontrol in the IDPi-catalyzed [2 + 2] photocycloaddition arises from a two-step mechanism in which the first C−C bond-forming step determines enantioselectivity, while the second, subsequent bond-forming step governs the observed diastereoselectivity.

We began our investigation of the stereochemical origins of this transformation by examining the inherent reactivity of the photocycloaddition couple **sub^·+^** and styrene (Figure 2). The reaction between **sub^·+^** and styrene follows a stepwise mechanism in which the first C−C bond-forming event is rate-determining, while the second step proceeds with a negligible barrier. These findings are consistent with earlier computations on related radical-cation [2 + 2] systems, which established a stepwise mechanism with the first C−C bond-forming transition state as the rate-limiting barrier.^28^ Notably, the activation barriers in Figure 2 demonstrate that the reaction of **sub^·+^** with styrene would proceed even in the absence of the chiral catalyst, underscoring the clear advantage of employing radical cation species generated via photoredox processes to facilitate challenging bond rearrangements.

**Figure 2:**
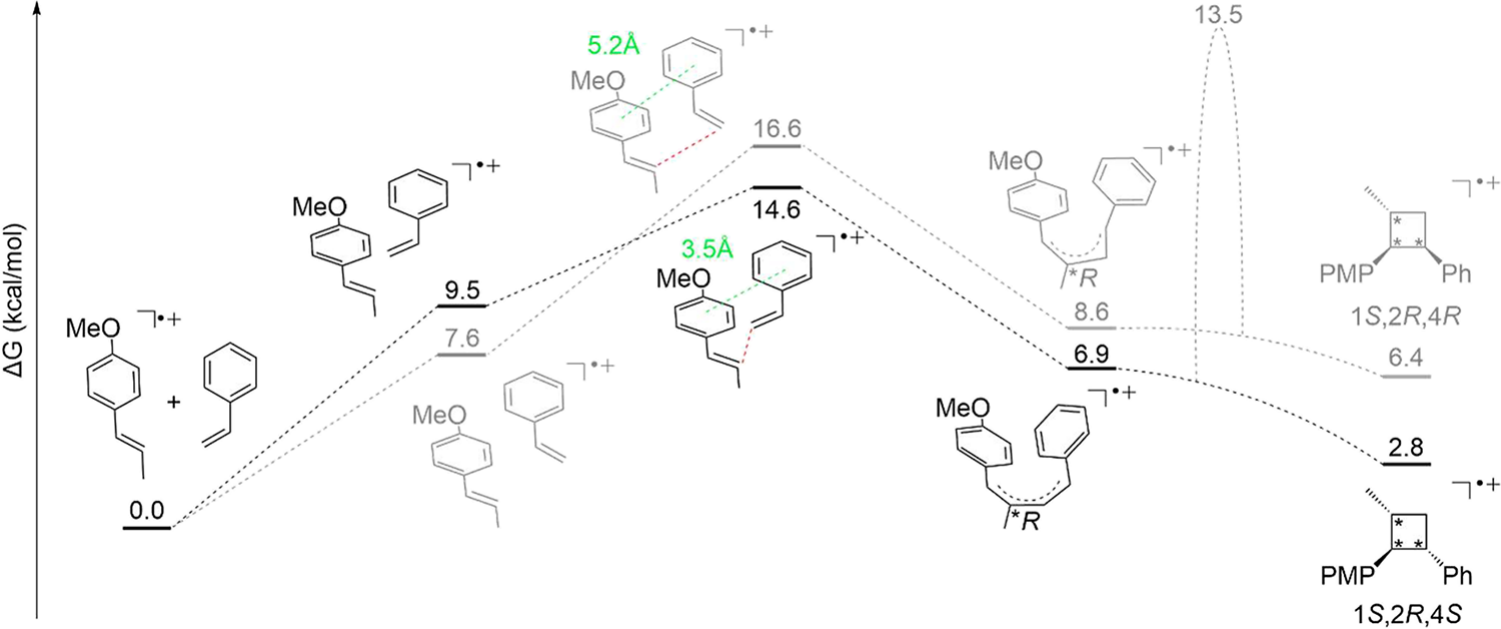
Computed free energy profiles (Δ*G*, kcal mol^−1^) for the uncatalyzed [2 + 2] cycloaddition of **sub^·^** ^**+**^ with styrene. The black pathway leads to the (1*S*,2*R*,4*S*) product observed experimentally; the gray pathway leads to the (1*S*,2*R*,4*R*) diastereomer. Intermediates evolve barrierlessly to product after the first bond-forming step. A high barrier precludes crossover between the two pathways. Red values denote the forming C−C bond distances in the transition states; green values indicate centroid-to-centroid distances between the aryl moieties.

Calculations of the two competing diastereomeric pathways reveal that the formation of the experimentally observed diastereomer (1*S*,2*R*,4*S*) is favored both kinetically and thermodynamically favored. This result demonstrates that the observed diastereoselectivity originates from the intrinsic reactivity of **sub^·^** ^**+**^ and styrene, as both the lower barrier and the more stable product also arise in the absence of the chiral counteranion. Notably, the relative orientation of the reactants in the first rate-determining transition state determines the preferred diastereomer, because any post-transition-state interconversion between radical intermediates—which could alter the stereoisomer formed—has a much higher barrier than the second C−C forming step, consistent with ref 28. Specifically, the rate determining transition state leading to the (1*S*,2*R*,4*S*) diastereomer features the aryl rings of **sub^·^** ^**+**^ and styrene positioned in a parallel, close-contact geometry with an interplanar distance of 3.5 Å—corresponding to a *parallel-displaced* arrangement (Figure S4). This arrangement has been consistently identified by computational studies as the most stabilizing aryl−aryl stacking motif in the benzene dimer.^53–56^ This interaction is further favored by the para-methoxy substituent on **sub** ^**·+**^, which was proven to enhance these types of stacking interactions.^57^ In contrast, in the competing transition state leading to the (1*S*,2*R*,4*R*) isomer, the aromatic groups are twisted and separated by 5.2 Å, precluding effective stacking and correlating with the higher energy. Importantly, all viable transition states consistently involve the formation of a new C−C bond between the carbon of styrene and the carbon of **sub** ^**·+**^ that is furthest from their respective aryl rings. Our attempts to locate alternative pathways, involving the more proximal olefinic carbons (e.g., head-to-tail pathways; see Figure 6a for representative head-to-head and head-to-tail orientations) invariably led to prohibitively high-energy rearrangements (Δ*G* ^‡^ ≥ 25 kcal mol^−1^), confirming that only this specific connectivity can support the stereodefined [2 + 2] cycloaddition under the reaction conditions.

To rationalize the origin of enantioselectivity, it is required an explicit model of the chiral environment provided by the IDPi counterion, **X** ^**−**^. To this end, we employed a multilayer ONIOM approach, treating the reactive pair of **sub^·^** ^**+**^ and styrene at the DFT level and the full structure using GFN2-xTB (see Section 2). This multiscale strategy allowed us to capture the full supramolecular environment comprising hundreds of atoms while preserving an accurate description of the reactive couple. We found that the first C−C bond formation proceeds through four distinct transition states, each defined by (i) a specific conformer of **sub** ^**·+**^ stabilized by its interaction with **X** ^**−**^ and (ii) the relative orientation of styrene approaching the opposite reactive face. Crucially, the two conformers of **sub** ^**·+**^ arise from rotation around the single bond connecting the PMP aryl group and the reactive double bond fragment (Figure 3), which defines the accessible face of the prochiral sp^2^ carbon distal to the PMP group, which is first attacked by styrene.

**Figure 3:**
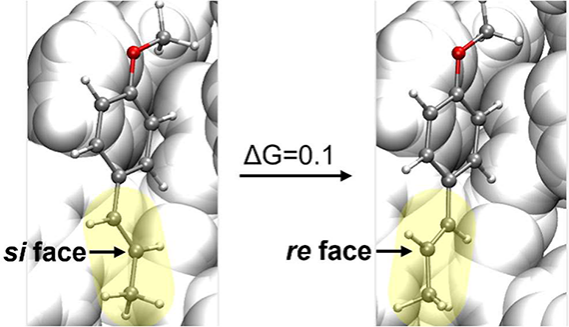
Depiction of the two conformers of **sub^·^** ^**+**^, defined by rotation around the PMP−alkene bond (rotating chain in yellow), which expose either the si or re face of the PMP-distal prochiral carbon. Δ*G* is reported in kcal/mol.

In one conformer, the *si* face of the PMP-distal prochiral carbon is exposed; in the other, the *re* face. Notably, the facial selectivity is inverted for the PMP-adjacent prochiral carbon of **sub^·^** ^**+**^. The two conformers give rise to the pair of pathways that ultimately lead to the enantiomeric products for which exceptionally high enantioselectivity was originally reported.^22^ For each face exposed to **sub** ^**·+**^, two approach trajectories of styrene are possible, resulting in four distinct transition states that capture the full range of enantio- and diastereomeric outcomes (Scheme 2). All viable orientations feature π−π stacking between the aryl rings and position styrene to maximize interactions with both **sub** ^**·+**^ and **X** ^**−**^. The origin of this preferred relative orientation and the role of **X** ^**−**^ in enforcing it are further discussed in the following section.

**Scheme 2:**
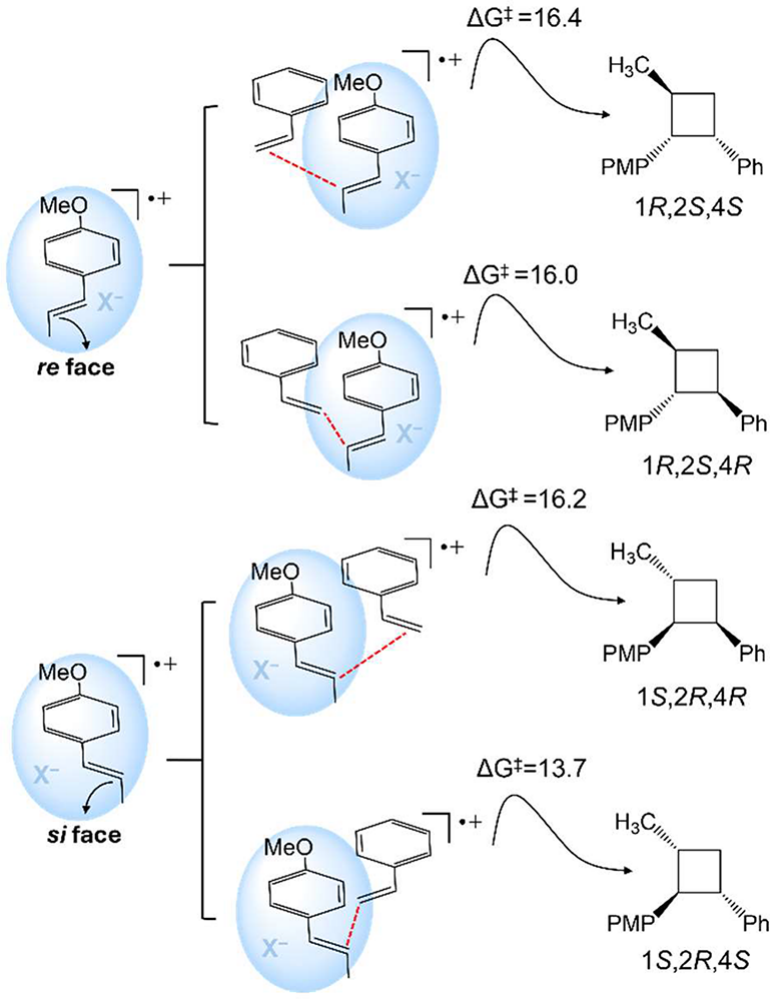
All Possible Relative Orientations between **Sub^·^** ^**+**^ and Styrene as a Function of the Exposed si or re Face of the PMP-Distal Prochiral Carbon of **Sub^·^** ^**+**^ ^a^

a Each orientation leads to a distinct stereoisomeric outcome. Computed activation barriers for the first C−C bond-forming step are reported for each pathway. The stylized blue shape denotes the counterion **X^−^**. Dotted red lines indicate the carbon atoms involved in the rate-determining step.

Among these four, the lowest-energy transition state involves styrene approach to the *si* face of the prochiral carbon and leads to the experimentally observed major enantiomer.^22^ In our calculations, this TS lies 2.3 kcal/mol below its enantiomeric counterpart, and 2.5−2.7 kcal/mol below the transition states leading to the other two diastereomers. The predicted ΔΔ*G* ^‡^ for the two enantiomers is consistent with the experimental er (97:3; ΔΔ*G* ^‡^ = 1.2 kcal·mol^−1^), supporting the proposed origin of stereocontrol. In line with the uncatalyzed reaction, the two lowest-energy transition states promoted by the chiral ion pair, leading to the experimentally observed product and its enantiomer, exhibit short aryl−aryl distances (∼3.8 Å) and a *parallel-displaced* alignment. In contrast, the higher-energy diastereomeric transition states show larger separations (>5.0 Å) and nonstacking geometries, indicating a loss of aryl−aryl stabilization. This suggests that while the chiral counteranion actively governs enantioselection, it simultaneously preserves the intrinsic diastereochemical bias encoded in the architecture of the reactive couple at the first bond-forming step. In all transition states, we find that ∼0.4 units of both positive charge and unpaired spin density are delocalized from **sub** ^**·+**^ onto styrene, based on Löwdin population analysis. This indicates that significant charge transfer and spin delocalization already occur at the first bond-forming stage. Importantly, our results indicate that the second bond-forming event proceeds with no appreciable barrier. This was observed consistently for both the catalyzed ONIOM model, including **X^−^**, and the uncatalyzed full DFT system with only **sub** ^**·+**^ and styrene.

To further validate that stereoselectivity is fully determined at the level of the first bond-forming event, even in the presence of the chiral counteanion, relaxed torsional scans along the newly formed C−C bond confirm that the energy required to reach the alternate configuration is significantly higher than that needed for direct product formation (Figure S5). We postulate that this rigidity is enforced by the aryl moieties on both **sub** ^**·+**^ and styrene, whose steric encumbrance restricts internal rotation around the key dihedral. Notably, this structural motif recurs across all product scaffolds reported by List and co-workers, suggesting that post-TS torsional rigidity is a general feature of this substrate class.

**Scheme 3:**
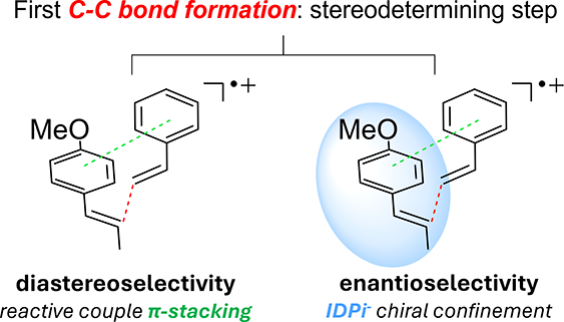
Dual Origin of Stereocontrol in ACPC: Diastereoselectivity Arises from the Aryl−Aryl Stacking of the Reactive Couple, while Enantioselectivity Is Imposed by Chiral Confinement of the IDPi Counteranion

Altogether, this analysis clarifies the mechanistic foundation of the stereocontrol in this transformation. As summarized in Scheme 3, the combined effects of the confined chiral environment provided by the IDPi counteranion, the stacking interactions and rigidity imposed by the reacting π-systems, as well as the irreversible nature of the first C−C bond formation, converge to encode both enantio- and diastereoselectivity within a single reactive event.

### 3.3 London Dispersion as Driving Force of Selectivity

Previous studies have suggested that London dispersion (LD) interactions play a central role in the performance of IDPi-based catalysts, particularly in shaping the stereochemical outcome through confinement-driven noncovalent interactions.^29,58,59^ Motivated by this, we applied our atomic decomposition of the London dispersion (ADLD) method to directly evaluate how attractive London dispersion forces contribute both quantitatively and spatially to the observed stereoselectivity of the system.^47,48^ Specifically, we compared the dispersion energy of each atom in the reactive couple across the two lowest-energy transition states for the rate-determining step, hereafter referred to as the major TS and minor TS (Figure 4a), leading to the experimentally observed (1*R*,2*S*,4*S*) product and its enantiomer (1*S*,2*R*,4*R*), respectively. The total dispersion energy difference between these TSs is 1.4 kcal mol^−1^, with a remarkable 1.2 kcal mol^−1^ localized exclusively on the atoms of the reactive couple. These findings strongly suggest that stereoselectivity arises from highly localized dispersion interactions of the reactive couple within **X^−^**, whose relative orientation is determined by how the system engages with the chiral environment shaped by the counteranion.

**Figure 4:**
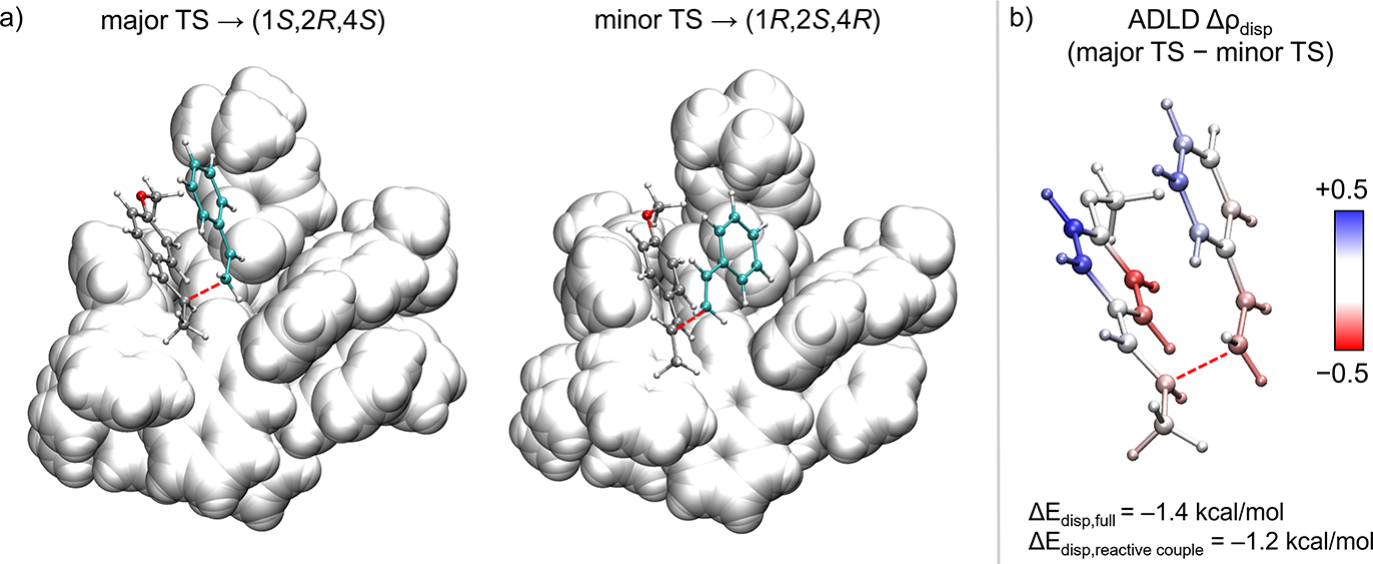
(a) Transition state structures leading to the major (1*R*,2*S*,4*S*) and minor (1*S*,2*R*,4*R*) enantiomers. Dashed lines highlight the atoms involved in the first C−C bond-forming step. **Sub^·^** ^**+**^ C atoms, silver; styrene C atoms, cyan. (b) ADLD density difference Δρ_disp_ between the two TSs, computed using the dispersion correction parametrized for the ωB97X-D3BJ functional and projected onto the structure of the reactive couple for the major TS. Red/blue indicate atoms with higher dispersion stabilization in the major/minor TS, respectively. The reported dispersion energy differences Δ*E*_disp_ refer to (major TS−minor TS).

To further dissect how individual atoms of the reactive couple contribute differently to the overall dispersion stabilization and how these differences arise from their distinct spatial arrangements within the chiral environment defined by **X^−^**, we analyzed the atom-resolved dispersion density difference Δρ_disp_ (Figure 4b). Notably, the carbon atoms directly involved in the bond-forming event are both more stabilized in the major TS, underscoring the role of London dispersion not merely as a passive background force but as a direct contributor that reinforces stereodetermining contacts.

Encouraged by these insights, we examined substitution on the styrene ring, as experiments generally show a drop in enantioselectivity with a dependence on substituent position. We adopted para-methylstyrene as a model: compact and electronically simple, it minimizes changes in polarizability and aromatic footprint, while still offering a controlled site for dispersion with the catalyst. In this case, the computed energy difference between the major and minor transition states decreases from 2.3 kcal·mol^−1^ with the unsubstituted styrene to 1.8 kcal·mol^−1^. Even though subkcal·mol^−1^ energy differences should be considered carefully, given the complex nature of the system, it is remarkable how the predicted decrease in er for this model substrate is fully consistent with the experimental trend reported in ref 22 for para-substituted styrenes (Figure 5).

**Figure 5:**
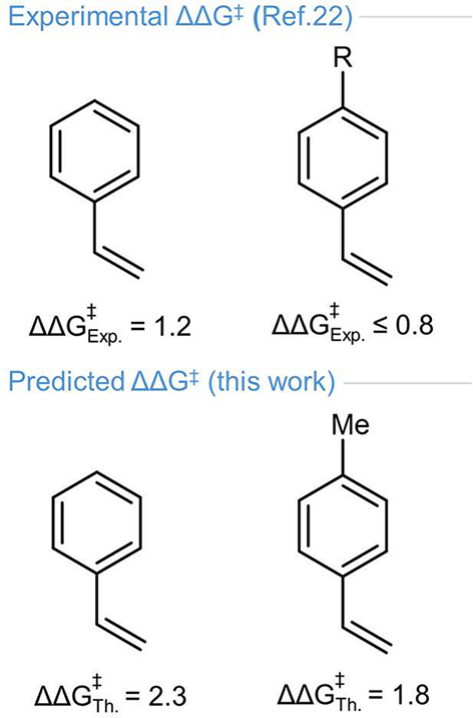
Effect of the para substitution of styrene on enantioselectivity. Upper panel: experimental ΔΔ*G* ^‡^ (ref 22 for styrene and for *p*-R-styrenes (R = *t*-Bu, Ph, Cl, I), illustrating the effect of replacing unsubstituted styrene with a para-substituted derivative in the reactive couple. Lower panel: predicted ΔΔ*G* ^‡^ for styrene and for *p*-Me-styrene, highlighting the same replacement within our model. All ΔΔ*G* ^‡^ values are reported in kcal·mol^−1^.

This effect originates from substitution-induced tuning of noncovalent interactions in the key transition states: the para position is oriented outward from the IDPi in the favored TS, while it points directly toward the counterion pocket in the disfavored TS (Figure 4 for styrene and SI for *p*-Me-styrene). For *p*-Me-styrene, ADLD analysis indicates a 0.6 kcal·mol^−1^ greater dispersion stabilization of the para substituent in the minor transition state, correlating well with the reduced discrimination.

Beyond the transition state, our analysis reveals that the IDPi anion already exerts stereocontrol during the prereactive phase by guiding the geometry of the encounter complex. While no reactant complex involving styrene approaching the **[X−sub^·+^]** ion pair is significantly more stable than the nonassociated fragments, the four productive approaches (Scheme 2) characterized by π-stacked alignment of the **sub** ^**·+**^ and styrene aryl rings and close olefin−olefin proximity consistently emerge as local minima. These structures lie within a thermodynamically accessible range of 0.6−2.9 kcal mol^−1^ above the dissociated state and serve as viable entry points into the reaction coordinate. In contrast, conformers where styrene approaches from the opposite face fail to converge to reactive geometries: geometry optimizations show that it is nonproductively drawn into the IDPi pocket, unable to position its olefin for C−C bond formation. To visualize the role of the IDPi scaffold in enforcing this organization, we computed a molecular dispersion potential (MDP) map of **X^−^** in [**X^−^**−**sub** ^**·+**^] (Figure 6).

**Figure 6:**
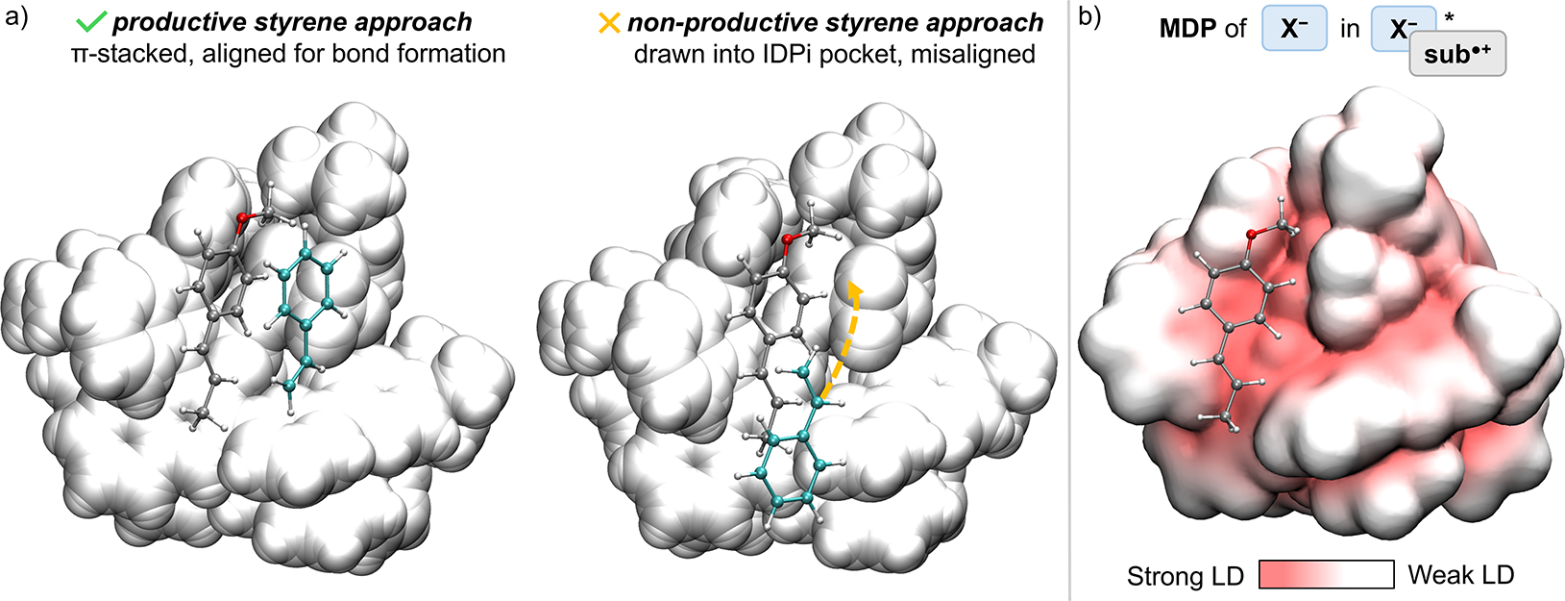
(a) Optimized geometries of prereactive complexes showing the approach of styrene (cyan C atoms) to **sub** ^**·+**^ (silver C atoms) within in [**X**−**sub** ^**·+**^]. Left: head-to-head productive approach; right: head-to-tail nonproductive approach. (b) Molecular dispersion potential (MDP) of **X** in [**X**−**sub** ^**·+**^].

The analysis reveals a confined dispersion-rich chiral pocket around the reactive site, which complements the steric architecture of the substrate and effectively directs styrene into a productive stereoelectronically favorable orientation. This highlights how the electrostatic and steric landscape of IDPi not only creates a chiral environment but also enforces a favorable approach trajectory, aligning the reacting partners for successful bond formation. The spatial steering effect of the catalyst, already manifested at the level of the reactant complex, anticipates the emergence of selectivity in the transition state and exemplifies the dual role of IDPi as both a confined chiral framework and a dynamic orienting element.

## 4 Conclusions

This work elucidates the mechanistic basis and stereocontrolling factors of asymmetric counteranion-directed photoredox catalysis, using a landmark radical [2 + 2] cycloaddition as a case study. To capture the subtle but decisive effects that govern selectivity, we established a computational protocol capable of accurately exploring the complex reactive landscape of such large ion-pair catalytic systems.

Our results demonstrate that both enantio- and diastereoselectivity originate from a single, early transition state shaped by the interplay between the intrinsic properties of the reactive pair (radical cation and styrene) and the chiral environment provided by the counteranion. Diastereoselectivity is dictated solely by the nature of the reacting partners, which direct the formation of a specific diastereomer through aryl−aryl-stabilized transition states. These aryl−aryl interactions, encoded in the architecture of the radical cation and styrene, are preserved across both catalyzed and uncatalyzed pathways, highlighting their intrinsic role in shaping the diastereomeric outcome.

In contrast, enantioselectivity is enforced by the IDPi counteranion, which, despite not interfering with the initial single-electron transfer, steers the reactive radical cation and stabilizes the transition state leading to the experimentally observed enantiomer. This occurs within a confined chiral pocket, where London dispersion interactions guide the system toward a single productive stereochemical outcome. Upon substitution, the reactive couple is reconfigured such that emergent geometry-specific contacts with the catalyst pocket may preferentially stabilize the minor transition state, reducing the enantiomeric discrimination. The use of ADLD and MDP analyses further enabled a physically interpretable understanding of how these noncovalent forces can promote or hinder asymmetric induction.

Our findings underscore a general principle: stereoselectivity in chiral ion pair-mediated radical catalysis emerges from the concerted action of substrate architecture and chiral confinement. While the substrate intrinsically biases the reaction toward a preferred diastereomer, the counteranion enforces enantioselectivity through spatial and electronic organization that is responsive to noncovalent interactions arising from substrate substitution. This dual contribution defines a transferable framework for asymmetric induction, opening new directions for the rational design of stereoselective radical transformations through the synergistic tuning of the catalyst and substrate.

## Supplementary Material



## Notes

Additional analysis and Cartesian coordinates of relevant structures (PDF)
