# Supplementary material for: Deciphering Asymmetric Induction in Photoredox Catalysis by Chiral Counteranions
Source: ACS Catal. 2025 Dec 19;16(1):766–74. doi: 10.1021/acscatal.5c07578 (PMC12772447; doi:10.1021/acscatal.5c07578)
Supplement: Supplementary file 1 [file cs5c07578_si_001.pdf]

Electronic Supporting Information (ESI)

Deciphering Asymmetric Induction in Photoredox Catalysis by Chiral Counteranions

Lorenzo Baldinelli,*[,a] Sofia Lerda,[a] Riya Kayal,[b] Frank Neese,[b] Filippo De Angelis,[c,d,e] Giovanni Bistoni*[,a]

[a] *Dipartimento di Chimica, Biologia e Biotecnologie, Università degli Studi di Perugia, Perugia 06123, Italy*

[b] *Max-Planck-Institut für Kohlenforschung, Kaiser-Wilhelm Platz 1, 45470 Mülheim an der Ruhr, Germany*

[c] *Dipartimento di Chimica, Biologia e Biotecnologie, Università degli Studi di Perugia and INSTM, Perugia 06123, Italy*

[d] *Computational Laboratory for Hybrid/OrganicPhotovoltaics (CLHYO), Istituto CNR di Scienze e Tecnologie Chimiche "Giulio Natta" (CNR−SCITEC), Perugia 06123, Italy*

[e] *SKKU Institute of Energy Science and Technology (SIEST), Sungkyunkwan University, Suwon 440−746, Korea*

*Email: lorenzo.baldinelli@dottorandi.unipg.it .

*Email: giovanni.bistoni@unipg.it .

Table of Contents

S1 Protocol validation via comparison with experimental UV-Vis spectra 3

S2 Excitation localization analysis of [Pc^{+}–X^{-}] 4

S3 SET energetics and MOs Alignment Analysis 5

S4 Aryl–aryl stacking in diastereomeric transition states 6

S5 Barrier to post-transition state interconversion 7

S6 References 8

S7 Cartesian coordinates 9

S1 Protocol validation via comparison with experimental UV-Vis spectra

To validate the computational protocol employed in this study, we compared the experimental UV-Vis spectrum of the ion pair [**Pc**$^+$–**X**$^-$] with TDDFT spectra computed using a range of functionals in combination with the def2-TZVP basis set on geometries optimized at the PBE-D3BJ/def2-SVP level. Among all tested methods, the ωB97X-D3BJ functional showed the best agreement with experiment, accurately reproducing all key spectral features. These results establish this protocol as a reliable framework for capturing the electronic structure of radical ion pairs and support its adoption for all high-level calculations in this work.

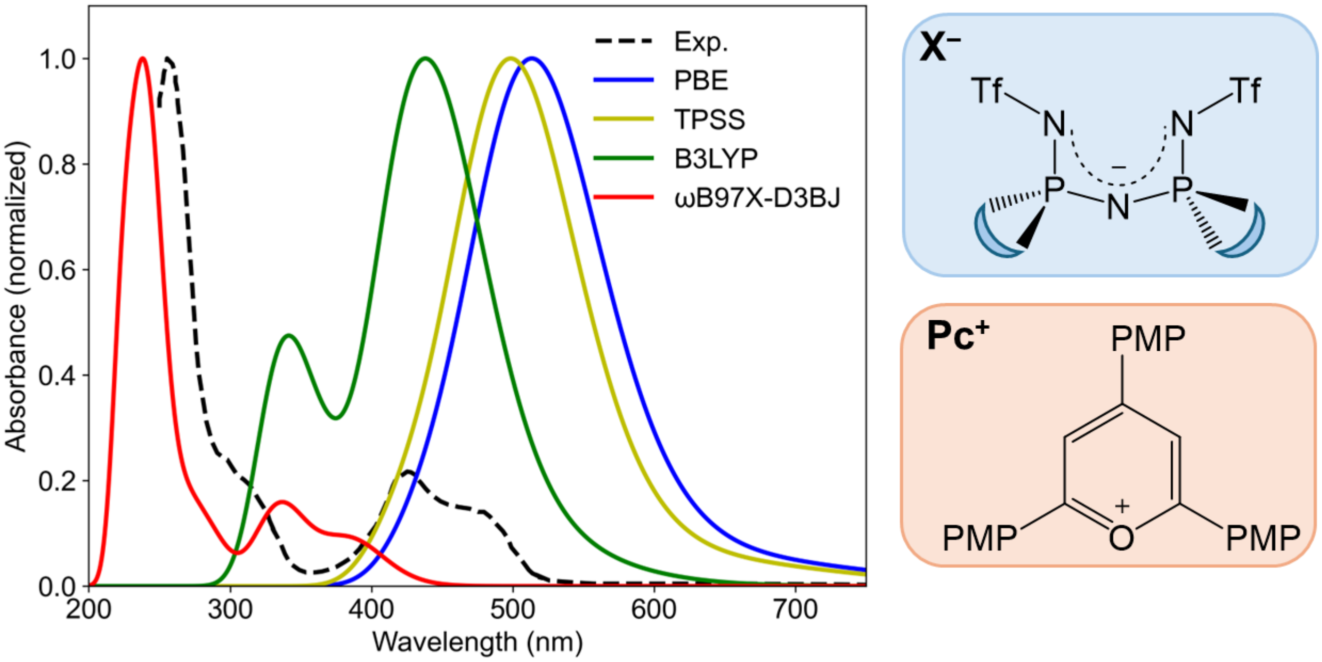

Figure S1. Comparison between the experimental UV-Vis spectrum of the chiral ion pair **Pc**$^+$–**X**$^-$ (black dotted line) and TDDFT spectra computed with various functionals (colored lines) using geometries optimized at the PBE-D3BJ/def2-SVP level. A schematic representation of the chiral ion pair structure is also depicted for clarity.

S2 Excitation localization analysis of [Pc⁺–X⁻]

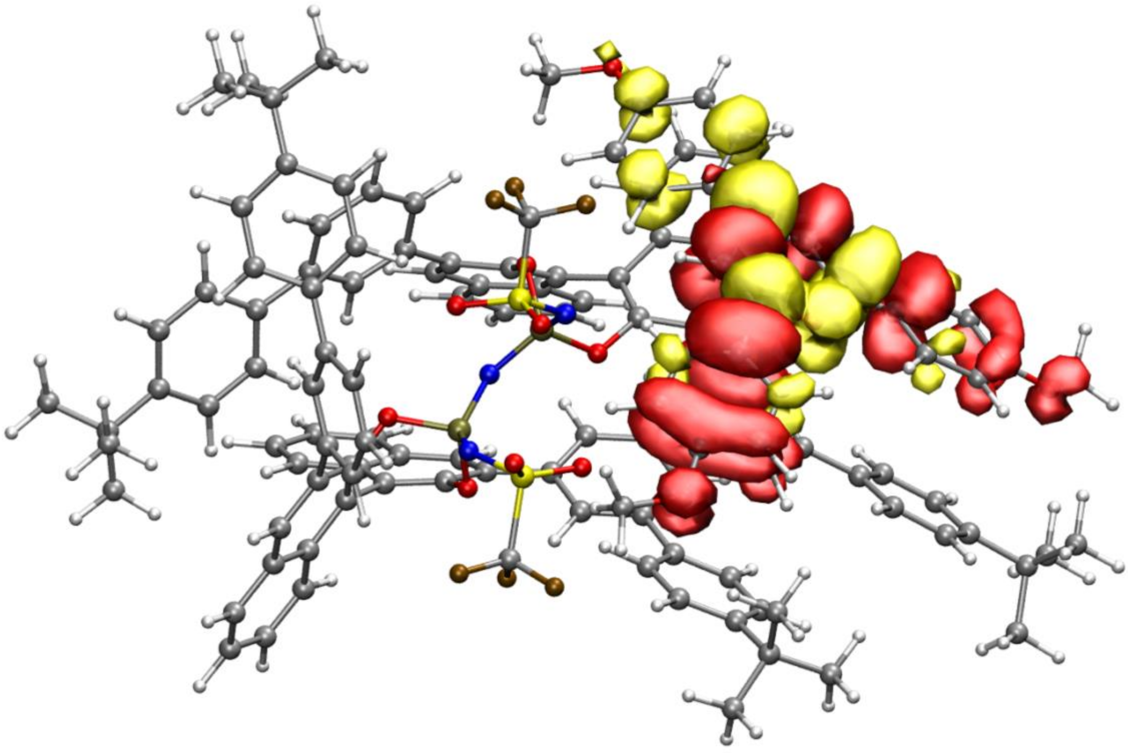

Figure S2. Difference density (S_1–S_0) of the [**Pc**$^+$–**X**$^-$] ion pair, computed using TDDFT with the ωB97X-D3BJ/def2-TZVP. The excitation is fully localized on the photocatalyst cation, further supporting the conclusion that the chiral counteranion IDPi does not participate in the photophysical SET process. Isosurface values: ±0.0005 au; red indicates electron depletion, yellow accumulation.

S3 SET energetics and MOs Alignment Analysis

SET energetics were evaluated by combining the oxidation strength of the substrate with the reduction strength of the excited photocatalyst, estimated as $E_{\mathrm{red}}(\mathrm{Pc}^{+*}) = E_{\mathrm{red}}(\mathrm{Pc}^{+}) + E_{0,0}$,[1] where $E_{0,0}$ was included as the TD-DFT vertical $S_0 \rightarrow S_1$ energy. Activation barriers were obtained within classical Marcus theory[2–4] using the total reorganization energy $\lambda = \lambda_{in} + \lambda_{out}$, where λ_{in} and λ_{out} denote inner and outer sphere reorganization energies, respectively. λ_{in} was computed by the four-point scheme for donor and acceptor;[5] λ_{out} was evaluated with a two-spheres dielectric model using radii obtained from CPCM cavity volumes.[6] Donor–acceptor separations were set to the contact distance derived from CPCM cavity radii ($R_{contact} = r_{donor} + r_{acceptor}$), the Marcus barrier $\Delta G^{\ddagger} = \frac{(\Delta G^{\circ}+\lambda)^2}{4\lambda}$ was found to be 0.3 kcal·mol^{-1}. In our case the reaction lies slightly in the inverted region ($|\Delta G^{\circ}| > \lambda$); since $E_{0,0}$ as vertical $S_0 \rightarrow S_1$ is an upper bound, sensitivity tests decreasing it by 0.1-0.2 eV yield barriers lower than those reported, reinforcing feasibility. All quantities were computed at the same level of theory and solvation as in the main text.

We also examined the alignment of the frontier molecular orbitals, specifically the highest occupied (HOMO) and lowest unoccupied (LUMO) orbitals, of the substrate (**sub**), the chiral ion pair (**Pc^{+}–X^{-}**), and the product (**pdt**). All species were modeled as neutral in their singlet ground states. As shown in **Figure S3**, the HOMO levels of the substrate and the chiral ion pair are nearly isoenergetic, with a minimal energy input of approximately 1.2 kcal/mol required for electron transfer. In contrast, the HOMO of the product lies significantly lower in energy, resulting in a much less favorable alignment and a higher energy requirement of about 9.1 kcal/mol. This analysis confirms that the product, once formed, is not electronically competitive with the substrate for single-electron transfer with the photoexcited catalyst, thereby supporting the selective nature of the catalytic cycle.

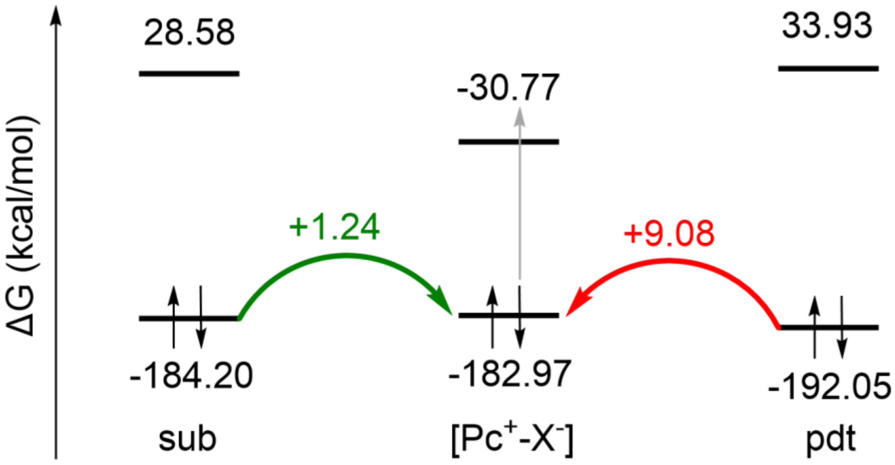

Figure S3. Energy alignment of the frontier molecular orbitals of the substrate, chiral ion pair, and product. Orbital energies and the energy required for electron transfer are reported in kcal/mol.

S4 Aryl–aryl stacking in diastereomeric transition states

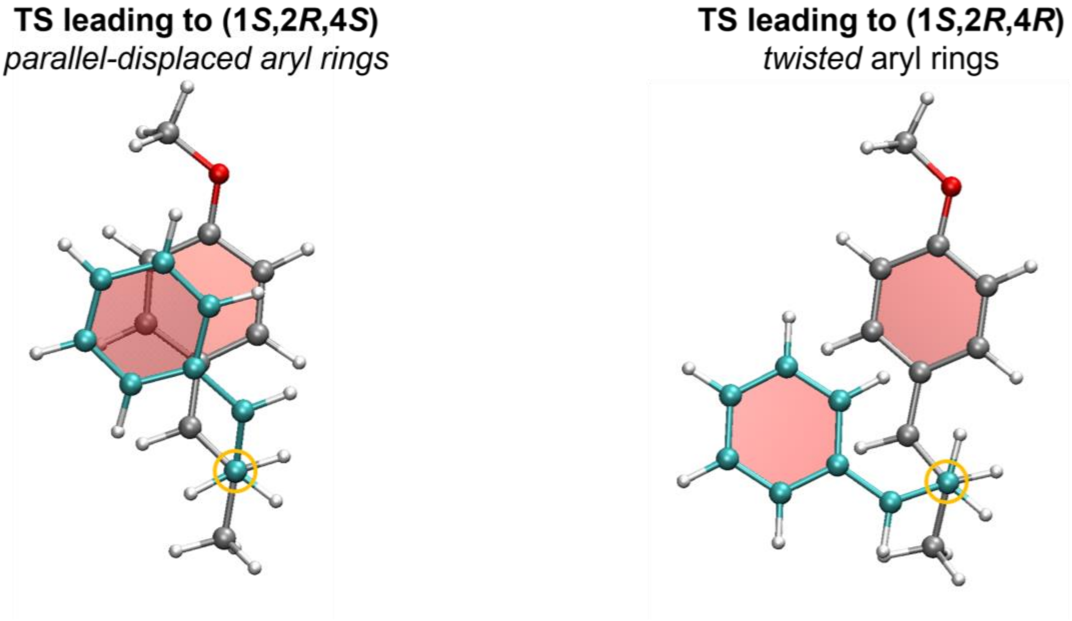

Figure S4. Comparison of transition state structures for the uncatalyzed reaction leading to the (1*S*,2*R*,4*S*) and (1*S*,2*R*,4*R*) diastereomers. The aryl rings of **sub**$^{•+}$ (C atoms, grey) and styrene are represented by red planes, highlighting the parallel-displaced aryl–aryl stacking (left) and the twisted, non-stacking arrangement (right). The two atoms involved in the C–C bond formation are circled in yellow and overlap in both transition states, perpendicular to the planes of the aromatic rings.

S5 Barrier to post-transition state interconversion

To assess the possibility of post-transition state interconversion between stereoisomeric products, we performed relaxed potential energy scans along the key torsional coordinate connecting the two geometries after the first C–C bond formation. In both cases, catalyzed (with **X**⁻) and uncatalyzed, the resulting energy profiles reveal a significant rotational barrier exceeding 20 kcal mol^{-1}. These results confirm that once the first bond is formed, the system is kinetically locked into its stereochemical outcome, rendering subsequent interconversion between diastereomeric products unlikely under reaction conditions.

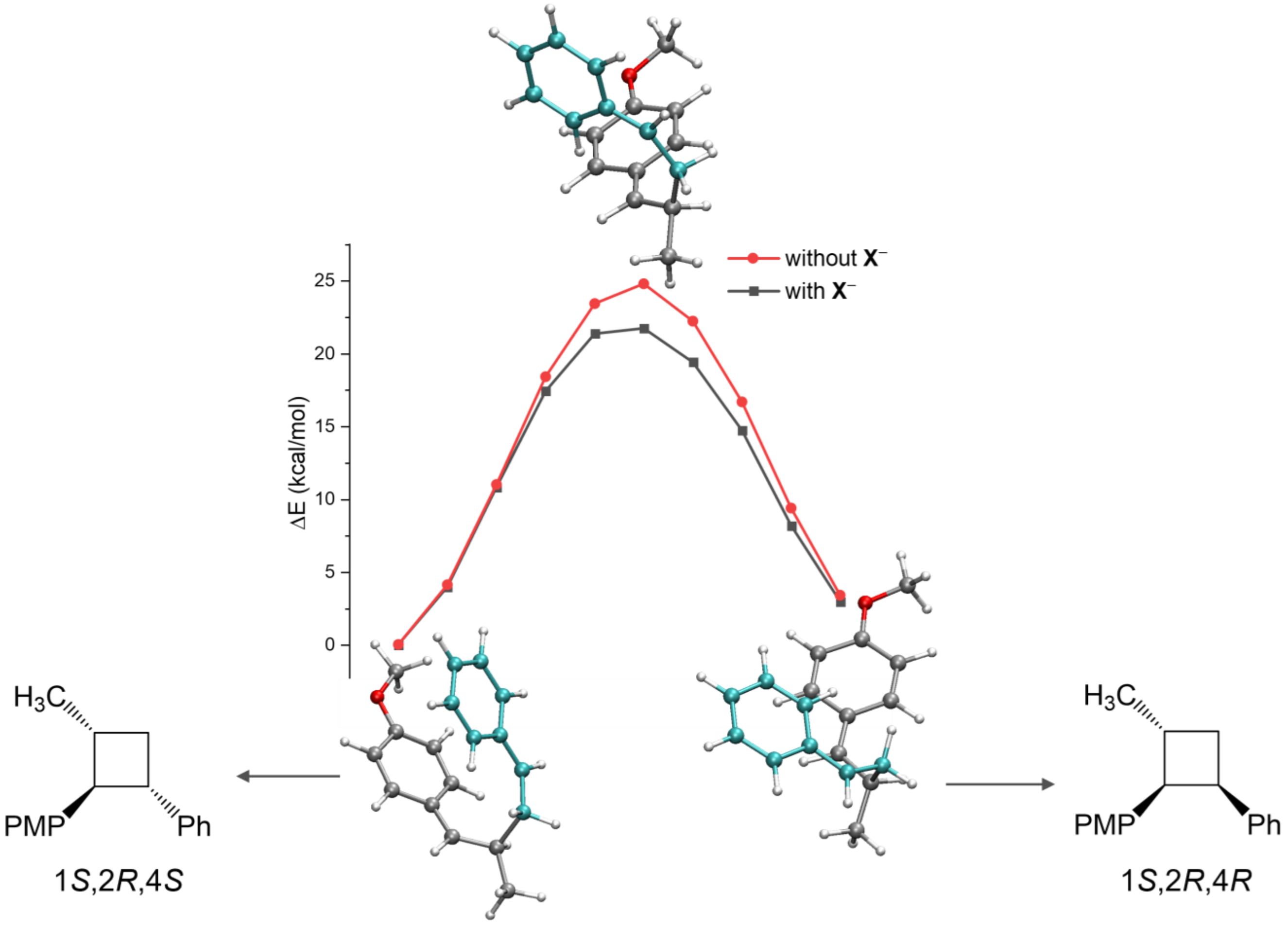

Figure S5. Energy profile along the torsional coordinate connecting two stereoisomeric intermediates formed after the first C–C bond between **sub•**$^{+}$ (grey carbon atoms) and styrene (cyan carbon atoms). Geometry optimizations were performed on the reactive core at the PBE-D3BJ/def2-SVP level, with single-point energy refinements at ωB97X-D3BJ/def2-QZVP, both in the presence and absence of the IDPi counteranion (**X**⁻), treated at the GFN2-xTB level when included.

S6 References

(1) Romero, N. A.; Nicewicz, D. A. Organic Photoredox Catalysis. *Chem. Rev.* **2016**, *116* (17), 10075–10166. https://doi.org/10.1021/acs.chemrev.6b00057.

(2) Marcus, R. A. On the Theory of Oxidation-Reduction Reactions Involving Electron Transfer. I. *The Journal of Chemical Physics* **1956**, *24* (5), 966–978. https://doi.org/10.1063/1.1742723.

(3) Marcus, R. A. Chemical and Electrochemical Electron-Transfer Theory. *Annu. Rev. Phys. Chem.* **1964**, *15* (1), 155–196. https://doi.org/10.1146/annurev.pc.15.100164.001103.

(4) Marcus, R. A. Theoretical Relations among Rate Constants, Barriers, and Broensted Slopes of Chemical Reactions. *J. Phys. Chem.* **1968**, *72* (3), 891–899. https://doi.org/10.1021/j100849a019.

(5) Amini, A.; Harriman, A. Computational Methods for Electron-Transfer Systems. *Journal of Photochemistry and Photobiology C: Photochemistry Reviews* **2003**, *4* (2), 155–177. https://doi.org/10.1016/S1389-5567(03)00027-3.

(6) Chaudhuri, S.; Hedström, S.; Méndez-Hernández, D. D.; Hendrickson, H. P.; Jung, K. A.; Ho, J.; Batista, V. S. Electron Transfer Assisted by Vibronic Coupling from Multiple Modes. *J. Chem. Theory Comput.* **2017**, *13* (12), 6000–6009. https://doi.org/10.1021/acs.jctc.7b00513.

S7 **Cartesian coordinates**

[X^{-}−Pc^{+}]

N	-0.46943352965601	-1.53950658029379	-1.05105036289557
S	-1.70534230719165	-1.70226627644833	-2.08758159884799
O	-1.28898281638257	-1.34035362982049	-3.46122746960901
P	-0.72213397471074	-0.98966681925258	0.45525627832236
N	-1.61962406986727	0.28190203740319	0.74987454942394
P	-2.10302249040785	1.61959571001777	0.04244763220585
N	-1.99494587263890	1.93956783757685	-1.52119215932949
S	-0.79799949629280	2.17861048870791	-2.54123954480638
C	-0.67298629008851	4.06999751606379	-2.59341365802080
F	0.36539514013065	4.41558860175715	-3.37998578873275
O	-1.27806880663715	-2.27197956808268	1.33081095487677
C	-1.41867613148059	-2.05395797260154	2.70224462913975
C	-0.26162656706135	-2.02955440108503	3.47616787573946
C	-0.37852116251662	-1.64673526947156	4.86295749600614
C	-1.68846495093528	-1.35125821704915	5.39337480301657
C	-2.83579013005261	-1.48115385488934	4.56067882661996
C	-2.73220724344076	-1.83516573165198	3.21926312415564
C	0.75097520243169	-1.47778781337633	5.71884743426250
C	0.59708100659788	-1.04646509424843	7.02984223346121
C	-0.69428435849037	-0.77199163198865	7.55485651891147
C	-1.81310952388792	-0.92312663594874	6.74939738851787
C	1.05486200305080	-2.35824769782289	2.86138467979065
C	1.54685876940712	-1.64263786963705	1.76112068262285
C	2.90880418739517	-1.75567066138139	1.31233642669176
C	3.69597008387842	-2.72134961355990	1.94117701126568
C	3.19892821098684	-3.59615900157939	2.93912516508653
C	1.85091259802935	-3.42489516227545	3.41160930686785
C	4.00286279463997	-4.64611933820751	3.47518105759329
C	3.48914623380688	-5.51896787101890	4.42227132185325

C	2.14659549296104	-5.37311646993954	4.86395734940354
C	1.34844230520662	-4.34661028233031	4.37642863381903
C	3.52478900193985	-0.85832353809384	0.29856819324985
C	4.90089449122018	-0.54301842115044	0.41036091518102
C	5.55901342518709	0.29625243962081	-0.51164410712172
C	4.79862816028459	0.83641254592975	-1.57261491253776
C	3.44030081384574	0.54460784235699	-1.69311409193132
C	2.80258734803535	-0.30459601519268	-0.78166264983338
O	0.73581044910491	-0.68522978178157	1.15772622288145
C	-3.91985643214641	-1.97517026659463	2.34182233603506
C	-4.91771789481912	-0.98329352319665	2.34294098623694
C	-6.04897852890393	-1.06796837702570	1.50098912558650
C	-6.16436479006348	-2.19973370355129	0.65976452565583
C	-5.18617070560832	-3.20348808308557	0.66621926193396
C	-4.06471577621742	-3.09830437067937	1.49675223685703
O	-1.50467623811112	2.91673554640250	0.89723376691970
C	-1.84900173409475	2.95530449564903	2.25080240438288
C	-0.86836967109158	2.59282454975191	3.23287316041977
C	-1.30916447869220	2.52919614621118	4.55643042532925
C	-2.65581368234034	2.77529183529853	4.93299639006329
C	-3.60721736918940	3.19716687168768	3.93554033730134
C	-3.16251862325158	3.32413005112314	2.57282215997549
C	-4.95892428140584	3.41548153266542	4.33744827042385
C	-5.35088424395025	3.22582996251499	5.65570841029620
C	-4.40849869723269	2.82217512197456	6.64043288057948
C	-3.08590281353168	2.60567821608970	6.28410976090233
C	0.55410746069815	2.29015278991254	2.91584775087876
C	1.24882223264251	1.35796568094851	3.72152931227237
C	2.60909408606375	1.10536058073255	3.51463132063565
C	3.30544915437973	1.76936133653636	2.49771143014061
C	2.64152746335221	2.69622311411222	1.66155059646470
C	1.26680664132585	2.94339721267401	1.88515344898071

C	-4.06651288698944	3.79842179771827	1.48505293850188
C	-4.29551613149270	2.97661699692041	0.37926704688869
C	-5.09459727058342	3.36452524001038	-0.73921437960946
C	-5.69547476521061	4.61761856190813	-0.69374173214196
C	-5.50854223531991	5.50414589746900	0.40402177854323
C	-4.66161560829106	5.10988291661625	1.50461482629794
C	-4.42578980393065	6.05349362124487	2.54938991472974
C	-5.03454524765544	7.30109155056117	2.53562894374379
C	-5.90312489860087	7.67093965020595	1.47342857563890
C	-6.12647292222026	6.79071912438708	0.42536287712391
O	-3.71158687204722	1.70824129721576	0.38185974438052
C	-5.22555988410831	2.48508727130447	-1.93313594972545
C	-4.68460843441244	2.90883162507696	-3.16477184947496
C	-4.70380764297991	2.05345464451417	-4.27503556872562
C	-5.27620082598819	0.77769306816236	-4.17334292619194
C	-5.87010970815242	0.35680017308536	-2.96397363624343
C	-5.83499867733771	1.21909055038978	-1.84848056624296
O	-1.23578613442818	1.84207981436347	-3.91686257868383
O	0.53586691167754	1.71480447519898	-2.07946392477481
O	-3.01514337705052	-1.22027103316295	-1.57785761915132
C	-1.92193862926891	-3.58122096288275	-2.20212082045238
F	-0.78784456416992	-4.16232969532799	-2.63459977314339
F	-2.25443697464545	-4.10405403137100	-1.01282555283358
F	-2.89936587114627	-3.84999215001279	-3.08231722622723
F	-0.47311942114068	4.57379913785451	-1.36577741031728
F	-1.79195613786542	4.60228017163384	-3.10291289421143
C	-7.04440077036596	0.03307535515687	1.44290103572987
H	-4.78534856293800	-0.11155292602201	2.99962897530265
H	-5.29027751688974	-4.06457680000230	-0.01153573596618
H	-3.29667895470459	-3.88376070921198	1.49349966593938
H	1.74557151859789	-0.54104744505642	-0.93462817850903
C	6.99339517244167	0.64966869297333	-0.36221892862315

H	5.47307336421430	-0.95177529839439	1.25439573952017
H	4.36446709298285	1.53660205068668	2.31733590854706
H	0.71303726976349	0.80358886737014	4.50284482316041
H	0.74929117085435	3.68050391511255	1.25749972985511
C	3.37265073258004	3.34716277537935	0.54422876272917
H	-4.19665174798661	3.89128488236308	-3.22988378461438
H	-4.24734759776672	2.37853732310855	-5.22211946479774
H	-6.26052333120779	0.88385669818808	-0.89195668662231
H	1.75742171309440	-1.67090004855600	5.32345012193788
H	1.48522209367803	-0.90971587685409	7.66529126873263
H	-0.80262653421850	-0.43222562968065	8.59599083620542
H	-2.81933031801871	-0.70201891167046	7.13759512614317
H	-3.82989879860131	-1.29726295996802	4.99618227349946
H	0.30878346597194	-4.25120252163791	4.72002007996047
H	5.03505671026730	-4.75661159017296	3.10682527566661
H	4.74187284535326	-2.84379448495977	1.62974091118325
H	-5.69997411486352	3.72419568837671	3.58859218515996
H	-6.40248078078778	3.38547318799401	5.93846901900263
H	-4.73261711381953	2.67814797262416	7.68228460277868
H	-2.34574756783198	2.28347666136879	7.03276051877666
H	-0.59417299230910	2.26930930908776	5.35071820791604
H	-3.74849651592491	5.78467733393959	3.37158480247954
H	-4.83803754210724	8.01183138930127	3.35272577396223
H	-6.38503608327889	8.66033175379743	1.47846165986728
H	-6.78062309803481	7.07111669681118	-0.41477082740374
H	-6.32914674953455	4.93986308352345	-1.53431902986400
H	4.11206238841137	-6.33320551102909	4.82245434616285
H	1.73088136055266	-6.08586230495196	5.59218998094918
C	-6.49502353037327	-0.98571100781913	-2.84661802862892
H	2.84948940695359	0.98956013066823	-2.50349597004510
H	3.12514611611233	0.36060506059453	4.13984608400518
H	-7.00375070837636	-2.28093879547197	-0.04348176419083

H	5.26193078072919	1.53024856743699	-2.28427454428311
H	-5.28277760466445	0.10098922605439	-5.04168504022411
C	2.70887824076148	3.67092480093610	-0.65927336749062
C	3.40733899680672	4.16974271359273	-1.76220115833585
C	4.80454922659763	4.37708715781609	-1.72494767148322
C	5.45912985736186	4.09426729818450	-0.50826551963493
C	4.76152821278866	3.59059266247858	0.60020616344006
H	1.64045870594157	3.44692659819810	-0.76227756890573
H	2.84566536598745	4.36489828286887	-2.68815631764892
C	5.54025923471507	4.86365397396483	-2.98531677549853
H	6.54287052008906	4.24807700977514	-0.41374566531686
H	5.31945379013563	3.37391201374382	1.52412705864643
C	7.74983190266757	1.10707149796993	-1.46725035701035
C	9.09085468986800	1.47630356534259	-1.33152169793547
C	9.76135289148349	1.40212243758841	-0.08925032611619
C	9.01194222952980	0.93400678971742	1.00935505879746
C	7.66165580927717	0.57149037358422	0.87835756734242
H	7.28565896613247	1.16205230277639	-2.46273935044775
H	9.63057854764847	1.82750814245722	-2.22436912298041
C	11.22980231135733	1.84430843920804	0.01744703909480
H	9.47580789362871	0.85864419454085	2.00264493325071
H	7.11397281032978	0.24393882795578	1.77496719002708
C	-7.82631891335697	-1.13710833142717	-2.40293736008936
C	-8.38823273235333	-2.41017903075088	-2.24536380715402
C	-7.64488980642359	-3.58561340824928	-2.50165802287173
C	-6.32124676454245	-3.42305827556666	-2.96071125515088
C	-5.75945234528317	-2.15000133841630	-3.14206991896951
H	-8.42457624881201	-0.24234897099114	-2.17294974624376
H	-9.42935395332135	-2.48648475894595	-1.89474789921247
C	-8.27836274920725	-4.96429092702224	-2.24770127960741
H	-5.69356325965477	-4.30109673102275	-3.16481197393680
H	-4.71163844109465	-2.05514179586521	-3.45919174711948

C	-6.71165944410655	1.33713011775545	1.86685910453839
C	-7.59558488139310	2.41121529419894	1.70428132411989
C	-8.86418267479190	2.23994906074729	1.11605162740695
C	-9.21620884112842	0.92452828819565	0.73420390330516
C	-8.33591609501224	-0.15101394162717	0.89453158616498
H	-5.71974970668795	1.53440124126759	2.29251814339860
H	-7.26225733246639	3.41117102299843	2.01499983505582
C	-9.81851827480150	3.41299782558345	0.84443919556043
H	-10.20258923216800	0.73054302083041	0.28551902816307
H	-8.65950107758920	-1.15283054977554	0.57800117161832
C	4.95461319095488	6.22584241987179	-3.42264282369137
C	5.33790956841592	3.83392462916631	-4.12334613121612
C	7.05121588061435	5.03074077833832	-2.74611966386425
H	5.07781736332779	6.98458763947828	-2.62296603182870
H	3.87408834638184	6.14936305632661	-3.65698130347108
H	5.47398942362740	6.59405182773326	-4.33163277679720
H	7.54319294785499	5.36717323698070	-3.68106497358342
H	7.52686385228927	4.07717217005751	-2.43889790508086
H	7.26209860885351	5.78751091546769	-1.96341526446994
H	4.26562324836349	3.65684092785228	-4.34182214758123
H	5.79534324333717	2.85761381421640	-3.86188177431921
H	5.81995347247545	4.19364501201959	-5.05593408513634
C	-8.67523020028721	-5.06564033481021	-0.75609917866152
C	-7.31115357958899	-6.11564708253104	-2.57221269746116
C	-9.53851881633261	-5.11759917525550	-3.12915423480666
H	-7.80889155905020	-7.08684454600353	-2.37649544114096
H	-6.39669944550050	-6.07503188126862	-1.94588945720690
H	-6.99964989800731	-6.10471214905499	-3.63629502863647
H	-10.28884591768651	-4.33159540760863	-2.91154489918050
H	-10.01822585607155	-6.10226077587910	-2.94985428886041
H	-9.27746531040553	-5.05246328129429	-4.20523480501078
H	-9.40659784650316	-4.28234094073883	-0.47343064173130

H	-7.78621772951923	-4.95143777407963	-0.10290921311733
H	-9.13542236349106	-6.05329157303334	-0.54518762323660
C	11.80960445667363	1.59611501767100	1.42063139910011
C	11.31435979521054	3.35789715022545	-0.29161419687643
C	12.08094681298597	1.06252447438048	-1.00993347651913
H	10.92869317024524	3.58670554934978	-1.30536014356969
H	12.36781099773337	3.70340232011068	-0.24020884771435
H	10.72148914365916	3.94471965650214	0.43954717445688
H	12.87167391640656	1.91307038333192	1.44692342244328
H	11.76993337583259	0.52314250500039	1.69760053939265
H	11.26942110592745	2.17231929057322	2.19871260176332
H	13.14645556100619	1.36207684951734	-0.93140105803362
H	11.75396773165122	1.26069882868582	-2.05019212310769
H	12.00691393494914	-0.02865660634073	-0.83249456347810
C	-9.30333162169068	4.72985805199450	1.45207191827015
C	-11.21058964582249	3.11027306755983	1.44217221789983
C	-9.93931845706291	3.59249685794350	-0.68780064968573
H	-10.33326092539369	2.67774585619155	-1.17462383417835
H	-8.94915832992861	3.81777164702199	-1.13457036107018
H	-10.62585022144886	4.43148794071528	-0.92582081523653
H	-10.02831146029137	5.54339204219335	1.24798259131694
H	-8.33018921486717	5.03407191066496	1.01879396293471
H	-9.18102670441338	4.65287446770515	2.55162429896051
H	-11.14803300754030	2.97360507073642	2.54115315446993
H	-11.66024716085393	2.19480485166018	1.00893913420268
H	-11.90334014017549	3.95276181482793	1.23878411063074
H	0.60787203554642	2.34128447608484	-5.38235638404349
C	1.08251939128798	2.12029991450594	-6.35926405451790
H	1.17667271985063	3.04736801667290	-6.95236847240176
H	0.51940493697498	0.07574772958891	-4.91370435019686
C	1.57027376545915	-0.23264061544898	-4.84212257468258
O	2.41464364170451	1.62206625053371	-6.19465018377281

H	-0.92582624872175	-4.83564841137443	1.09379576522338
C	0.07808622020083	-5.24755733345203	0.94284000799588
C	1.89147800064930	-1.32920777780701	-4.04220745415341
H	1.06258795647867	-1.84080263111312	-3.53522198187953
C	2.60517749785167	0.50687056798242	-5.46397540641108
C	0.93184366822828	-4.62208772257816	0.03519880535545
C	0.53468404016498	-6.36096439787273	1.68096868440740
H	0.57201872548548	-3.72336527075309	-0.48282833298163
O	-0.20433152723774	-7.01085806074044	2.60512848134328
H	1.62958040481258	-3.43465831824512	-2.30742411472150
C	3.23779463734782	-1.71225283861627	-3.82412373086707
C	2.69623868780352	-3.47120933202402	-2.06119220947910
C	3.95153824627587	0.08427376971648	-5.31839047993841
C	2.26199079112397	-5.06397181021402	-0.15861816498675
C	1.85034459480496	-6.84382358705325	1.47107588175265
C	3.57532337303163	-2.68022209010977	-2.79481323009909
C	4.26061684546546	-0.99667493656557	-4.50415925054266
H	0.45124196669499	1.39061696319061	-6.90906591895090
C	3.16982423332552	-4.30173453908702	-1.01584573344954
C	2.69922833642690	-6.19636476633895	0.58091628549008
H	4.74025643368077	0.65548499055477	-5.82911856533176
H	2.18746681104558	-7.71923881296051	2.04412643299187
O	4.89923468151303	-2.70408629155543	-2.49084747276953
C	4.56834496374650	-4.29982868278778	-0.77192250750565
H	5.31212281596136	-1.27731253152960	-4.35933635094368
H	3.71964746663062	-6.58341617663214	0.44544697426531
C	5.40615223259877	-3.45255616040710	-1.47819840286186
H	4.99349822974603	-4.90147449536396	0.03893551833000
C	6.81702962224012	-3.20269490583468	-1.21210210005837
C	7.40109213220645	-3.60843015437609	0.01215982570013
H	6.80339229282583	-4.14872491866873	0.76048912734032
C	7.62116155213736	-2.48368381330899	-2.13473677451332

H 7.19134153058207 -2.15546669320990 -3.09064678830989

C 8.72647815719574 -3.29832640953302 0.32176103415987

H 11.45989691212930 -3.61118496517642 1.03096351873364

C 8.94668114596864 -2.18368864311343 -1.84353324889511

C 9.51287217921958 -2.57565251615288 -0.60638362424948

H 9.13776588979842 -3.60811360923425 1.29100031781159

H 9.56941946242121 -1.62151166769587 -2.55336526519672

O 10.79663022963316 -2.21619862889641 -0.39646095336274

C 11.40275106756140 -2.51535011397899 0.86105117821001

H 10.84602271008187 -2.03998703118026 1.69619507357921

H 12.42298832730425 -2.09436847430708 0.81594030557048

C -1.51276820344674 -6.51972805571632 2.89729769675000

H -1.91583061337441 -7.17264969056950 3.69197210888977

H -2.17413197054709 -6.57079716073008 2.00623062496246

H -1.47651989446299 -5.47087906825502 3.26242171148959

[X^{-}–$sub^{\cdot+}$]

N 0.25616713532370 -0.79314748926853 1.87867335004008

S -1.26590061864730 -1.27969455672713 1.96452077992809

O -1.32559432352087 -2.66905475989981 2.47152502733384

P 0.92517633748996 0.08718977487361 0.71555301970831

N 0.16483246695685 0.78684147540952 -0.48199107890703

P -0.92673934074114 0.87437359436121 -1.63329138003457

N -1.33788718107126 -0.31485980809824 -2.62346142601776

S -0.89921768336617 -1.85301717951637 -2.51561469455292

C -0.04208537133989 -2.09261154444504 -4.20861536423079

F 0.92222976514794 -3.01959711979510 -4.09775084363497

O 1.74273340644922 1.25357967480267 1.58397808172557

C 2.68548509998601 2.03189526542715 0.93729623902042

C 3.87983483269040 1.43822570073077 0.47490033941668

C 4.76854963637250 2.23733576563634 -0.33299731669061

C 4.51522572755353 3.64827557743889 -0.46358827333301

C 3.40700966417154 4.22077989785529 0.21863790621566

C	2.46825695108493	3.44398481000602	0.89911979555452
C	5.87262579116825	1.67806776733911	-1.03545569249172
C	6.70010082325624	2.47860835010906	-1.81424182832374
C	6.46300364818168	3.87255394099696	-1.92389861546872
C	5.38540420447529	4.44640070245447	-1.26053224148492
C	4.25364362334492	0.07966572652888	0.93947495996259
C	3.34615561966233	-0.98930657119400	0.83528620670976
C	3.59679285711766	-2.28303378174701	1.40008518585473
C	4.85992589854527	-2.48356666622060	1.96600060307557
C	5.82770554839262	-1.45627904093808	2.08382830935123
C	5.51493944690786	-0.13136624350846	1.61740703214131
C	7.07721711015286	-1.70844336764281	2.72571308789844
C	7.98458606195175	-0.68134730923062	2.94472804877625
C	7.65441283932836	0.63744292736163	2.54307314149365
C	6.44914360594451	0.90657907731967	1.90138654368535
C	2.61281788008726	-3.39762982902193	1.49666533498705
C	1.74376784644391	-3.77790683279053	0.45490861665309
C	0.87859952414359	-4.88800602787814	0.58611576776888
C	0.86367435914483	-5.60074580506165	1.80613498722304
C	1.71038875008785	-5.22416513412671	2.85587494873006
C	2.58529397729795	-4.14264331560424	2.70150025708631
O	2.19011860068018	-0.77414307830465	0.10206853812911
C	1.39341283459645	4.11000479069228	1.68595999903428
C	0.03223891856866	3.75082070051427	1.61069911043092
C	-0.95311617911901	4.43292313508181	2.36404918101774
C	-0.53161764568632	5.46289092190350	3.23792009416209
C	0.81589355894803	5.83215515952185	3.31810074010886
C	1.77419471612520	5.17452153006576	2.53701715321974
O	-0.50746187283289	2.08543917171599	-2.69032059554868
C	-0.35009858864112	3.37783242659062	-2.20696479842624
C	0.93452252024298	3.99006341789253	-2.34701855606215
C	1.09101991875434	5.28097283260894	-1.84134555224755

C	0.04143080664891	5.96382238361387	-1.17114535065512
C	-1.26932888858770	5.36782912392500	-1.12564872284260
C	-1.47426195300196	4.08241829213815	-1.74559550474825
C	-2.31111021938732	6.06754511636146	-0.45358226452593
C	-2.07306570442784	7.30170048395774	0.13814476578851
C	-0.78495638157860	7.89569418324138	0.08107870150329
C	0.25350743293384	7.23605015288458	-0.56169859865997
C	2.02843887643969	3.36413999118543	-3.13715311161959
C	2.64262625248126	4.14338382752135	-4.14546660319595
C	3.67342181809121	3.60453855203315	-4.92631517777307
C	4.11515488125874	2.29439872612481	-4.70420578584048
C	3.53550354097690	1.49724229102862	-3.68949971714964
C	2.48232030850439	2.04717967029227	-2.92286586036103
C	-2.83338512817758	3.56423148004644	-2.05146830058252
C	-3.18873466931104	2.24623781806769	-1.73322906627469
C	-4.43479491522654	1.65879565308190	-2.12778150451102
C	-5.36022109348746	2.49978935172512	-2.74747882301846
C	-5.07003395928270	3.84667014293196	-3.08599144323662
C	-3.76198374042877	4.37962535210434	-2.80297209510283
C	-3.44523363596897	5.67213038850666	-3.32018393364025
C	-4.39479482194319	6.42765439141663	-3.99604456914647
C	-5.70982042519921	5.93041817453419	-4.19747726991128
C	-6.03405533917686	4.65533300869335	-3.76077308282472
O	-2.29864782033109	1.50157233243589	-0.96849077022059
C	-4.78478596208806	0.21639770859128	-1.99886161793582
C	-5.35566406647909	-0.41870825468667	-3.12875124450860
C	-5.76603870783748	-1.75607243675393	-3.06289017853885
C	-5.61856976791834	-2.48054499174181	-1.87352892245767
C	-5.03687403217840	-1.88018608100662	-0.73404610795262
C	-4.61843770739333	-0.53230820975477	-0.81760357746516
O	-2.07887004439122	-2.74121395292824	-2.56505844271729
O	0.16076972746697	-2.10994390461238	-1.50634827340553

O	-2.09927570485657	-0.89870221245888	0.79765680902659
C	-1.91134620945452	-0.21397864759091	3.42623581909459
F	-1.77583465256262	-0.89184757277141	4.58361738938018
F	-1.20958562731270	0.92718133595452	3.52212276482225
F	-3.20342817918701	0.08459769399130	3.25492076566531
F	0.51284881084769	-0.94676073453651	-4.62775155662253
F	-0.93773853552117	-2.50330153838865	-5.11960849562546
C	-2.39651482012406	4.12203522854958	2.20400052636117
H	-0.26771892802362	2.96025804541855	0.91095467580284
H	1.12464798591816	6.63957807418267	3.99937919062009
H	2.83080922886253	5.47097925714366	2.60091606971156
H	3.23947499345747	-3.85388476963345	3.53433156503809
C	0.00863309615338	-5.31086957912446	-0.53847712804068
H	1.70684424924070	-3.19306931373337	-0.46998047063900
H	4.91256835355690	1.87780684772873	-5.33670920000451
H	2.28558383558795	5.16730347811038	-4.33193968306484
H	2.03867297783623	1.45465071700967	-2.11221385296207
C	4.04923204779732	0.13357434280596	-3.40778089047589
H	-5.45920194063767	0.14137602211524	-4.06999092454242
H	-6.20504684603925	-2.23881067192528	-3.94944540548241
H	-4.11707371903025	-0.08407266894650	0.04757069852127
H	6.05313478077477	0.59633709580871	-0.97867434905307
H	7.53747303005825	2.02152128038475	-2.36244623863802
H	7.12511708932492	4.49441379255103	-2.54486908463957
H	5.17751285948269	5.52381328556855	-1.34921365372433
H	3.27044649573110	5.31108800192700	0.18099017650419
H	6.21757577437365	1.93983529615227	1.61895572818482
H	7.29493175259903	-2.73399598072547	3.06176774129299
H	5.11378574392311	-3.47966805648767	2.35666653635592
H	-3.30694601407642	5.60957290636336	-0.38483727320875
H	-2.88900780232991	7.81579966261180	0.66804218014398
H	-0.61070848852525	8.87357014561994	0.55486776580612

H	1.26096648319148	7.67843328642728	-0.60451902369226
H	2.06529298418395	5.78068464628987	-1.94514088301616
H	-2.43246087685351	6.07331919082053	-3.18972543689640
H	-4.12057261196906	7.42031615639743	-4.38442764371140
H	-6.45687242243806	6.54598182129737	-4.72096492121956
H	-7.03416262743912	4.23289301384289	-3.94458035539965
H	-6.35028516472459	2.09822340212459	-3.01064417279543
H	8.94407453386345	-0.88287202129904	3.44402851552343
H	8.35273883284800	1.46333016994898	2.74655918414865
C	-4.84037655697989	-2.65886351754766	0.51297372855071
H	1.69353376568809	-5.77912457564178	3.80614162975979
H	4.12951969150179	4.20972578025128	-5.72465557525203
H	-1.26842871765542	5.98253768337799	3.86699665646588
H	0.19579580660150	-6.46721228256895	1.92285033537470
H	-5.96208728411884	-3.52452501099844	-1.82551714126366
C	3.23912776053532	-0.85410324278868	-2.81298850804660
C	3.75340112654247	-2.11753009380868	-2.48825053896961
C	5.09937082119266	-2.45535377198734	-2.72831245372989
C	5.90322027380702	-1.47181645911830	-3.35033333160588
C	5.39316126441050	-0.21475210635074	-3.68717045992601
H	2.18566163080713	-0.64639199098383	-2.58558954003169
H	3.07465604360839	-2.84718344056634	-2.03114929123542
C	5.71041433355572	-3.80383619949563	-2.31607286520765
H	6.96284788455111	-1.68350944505342	-3.56115955234300
H	6.06776933827309	0.52992735062682	-4.13412237123788
C	0.45201940883448	-5.24396747504924	-1.87461254758427
C	-0.37854873289878	-5.62785746346827	-2.93568871439935
C	-1.69050869124316	-6.09131938182538	-2.71505950360305
C	-2.11920066660533	-6.18782524682249	-1.37204434554434
C	-1.29441044775101	-5.80650454316563	-0.30791613783743
H	1.46804091280432	-4.88331158616697	-2.09030400825615
H	0.01088710218205	-5.53826909910073	-3.95806168653556

C	-2.65830682057286	-6.42991604001954	-3.86059426005841
H	-3.13642543552564	-6.54510042128851	-1.14829663382536
H	-1.68283970269973	-5.85606941185038	0.72024371018360
C	-4.90330526148859	-2.05486754272680	1.78520733825871
C	-4.62529915616305	-2.78265554882477	2.95034474064520
C	-4.28160175848690	-4.14885698686868	2.90329432941295
C	-4.27620547613517	-4.76340518057608	1.63174104215254
C	-4.54071342097207	-4.03909761853593	0.46462559085107
H	-5.15863886627008	-0.98830107014682	1.86781375462704
H	-4.65844768861810	-2.25573508024077	3.91334673017502
C	-3.89168026932953	-4.95756567988476	4.15174638043398
H	-4.02512942591835	-5.83141450546013	1.53991992049634
H	-4.46307268080791	-4.54475486781297	-0.50880573557238
C	-2.83633963188662	2.89027180148706	1.68227239436424
C	-4.19714537936598	2.63336580923362	1.46532053119944
C	-5.18904937057224	3.58935198593542	1.75313768616356
C	-4.74821341826582	4.82020791608878	2.29324456718242
C	-3.39288956647586	5.07931558955160	2.51632568274910
H	-2.11300771430594	2.10823185682305	1.42307544983211
H	-4.48020004654852	1.65774553566776	1.05327888147163
C	-6.68084666063592	3.35253464256278	1.47154764793638
H	-5.47944071420343	5.60897524336464	2.52802376636983
H	-3.10236045058134	6.06835125120520	2.89878345213000
C	6.33812743590313	-4.48808862286634	-3.55133679103729
C	4.66318136470228	-4.75368001515125	-1.71006536981047
C	6.80710225019889	-3.54199717252773	-1.25629882361510
H	5.56896054128196	-4.68555641174665	-4.32574906579512
H	6.79314582066599	-5.45800176175664	-3.26230825518870
H	7.13247570862978	-3.86496093840893	-4.00801191394288
H	7.26417440123954	-4.49938560583062	-0.92991534877963
H	6.38073885580117	-3.04029179711341	-0.36387698850305
H	7.61567434156585	-2.89766970442545	-1.65623553201845

H	3.84664251268469	-4.97416501375551	-2.42716933141487
H	4.20635466946229	-4.33706867936723	-0.79067880490384
H	5.14325036415207	-5.71573066168144	-1.43975289625578
C	-3.77229505350453	-4.06602613477245	5.40048063355272
C	-2.52206083417357	-5.63626191470602	3.91156788422386
C	-4.96472927284796	-6.04006471998215	4.40879015499641
H	-2.19876326775940	-6.17878492074847	4.82422245529782
H	-1.75119390839990	-4.88327864982315	3.65520395097989
H	-2.56566326261764	-6.37210789678286	3.08388146379226
H	-5.07062822784689	-6.71629447393428	3.53670199549910
H	-5.95393185872029	-5.57841263794367	4.60603153818633
H	-4.69012364297651	-6.65830733262222	5.28902773068821
H	-4.73684265939465	-3.58519545915133	5.66084253952571
H	-3.01710029630407	-3.26687662621853	5.25796584888356
H	-3.45966556761336	-4.67970647968077	6.26948292276733
C	-3.86534594125257	-5.46391066179496	-3.78563798827439
C	-3.14986191444813	-7.88704953769111	-3.71397306981502
C	-1.99728101271707	-6.26976204695530	-5.24097284927409
H	-2.30152319073736	-8.59981698550521	-3.76926999196615
H	-3.86279537759452	-8.13591216157385	-4.52734215138149
H	-3.67011575381377	-8.04908798390192	-2.74880999995879
H	-4.57584131787759	-5.67020341645189	-4.61303627572114
H	-3.52669316273635	-4.41145521629994	-3.85778758053198
H	-4.41550075662868	-5.57542585122396	-2.82956314473840
H	-1.12164145351071	-6.94007013233052	-5.35820171855650
H	-1.66153315174190	-5.22783536069406	-5.41472806902142
H	-2.72687099228725	-6.52576376302211	-6.03566927275870
C	-7.13452189484210	4.34039911562699	0.37011555275508
C	-7.49719213619991	3.60162757338061	2.75959446620531
C	-6.95951043371525	1.91899187882617	0.98620606403739
H	-6.44616187479619	1.69648110718255	0.02978482509368
H	-8.04749604076273	1.78826976615314	0.81826279225036

H	-6.63932897192656	1.16074587196336	1.73007191445583
H	-6.98160106552597	5.39384636819357	0.67974331941900
H	-8.21321471785980	4.20202619931083	0.14827340910694
H	-6.56559172627299	4.17250794421493	-0.56691777512018
H	-7.18403410965346	2.90666943975777	3.56542973967788
H	-8.57766339442232	3.43995167638528	2.56570680100769
H	-7.37367310110764	4.63743912029585	3.13312779429356
H	0.13418611534057	-4.26856263279901	5.76452763281458
H	-0.16702342221420	-2.52406699015900	6.14097133955025
C	0.38172286165081	-3.23002274774907	5.48467426286086
H	0.10303975088835	-3.05197149947959	4.42615717934434
O	1.79923408997365	-3.10032938623169	5.67592069168138
C	2.40366133193886	-1.94477468360164	5.37183090276251
H	0.64013418614347	-0.80498545554003	4.75781939286389
C	1.72541747074563	-0.79270149940952	4.89555658567191
H	4.32265603710707	-2.81796155913651	5.90665562238049
C	3.81761444693428	-1.91377101716001	5.53722812366676
C	2.44502072856777	0.35334820030136	4.58641912019207
C	4.52238385252292	-0.75977900206774	5.23433436217432
H	1.89680931273732	1.22360226255701	4.20105942927752
H	5.61569228512123	-0.74003006097045	5.35716371746699
C	3.86242053495274	0.40872643019025	4.74676250516452
C	4.65444758336872	1.57743915781163	4.43631775548569
H	5.73705399887409	1.48089491079262	4.62263172756794
C	4.19897106446275	2.76521181233687	3.92460513854154
H	3.11791984064214	2.89917273483061	3.74753570000370
H	4.74306227455659	4.83413757615890	4.12093036054150
C	5.07179033879038	3.91689125747583	3.58453536515600
H	6.13794245952142	3.72325845896371	3.81016860736917
H	4.97533165254388	4.16635301190200	2.50199581042233

[X^{-}–pdt$^{\cdot +}$]

N	-0.37542553191443	-0.90300498281729	-1.40746582659224

S	0.82927546530163	-1.92106345193349	-1.11169691296851
O	0.43668517440679	-3.32776001429590	-1.36483118443709
P	-1.05551179110667	0.11253931159242	-0.37440750604431
N	-0.33746409671011	0.77544634959078	0.87222249122465
P	0.75591408112463	0.73041455129332	2.02039670671387
N	0.95845363359987	-0.46532560990644	3.06545506012759
S	0.22232429812392	-1.89137864999430	3.03773744621846
C	-0.68620710746621	-1.83641784566751	4.71416941186425
F	0.13106667888692	-2.25843004987936	5.69106255795794
O	-1.58040959884983	1.33030279655378	-1.38308115618380
C	-2.37946547941781	2.32217797568424	-0.83352505809699
C	-3.67881265001255	1.99625915888300	-0.40488425146561
C	-4.43859182432861	2.98932504279185	0.31420356927805
C	-3.93301584442978	4.33442070301291	0.38540171395232
C	-2.72089811244877	4.64946275322344	-0.28200091239951
C	-1.91724447781233	3.67181423416395	-0.87935872060601
C	-5.65816485200372	2.68445248166431	0.97772146185284
C	-6.35029969344350	3.66694978414111	1.67912615049902
C	-5.85888797368147	4.99558958721219	1.73917734710366
C	-4.66815995571261	5.32305055334593	1.10233338995697
C	-4.29114292672709	0.72150645490904	-0.84628169879966
C	-3.64080299490034	-0.50515086304390	-0.63635205106883
C	-4.15361403486684	-1.74783372927668	-1.13893667598061
C	-5.40361157395670	-1.69856343620904	-1.76730918679618
C	-6.10446282053337	-0.49205815835910	-2.01568553189748
C	-5.51821884637732	0.75748416535920	-1.61112128936517
C	-7.35094538808948	-0.49909053211638	-2.71128710805055
C	-7.98304396725244	0.68984647249169	-3.04445617465341
C	-7.36760638085014	1.92629736751781	-2.71753391403155
C	-6.16257492240504	1.96054618013259	-2.02494825401978
C	-3.45164030131456	-3.06084432034119	-1.09611883657591
C	-2.66441733780090	-3.50505685802324	-0.01370849927769

C	-2.06776615483900	-4.78625086260640	-0.01630740441328
C	-2.24865642472526	-5.62526706158251	-1.13843515894477
C	-3.02003399974887	-5.19532195197676	-2.22476667530408
C	-3.61653517741475	-3.92882810052588	-2.20681941683197
O	-2.49974445626103	-0.50000834790350	0.14879750118001
C	-0.72636289317054	4.09804399571688	-1.65695543773170
C	0.55596301714333	3.53774421248380	-1.49141338371361
C	1.66473374698371	4.03206871579449	-2.21461875722740
C	1.45523898779846	5.08134455601944	-3.13964468110224
C	0.18303617334787	5.63032884446901	-3.32702147935076
C	-0.90313143694475	5.15234711572692	-2.58470551547349
O	0.50984710531333	2.04203731575484	3.01515572951509
C	0.55366785904559	3.31239074946756	2.46270063034011
C	-0.63142406602989	4.10805941550583	2.51790860105750
C	-0.58086654987829	5.38235529962262	1.94352939459989
C	0.57655357847036	5.86991139336251	1.28157637897091
C	1.78760604649691	5.09201869454108	1.31978447348569
C	1.78303232018806	3.82572766423859	2.01012257185887
C	2.93684419681456	5.59151386104448	0.64771693280955
C	2.89570932539176	6.80947057806177	-0.02340217396436
C	1.70755399111559	7.58289609171324	-0.04873484563901
C	0.56623106013200	7.11823664913964	0.59272204928647
C	-1.84259784592765	3.69108542636024	3.27184141997539
C	-2.37676889361817	4.60285168553967	4.21359472150941
C	-3.52114288482754	4.26913380260183	4.94834029202647
C	-4.15984878050321	3.04082370684751	4.73735838335044
C	-3.66275628290262	2.11782767932346	3.78705552382900
C	-2.48930240881350	2.45349824446563	3.07410910725677
C	3.03871054765774	3.12462787109584	2.37495099566807
C	3.18922478535364	1.75458313437930	2.11502856254526
C	4.30345638792876	0.99401135439530	2.58823214343916
C	5.32725140024090	1.69202185222903	3.22857991713858

C	5.25309762617196	3.08427500924150	3.49658788233319
C	4.06548589776177	3.81261297700578	3.12765681533089
C	3.94946097500256	5.16292491554300	3.57430595470752
C	4.98421793955865	5.78290042445282	4.26407979539098
C	6.18553040028015	5.08430684039112	4.55366364464238
C	6.30832807232029	3.75259561152386	4.18719870391546
O	2.21460451188833	1.12448728680903	1.35073879711320
C	4.38078297213185	-0.49211892790200	2.54302881512323
C	4.58407841871330	-1.17064844711881	3.76815434500473
C	4.68402452206663	-2.56692343548677	3.79840177888086
C	4.60196700191764	-3.30298884940391	2.60866015771659
C	4.41618264787800	-2.64815475236208	1.37254830298977
C	4.29159190390105	-1.24307181375368	1.35728456190039
O	1.19789042136408	-2.99087155821985	3.18331475839037
O	-0.86071715121658	-2.00722473951565	2.02571646035514
O	1.59853098739816	-1.61778351400514	0.12155537117935
C	2.00553954571956	-1.50850973694019	-2.57721528425843
F	3.12945198697379	-0.95106196458078	-2.10507259824808
F	2.31757079750113	-2.63912716510123	-3.22453118418846
F	1.43691177082649	-0.65952522965833	-3.44560575224250
F	-1.76415105828898	-2.63597444271487	4.67291012566098
F	-1.09048952936315	-0.58799640463879	4.99270579285907
C	3.02785254019882	3.50038463020697	-1.97616958317289
H	0.70052184740702	2.74648316883536	-0.74350545651800
H	0.03270581774534	6.42956579661208	-4.06781675672444
H	-1.90655824199174	5.57768253511144	-2.73524309845035
H	-4.19189906546406	-3.59247401240952	-3.08104061187679
C	-1.26680006867298	-5.25828212591001	1.13815186722369
H	-2.47648107919256	-2.84240360774312	0.83807845989290
H	-5.05123352306270	2.78627853385423	5.32930633035079
H	-1.87080396061416	5.56468151141891	4.38541287226138
H	-2.10240492861985	1.76443712509415	2.31124754861665

C	-4.38575195892507	0.85035178793638	3.51648617913479
H	4.63670649420089	-0.59183127449680	4.70255061654306
H	4.82485744643631	-3.08724868931794	4.75810395969958
H	4.09463588538274	-0.74105435146297	0.40357292835965
H	-6.03754684156939	1.65393772229288	0.95817906755288
H	-7.28275588690537	3.40490694721024	2.20119225092978
H	-6.41581971413382	5.76164752127326	2.29910636602136
H	-4.26763364563391	6.34750584471898	1.14795399117328
H	-2.39166525402181	5.69773665544894	-0.31644842920238
H	-5.69868052945235	2.93007675448748	-1.80474181485511
H	-7.78815793627697	-1.46941494742313	-2.99307954315849
H	-5.86555445752910	-2.63735314094305	-2.10521289118173
H	3.85794700515674	4.99327466573189	0.63908765754695
H	3.79279360708190	7.16797446277725	-0.55006725415705
H	1.68865706658210	8.54319148809581	-0.58545437221418
H	-0.36838372678665	7.69982069735237	0.57249916680581
H	-1.47400637262481	6.02225971346173	1.98620757226204
H	3.02606020188631	5.72246172322137	3.37815276402434
H	4.86561843114167	6.82612530255660	4.59413116559925
H	7.00228550284318	5.59154127013698	5.08894291704203
H	7.21386273758346	3.17926250306828	4.43922794606312
H	6.21506889723598	1.13771591398012	3.56895630806862
H	-8.94202368382589	0.67756809151948	-3.58367720545920
H	-7.84235692485258	2.87068326831333	-3.02367929533552
C	4.37889512251151	-3.43448514139536	0.11514679824306
H	-3.15840318150970	-5.85064684755614	-3.09835886243215
H	-3.91630447761442	4.97212529746648	5.69737919480965
H	2.29645842500976	5.45301499152554	-3.74087898372973
H	-1.79365074592698	-6.62711362608486	-1.14696095373995
H	4.69555333423574	-4.39906651361672	2.63070401890384
C	-3.72094477390209	-0.29874054548847	3.04394945383966
C	-4.42436284720010	-1.47572516899322	2.74817284279722

C	-5.82317946907054	-1.56013844578795	2.89159945671995
C	-6.48564779015044	-0.40885224686271	3.37834026366759
C	-5.78819157093467	0.76144379582735	3.69013161787475
H	-2.63390556734881	-0.28981730103416	2.89381659487274
H	-3.85156796947327	-2.34134864596525	2.39404059381900
C	-6.63277383548307	-2.81284568363629	2.51921312678186
H	-7.57932919147608	-0.41857581479186	3.50346424370501
H	-6.35135882870310	1.64120761427882	4.03427004878007
C	-0.06114403495344	-5.97207045384693	0.94721330162210
C	0.69464936187096	-6.41189379657325	2.03946704921151
C	0.28484977524107	-6.16968413128503	3.37109714849499
C	-0.92877840867260	-5.47787971401926	3.55288310832867
C	-1.68528648350105	-5.02622652883508	2.46384052339700
H	0.30893262187995	-6.14813597834017	-0.07441074753878
H	1.64089663212839	-6.94056781419498	1.84564840070380
C	1.16805646302625	-6.62203691819150	4.54503671774898
H	-1.29553795080155	-5.26332894660095	4.56483217750845
H	-2.62600710483154	-4.48769708877582	2.64729959432425
C	5.16840696869012	-3.06518039259176	-0.99568541948073
C	5.19999449844533	-3.85462768651277	-2.14984166247726
C	4.44258460412538	-5.04307589162154	-2.25668659258775
C	3.62667629555839	-5.38536660062134	-1.15822381320672
C	3.59623841966332	-4.60115505145735	0.00563608539693
H	5.78744077059157	-2.15670843248119	-0.93961411794698
H	5.84259322910661	-3.53881976742016	-2.98618370793287
C	4.55289157220911	-5.90858526064576	-3.52406094698280
H	2.99364689640875	-6.28309457580110	-1.19728804642186
H	2.93243881526905	-4.88588164251682	0.83632987447361
C	3.24402087640804	2.19005381819654	-1.50357513994797
C	4.53375165186761	1.73921840213889	-1.18564805783812
C	5.66574346731781	2.56601351193874	-1.32024768152621
C	5.44981055150013	3.86443901794698	-1.83716916259106

C	4.16867044785660	4.31851412927174	-2.16117513398615
H	2.39465218901613	1.50638040870500	-1.36615004993348
H	4.64865244207297	0.71299323778269	-0.81890821442514
C	7.07708625260720	2.12794215013367	-0.89853121967574
H	6.30092996609679	4.55030771443964	-1.96738975517672
H	4.04654413193710	5.35214545487282	-2.51601260754063
C	-7.46328344270170	-3.27354654809994	3.73844944390903
C	-5.73088224790985	-3.97665493337497	2.07533933907847
C	-7.58389984744521	-2.45358153201741	1.35284614050000
H	-8.06365732067881	-4.16941051916450	3.47689877444982
H	-8.16389012437189	-2.48650915150831	4.08121505976106
H	-6.80249765412654	-3.53845874150936	4.58909557866490
H	-8.28561698505952	-1.64329247563570	1.63503516013180
H	-8.18499059061413	-3.33891442753227	1.05826309850950
H	-7.00956978980597	-2.11576031796364	0.46631058160854
H	-5.02643068222452	-4.27758942924540	2.87722436914464
H	-5.13542605137644	-3.72305552828867	1.17617080037382
H	-6.35439430139029	-4.85895478490828	1.82668562690515
C	4.14874634074016	-5.07493644544224	-4.76207915626927
C	3.65965608566471	-7.15866925754944	-3.45046684535356
C	6.02033224312464	-6.37169437924736	-3.68425800340056
H	2.59171052158238	-6.89454154932166	-3.32248577062949
H	3.95027354227120	-7.82098671587804	-2.61005664783701
H	3.75431060937385	-7.74184990192637	-4.38850617042358
H	6.34378557567560	-6.97193791271838	-2.80934912137177
H	6.71135681758541	-5.51104686365141	-3.78280262096386
H	6.12772951168691	-6.99962760897397	-4.59312953369442
H	4.79209571097674	-4.17972435591406	-4.87501388016393
H	3.09876578890603	-4.72940682820885	-4.69703572379317
H	4.25448058547115	-5.68464912109984	-5.68341777499872
C	1.33993993177899	-8.15692690890547	4.49407844358160
C	0.56256224929630	-6.23668617321977	5.90629704746969

C	2.55048326201774	-5.93924472958833	4.42230737041070
H	-0.42927841414364	-6.70599548158001	6.06718665883122
H	0.44536621914996	-5.13867793143935	6.00528444628627
H	1.23211423938937	-6.57840408731750	6.72130435878089
H	0.36002442650130	-8.66882296421752	4.58567728530096
H	1.98823154365409	-8.50051432578597	5.32688669167218
H	1.80850554789426	-8.48282533561468	3.54389395361528
H	3.06888054496680	-6.22789741740193	3.48602302024083
H	3.20005510468489	-6.23232376523225	5.27331639790571
H	2.43964342872398	-4.83725486482542	4.42068595453981
C	8.04608927781132	2.27268366793231	-2.09306307855537
C	7.11027318950670	0.66826842781501	-0.41389377344679
C	7.54288161912269	3.03865860779949	0.26270290984344
H	6.85392874313396	2.95453012947256	1.12729261999551
H	7.58026859781505	4.10369144014406	-0.04221175294514
H	8.55835646712945	2.74430964922776	0.60063900392632
H	7.72877337001418	1.62914549950372	-2.93897440297400
H	9.07048007232461	1.96858982393685	-1.79408978829355
H	8.09812668442685	3.31758720877308	-2.45863174001077
H	6.46137635897380	0.51245195164027	0.47044678975317
H	8.14443822592457	0.39459581056774	-0.12371404826612
H	6.78497411304742	-0.03503327141388	-1.20736972647037
H	-5.63306700323330	1.16957842646345	-4.79017765686349
C	-4.77946871010359	0.81026724644324	-5.39952074026744
H	-3.14692538989855	2.95724572691446	-3.47659794816139
H	-4.94285444263711	-0.26871889973977	-5.60213879963725
H	-3.46403935805436	0.50127430298227	-3.70509405377666
H	-4.79729149239185	1.34739828386800	-6.37100509090383
C	-3.46076154139272	1.03237640944020	-4.67956208712551
C	-2.92259612943337	2.46905860286572	-4.44528218811235
H	-3.23467479773228	3.14326864247381	-5.26912238359291
C	-1.48650335398710	1.93249672357794	-4.64764855525134

H -1.70085786692164 4.05064948449293 -6.43400937999702

H -0.27576269751003 -0.26556373159104 -7.15239830118037

H -1.10724539018926 1.53067473313839 -3.68884624917513

C -2.14664307683621 0.71035308935673 -5.43378830925072

H -2.24288091094054 1.03182813365436 -6.49323254843479

C -0.49898005554727 -0.97181375597305 -6.33755305573145

C -0.66068596894240 3.74807643382212 -6.23852605502124

C -1.47708664723404 -0.61933775714588 -5.36808930795307

C -0.39713584008007 2.69797453247212 -5.33185709960145

H 0.92433314827463 -2.46945661887082 -7.03211196607483

C 0.17275529822717 -2.18867081077888 -6.27982195497817

H 0.16079192127381 5.24382105409229 -7.58130003012314

C 0.38693920138417 4.42066142109705 -6.88569154593459

C -1.73651090435160 -1.53940705746635 -4.32642671064955

C 0.94915398237403 2.33497514586807 -5.10254687999166

H -2.46757559698018 -1.28880197515456 -3.54496873431177

C -0.12067099234854 -3.10569924029647 -5.23585094326066

C -1.07748593002159 -2.76616921778743 -4.25112682131422

H 1.17238545520112 1.52801371400602 -4.38932354098249

C 1.72103289897041 4.04839894521520 -6.64650212333106

O 0.55038411195204 -4.27584160688473 -5.25832097162673

C 1.99785443783168 2.99873231491925 -5.75284018934712

H -1.28692431168735 -3.43975960185606 -3.41323759527076

H 2.54336952075267 4.57660309067249 -7.15334984821466

H 0.51726250635680 -4.85761409954593 -3.24182566029511

C 0.26117177766919 -5.24864332078277 -4.24831754797560

H 3.03967025344432 2.70692280309697 -5.54810696565358

H 0.88578294839944 -6.12691515578224 -4.48577359401322

H -0.81068516533546 -5.53501550892709 -4.27299711585544

[Pc$^{\cdot}$–X^{-}–sub$^{\cdot+}$]*

N -2.60526287482010 0.69594430961085 1.76442439361601

S -2.99626662521632 2.10503227692820 1.11348898207755

O	-4.46109898590115	2.18009973299299	0.92421373299821
P	-1.30752392702330	-0.20245768809091	1.46508112687774
N	-0.12449270963104	0.20747712118896	0.50038701310020
P	0.59804051756489	0.71762124338004	-0.81634533255622
N	0.13614886043284	0.36349247011801	-2.31305713567242
S	-1.34624515604606	-0.04376412195714	-2.77598471375461
C	-0.92191046527839	-1.38643852541273	-4.06923775244392
F	-1.91393058727815	-2.28690744844668	-4.12957496319370
O	-0.71469057528141	-0.46410874332071	2.99524486856029
C	0.16581811860816	-1.49951743486788	3.26162021586977
C	-0.26956988901698	-2.83257131660247	3.13750568947733
C	0.69662202273490	-3.88735668640384	3.32727380742940
C	1.99873286914308	-3.55727290780773	3.84358314007846
C	2.31589375700047	-2.20024312712308	4.11116713439737
C	1.43621688835365	-1.15352838062841	3.82703870059335
C	0.41352180091580	-5.25170098375540	3.02985207999792
C	1.34856536872929	-6.24651141524114	3.28441237085310
C	2.61245420500782	-5.92517374570490	3.84510859907578
C	2.93502322862384	-4.60088650728498	4.10534279748357
C	-1.71940977636315	-3.13313732275166	2.98864484887881
C	-2.49497819518259	-2.49420435540829	2.00853166343509
C	-3.91950509121950	-2.64699185058553	1.92127909300566
C	-4.50561117483142	-3.58585425350485	2.77191292976790
C	-3.77879532274423	-4.28422926394541	3.76681928587010
C	-2.37507928010465	-4.01170464088410	3.93606315449368
C	-4.43889922764822	-5.20079074947903	4.64044718953509
C	-3.75376824824572	-5.81049251052979	5.68018608096050
C	-2.38997380469773	-5.48836814439803	5.89755969545979
C	-1.72387018087911	-4.60517315373113	5.05636764069298
C	-4.83381037220389	-1.82067597686170	1.08434940553385
C	-4.62339137561546	-1.53448683239284	-0.27841331377752
C	-5.54525184675975	-0.75323390781720	-1.01236829725955

C	-6.68952252641797	-0.24853108502435	-0.35423443176727
C	-6.91431905219436	-0.53370030784099	0.99804176056661
C	-5.99917429260499	-1.31585067940982	1.71224974819664
O	-1.82887871906997	-1.71351971241635	1.06754181115305
C	1.75921965630357	0.22377154624324	4.29421699086708
C	1.52713464998510	1.39299461927524	3.53892911171785
C	1.84168473743498	2.67796765405417	4.04310520766352
C	2.36747398800684	2.76874199285605	5.35445969657988
C	2.61835633277118	1.61892082778987	6.11030092784293
C	2.33047750226777	0.35406851076173	5.58446590132471
O	2.16792282661773	0.18610731764222	-0.83445859931718
C	3.03261034927889	0.58260575638021	0.18216790945901
C	3.54720847238815	-0.41366269841679	1.07890511849240
C	4.39412627758751	0.03659247558013	2.09430465536750
C	4.74219989718851	1.39897032503405	2.26192947911455
C	4.26084243718614	2.37131588513170	1.31629855278766
C	3.44531155554929	1.92249347118775	0.21484883209891
C	4.59443208910051	3.73985901187802	1.52602683616482
C	5.40666977236570	4.12129966890922	2.58562164091230
C	5.91489750813009	3.15596062815315	3.49538294586430
C	5.57615710551129	1.81953871336660	3.34149066645731
C	3.34198219392254	-1.87497710135652	0.90414131328131
C	4.41312788749222	-2.74239822399462	1.24233435126209
C	4.29176581215221	-4.12472046265049	1.07628940336229
C	3.11295334882378	-4.67609938602334	0.56806507360251
C	2.01591229240218	-3.84615336843837	0.22888472776326
C	2.15477702405702	-2.45145350477686	0.41142196576067
C	3.12284525890464	2.83639075088248	-0.91568087008035
C	1.80235556735377	3.01364037917477	-1.34993872672870
C	1.44653139694623	3.89167803979009	-2.42773663202183
C	2.47935386730576	4.62912593599312	-3.00726571312740
C	3.83883522822817	4.47044350571137	-2.63884846635488

C	4.18540127539398	3.52107997047376	-1.61499076539500
C	5.57148093199280	3.28261704927896	-1.37080879877730
C	6.55548073865967	3.97284604583777	-2.06560436557044
C	6.20563151455728	4.95272832385312	-3.03333043946179
C	4.86937205535990	5.19251544386856	-3.31298043984543
O	0.79433265230854	2.34859506056454	-0.65857759303161
C	0.07651817492941	4.06822931452922	-2.98667812603639
C	-0.06268368185001	4.13660439545886	-4.39432777730831
C	-1.30941263067822	4.40432049043314	-4.97337205418032
C	-2.43397749990956	4.61596905865838	-4.16614773668663
C	-2.32917762442622	4.54772503494304	-2.75877677090791
C	-1.06844608935974	4.25967761584947	-2.18795115808168
O	-2.00229511130782	1.02034318662167	-3.56360494189971
O	-2.12778369351556	-0.75040001278706	-1.73027326401765
O	-2.09706568163995	2.54054488737164	0.01742245331089
C	-2.64680609969805	3.29900053251138	2.57415847772012
F	-3.76512151114772	3.47803312115123	3.29860571751269
F	-1.69502094658357	2.79493451604568	3.37931530443475
F	-2.23774090496571	4.48876123363355	2.11574406717722
F	0.21123882619337	-2.03165947797366	-3.74339949655857
F	-0.77236228881410	-0.83218622968090	-5.28440549919729
C	1.68438053258116	3.89820070878137	3.20812313315965
H	1.14107166060220	1.29163502988239	2.51703957618142
H	3.03423551232720	1.70812147017396	7.12540295239184
H	2.52659696077522	-0.54262784144013	6.18923452949984
H	-6.16497535002103	-1.51325622301853	2.78094188502071
C	-5.32618667042148	-0.45359016888789	-2.44935569841113
H	-3.71259488875091	-1.88780435765959	-0.77370793864410
H	3.04647717069130	-5.76148709154532	0.42422403610540
H	5.35963410671065	-2.32507836644538	1.60908594468840
H	1.29716249877957	-1.80524777634835	0.21135052977824
C	0.75338376575978	-4.42091387056720	-0.28886373347425

H	0.81373939218228	3.97841490355582	-5.03766468904639
H	-1.40441279272829	4.45418410032437	-6.06888145335024
H	-0.99789610935969	4.15994073133962	-1.09921775754920
H	-0.55662744978598	-5.51337271839505	2.58706241277709
H	1.10869983001056	-7.29260579939994	3.04051876824634
H	3.34149100548705	-6.72382601187699	4.04907471263426
H	3.92381116793154	-4.32983933327310	4.50619500812933
H	3.29780737509102	-1.96926328315503	4.55005342434752
H	-0.68201890859932	-4.34865592482096	5.27596196449459
H	-5.50946206255758	-5.39899668557818	4.47633886510182
H	-5.58744072540694	-3.76942990492687	2.69341873028315
H	4.17802275381099	4.50299754400197	0.85511557492480
H	5.63892800458519	5.18668134516159	2.73338842177429
H	6.55840771180837	3.47315132785619	4.32992221259271
H	5.93772646612742	1.05935415538163	4.05153168252325
H	4.79763296863489	-0.68720741705299	2.81592333100715
H	5.86582474313557	2.53684972112163	-0.62242474889239
H	7.61548274171147	3.75664458844792	-1.86331002465250
H	6.99376468814090	5.50458058264038	-3.56744828381154
H	4.57747193267999	5.92684581795511	-4.07984417928873
H	2.23548912728986	5.35015655808108	-3.80111235057514
H	-4.26893916908555	-6.51559023138203	6.34979430608155
H	-1.85301294294322	-5.92342067001385	6.75419570078297
C	-3.52141927408252	4.77546589062494	-1.90481837895154
H	-7.80779373692078	-0.13748443921281	1.50476260552319
H	5.13995618340453	-4.77886094607874	1.32715071896250
H	2.58453136918421	3.75255617314400	5.79322248820838
H	-7.41458596564387	0.35920538946156	-0.91678033888217
H	-3.40248929088630	4.85455059168509	-4.63063561884731
C	-0.23067666632338	-3.60999802909094	-0.90097701898228
C	-1.43227354314710	-4.14828119557969	-1.36263824037137
C	-1.72789617573941	-5.52654302829766	-1.24412944332196

C	-0.74456203915269	-6.33921077225568	-0.64400780679678
C	0.46530035049947	-5.80220508357674	-0.18202627057042
H	-0.06591725775157	-2.53361106015224	-1.02933478917779
H	-2.15532701331834	-3.46457268433296	-1.82844313881168
C	-3.06235518002315	-6.06932950998694	-1.77612358970193
H	-0.91458713278134	-7.41762574496322	-0.52167837062058
H	1.18875924459152	-6.47860496884510	0.29201007414562
C	-4.85650108685180	-1.43182679177198	-3.34713655217254
C	-4.63182743065491	-1.12898658375201	-4.69801363068382
C	-4.85033811815227	0.16456459131674	-5.21086942715007
C	-5.35282561259638	1.13266676826945	-4.31299343445554
C	-5.58718017215464	0.83591503010031	-2.96632701849448
H	-4.66353342824380	-2.45256686764342	-2.98337776185078
H	-4.25627955494480	-1.92550048102014	-5.35342753751381
C	-4.51417925799350	0.55616996067672	-6.65982997905436
H	-5.53983717068634	2.15823381469971	-4.66801435522761
H	-5.92988791917758	1.63120666741004	-2.28702262986649
C	-3.42433372895019	5.39678895433103	-0.63936143841247
C	-4.55409020371581	5.57969342529581	0.16388449890724
C	-5.83677077064445	5.14827013691323	-0.24374056552631
C	-5.93328823865620	4.54690988627004	-1.51432346350468
C	-4.80397194942280	4.36954295083379	-2.32742002611413
H	-2.44505814949812	5.74178370243268	-0.27669328062767
H	-4.42615619847355	6.05815103439879	1.14677381480608
C	-7.04364732229896	5.32078583578326	0.69321091851641
H	-6.90221927505183	4.18411356408787	-1.88419499460779
H	-4.91867918428952	3.86634718779624	-3.29821423453694
C	0.92164792777236	3.89513822577502	2.02362095786085
C	0.85995457133723	5.01930534316266	1.19036610197572
C	1.55410181588408	6.20696663734028	1.48981161365918
C	2.28375459569797	6.22554766355752	2.70004100613602
C	2.34824719290327	5.10586598969449	3.53434793608019

H	0.36666291774792	2.99985124292918	1.71961088848754
H	0.25629221106218	4.95051086375436	0.27826813359885
C	1.59283962738780	7.40949761852917	0.53427508111385
H	2.84835502214609	7.12574004499875	2.98749504202342
H	2.97469477939567	5.16538774221570	4.43534060254177
C	-3.24285377255405	-7.56687499918904	-1.47588472175428
C	-3.09964764645909	-5.86059985669762	-3.30894108547218
C	-4.22961150506198	-5.29490583544851	-1.12241976236165
H	-2.45576797728851	-8.18368091140794	-1.95452107356222
H	-4.22128346058996	-7.91206756242368	-1.86640178542599
H	-3.22315358152353	-7.77018160627992	-0.38588206442245
H	-5.20062139155512	-5.67212325324209	-1.50419576718717
H	-4.17909063368733	-4.21096745388626	-1.34182386344934
H	-4.21962644861988	-5.41666245732678	-0.01980011656491
H	-2.28275212462462	-6.42645918749108	-3.80305617500259
H	-2.98237733428400	-4.79210872003362	-3.57854138300203
H	-4.06575448247070	-6.21582613509997	-3.72379094391534
C	-6.75846483029271	4.58995292229164	2.02573232310484
C	-8.33343043492598	4.74087167192861	0.08736301503869
C	-7.26142869612880	6.82605545846993	0.96923058727509
H	-8.23866491295395	3.65432916762821	-0.11453117022719
H	-8.60694527683961	5.24571728649371	-0.86123623970828
H	-9.17422200217523	4.87978886699046	0.79669901437888
H	-7.47170419914943	7.37566893245341	0.02885858302698
H	-6.37057280837486	7.28558572909163	1.44220883627856
H	-8.12269826950153	6.97172006280258	1.65408572373013
H	-5.87127929643956	5.00915934145123	2.53920079956056
H	-6.56018397097223	3.51411889026033	1.85011271909455
H	-7.62678490869648	4.68726880321955	2.71022032311827
C	-3.42850326232835	1.65949033032711	-6.63497644532712
C	-5.78182757145988	1.09484286924704	-7.35955724034845
C	-3.97327947015639	-0.63464453461424	-7.47053228994233

H	-5.54683998615915	1.40161926791410	-8.39999693929528
H	-6.19546832176601	1.97778268284537	-6.83268205268003
H	-6.57360697088343	0.31876947104622	-7.39951091973495
H	-3.15336870051615	1.95233945638041	-7.66991169945984
H	-2.51993955935286	1.30584654903556	-6.10912611755031
H	-3.78163384368197	2.56713605920064	-6.10640306598093
H	-4.71032192412194	-1.46092429928133	-7.53490398576109
H	-3.03899508373542	-1.03897268233372	-7.03157113473893
H	-3.74404831646206	-0.30789418999075	-8.50507226540708
C	3.02280970051767	7.50534432209036	-0.05191378412720
C	1.25839097351788	8.70898086514327	1.29850174061753
C	0.59842595773256	7.25541960778151	-0.62959457220100
H	0.83645102146024	6.37778236687717	-1.26147208296031
H	0.63813331707977	8.15315000736134	-1.27892984706034
H	-0.44456550864587	7.14427370582633	-0.26913258851788
H	3.77977381436269	7.63913704989990	0.74719137876150
H	3.10021677061984	8.36833326823329	-0.74592697215458
H	3.27902714474694	6.58562833811601	-0.61681141015725
H	0.23947652056367	8.66030597758842	1.73430880778699
H	1.29650995412878	9.57718266911015	0.60886815060440
H	1.97506447157464	8.90268197535702	2.12077231478755
H	-6.13292708796089	0.99433709010734	3.83253273640520
H	-4.70764973801198	1.60459514954548	4.76891714565233
C	-5.11002289111580	0.75961137771211	4.17442276771175
H	-4.45778462194831	0.57925245145546	3.29676121158883
O	-5.22056820787455	-0.41636794157099	4.98785395857367
C	-4.11462592254405	-0.89848834587857	5.58415970760319
H	-2.63040846967959	0.43978579006984	4.67296203076964
C	-2.80841436277903	-0.38540533705567	5.37327279709868
H	-5.33029206118181	-2.35613150741669	6.63969462723135
C	-4.31010451033818	-1.97443311830928	6.49054898431280
C	-1.73199076487790	-0.95094741707268	6.05085893561660

C	-3.22664407552084	-2.51433423866116	7.16905414122728
H	-0.73021655571015	-0.53200529719004	5.88138663446870
H	-3.38415684297651	-3.35026507210487	7.86669845745515
C	-1.90187224911195	-2.03361622938181	6.95720291326377
C	-0.78983672310279	-2.69163471944274	7.62697202113971
H	-1.06622663550665	-3.42107940066314	8.40933433630935
C	0.52956230861762	-2.54060795038163	7.31933162263868
H	0.80310769987968	-1.85203133563385	6.50208345487964
H	2.19295690482946	-3.89546454227287	7.21310763937470
C	1.65498557137166	-3.27294408838626	7.96382050486142
H	2.41605678401744	-2.56516015258468	8.36042608240586
H	1.31489361420253	-3.93040032850114	8.78770810772468
C	2.51955787174814	2.38743401640810	-6.30324550354241
O	2.14630191958257	3.45392383088623	-7.06782212845813
C	1.04830813983605	3.28635514279111	-7.95877347843293
H	0.91827665743052	4.25320948290447	-8.47820489316523
H	1.25164229532142	2.48951115937903	-8.70682412264967
H	0.11192319094492	3.03558359671222	-7.41430167979185
C	1.84579770055588	1.14878259149807	-6.27430128129323
H	0.95511842056235	0.97123552300049	-6.89145752446629
C	2.30758305464432	0.12808327535035	-5.43781007010870
H	1.75105738368243	-0.81655301490301	-5.44153092246761
C	3.43531895756669	0.28688572334712	-4.58882465495046
C	4.09124729488176	1.55084728431514	-4.63602722194665
H	4.97383231432067	1.74143139126705	-4.00898988820729
C	3.89918467378756	-0.79939037697533	-3.72839869647022
C	3.36333344504282	-2.11868644571631	-3.81938464694845
H	2.55375351866025	-2.33969246461999	-4.52137886592458
C	3.84026068922277	-3.16909534478371	-3.05970042321149
C	3.30641634003112	-4.52272405480272	-3.04251580860656
C	2.06270472104439	-4.83204051970169	-3.65553731602845
H	1.48245753049196	-4.04571968939514	-4.15537656747187

C	1.51574238471701	-6.11211852794166	-3.59843622131531
H	0.53662059631797	-6.29594333631451	-4.05964058971187
C	2.20095394554391	-7.14166724108687	-2.90824412215194
O	1.73401354154613	-8.40497446049011	-2.76395164203137
C	0.49687901758188	-8.74989739326197	-3.38329202545514
H	-0.33621613769082	-8.12250992087660	-3.00168631709220
H	0.55454822532700	-8.64292108661193	-4.48775933439677
H	0.30618060332181	-9.80735701998556	-3.12595134671741
C	3.45276932592019	-6.85921690128281	-2.31220888260984
H	3.97841891029485	-7.67000786025172	-1.78659659066978
C	3.99169245006201	-5.57672629728640	-2.37443623628321
H	4.95512189103007	-5.36854739729884	-1.89067262224465
O	4.91578687712652	-2.98215141512998	-2.21087989020324
C	5.40047188930208	-1.70211418949380	-2.00546006790364
C	6.42996055296442	-1.64218604994813	-0.97726541898697
C	6.72684558147512	-0.42928252843213	-0.30844765907458
H	6.16870165197029	0.48187008821861	-0.56087440193719
C	7.66885202654201	-0.36828486473516	0.71973846307682
H	7.83660596534368	0.58781398772788	1.23278964808458
C	8.35658205796287	-1.54125203160657	1.11279929327603
C	8.08723282773474	-2.75923905743770	0.44151586113680
H	8.63450813050506	-3.66130301010884	0.75265132937755
O	9.27090174007007	-1.59299114665390	2.11212333030301
C	9.54924735303830	-0.40119312196288	2.84191761841238
H	8.63683682035923	-0.00768575271704	3.34073718888314
H	9.96959746160565	0.39020425277313	2.18467083476358
H	10.29670076398867	-0.67469779606539	3.60836626218506
C	7.13908270467574	-2.80955581549003	-0.57680705234007
H	6.92689645362168	-3.76511353113062	-1.07555644372608
C	4.91859937210366	-0.63650854876002	-2.74107931881426
H	5.35641066093905	0.35057764522188	-2.54566827804161
C	3.64923020974978	2.57194029041670	-5.47478374826553

H	4.16908909746716	3.54003495172699	-5.50240060087007

TS → (1*S*,2*R*,4*S*)

C	1.09671998207614	1.60457670652175	5.59812115335609
C	1.73948766924705	0.88278943572646	7.92349034924086
C	1.51308286468705	0.07038259461260	9.03589041410674
C	0.41832956189219	-0.81504710292368	9.05462670576489
C	-0.46049672521965	-0.87269186947329	7.95559795135405
C	-0.24301623487509	-0.05810517142347	6.84303915597160
C	0.87152833574151	0.82841792180650	6.79304775019486
C	2.22370863955755	2.39203513326585	5.35035888025103
H	0.35840489971824	1.48841742413031	4.78600793840993
H	2.18572405294123	3.06295445246648	4.47943491999676
H	2.85424986386873	2.71983598115951	6.19060478014914
H	2.59182966517400	1.57584610669540	7.92082672960174
H	2.19082799781908	0.12293311306692	9.90072639031766
H	0.24240518505173	-1.45928815829587	9.92935755352034
H	-1.31914125756241	-1.56135633916337	7.96276632817357
H	-0.91527052823542	-0.10969390202651	5.97222419425680
N	0.42622282592975	-0.61207283299947	1.65836794355912
S	-0.92264851468323	-1.28862298050466	1.28207064695959
O	-0.96416282495556	-2.70613207851486	1.48600243763191
P	1.19673910666211	0.22702238560119	0.51422216056643
N	0.44922353310033	1.14058568730489	-0.55648721446274
P	-0.83922893964441	0.99939429203721	-1.48699828627972
N	-1.20509936311475	-0.31435518744694	-2.34576114790790
S	-0.77984487395626	-1.69010541730251	-1.75632175416472
C	0.06925022125153	-2.33207162470565	-3.37470769329764
F	1.16472144983653	-3.07496489478421	-3.15549393660381
O	1.96506683931389	1.29546552384795	1.47591705295639
C	2.77563357514331	2.19612138868869	0.78005941164108
C	3.94579604522581	1.74879722022619	0.19556078762038
C	4.68331984044941	2.61837633667076	-0.65695440185422

C	4.29106587673332	3.97754816781400	-0.75630931765067
C	3.22810799463649	4.43358786834435	0.04528921260171
C	2.45361293593581	3.57209851813166	0.78444283646617
C	5.77900550964319	2.17297924632972	-1.42552981448019
C	6.45524973266619	3.03666833448745	-2.24257802114979
C	6.06919319973195	4.38133405229905	-2.33801324481342
C	5.00946661292856	4.84197758757064	-1.60848691208118
C	4.46914485929806	0.42686714613438	0.58683843803104
C	3.65929555833196	-0.68969222837163	0.52231517803549
C	4.09557715553634	-1.94347761088994	1.02516632946075
C	5.39972944243283	-2.04296077198684	1.45798720359572
C	6.26402890551778	-0.93821269792618	1.52831960330992
C	5.77671862808180	0.33896513631256	1.15281977512765
C	7.58239124382099	-1.06865877364790	2.01812901076430
C	8.37950037554493	0.02890050655264	2.17973391733562
C	7.87381340417281	1.30385815172457	1.88605625246662
C	6.60581371212657	1.45795548505059	1.39277538569951
C	3.24296772596516	-3.13699554827536	1.19056093815049
C	2.39922011604834	-3.64118766975184	0.20896561314462
C	1.66116606563516	-4.80016878833048	0.43357833622466
C	1.78282977770978	-5.46721527103111	1.64893521933509
C	2.63241534791470	-4.98233012791498	2.62635122953151
C	3.35192862121298	-3.82526868671491	2.40252950461417
O	2.42740461933650	-0.57343568799216	-0.11382319187842
C	1.44301391957940	4.14561523541838	1.69884341279254
C	0.08459784032229	3.87866593235215	1.61797460904392
C	-0.81160600409591	4.50771542112456	2.48253791746794
C	-0.31666367643053	5.36046930624848	3.46896083272698
C	1.03680752099528	5.61956743109604	3.56220110912783
C	1.91261413913721	5.03241726138498	2.66843346083133
O	-0.58398935214764	2.05383656195991	-2.69370750854954
C	-0.42837581627222	3.38320335244477	-2.30173692567106

C	0.80771656637210	4.03545821906635	-2.52439654711501
C	0.96788060520630	5.32105603718223	-2.06291088545585
C	-0.04222557466211	5.99002939898548	-1.34706949380728
C	-1.32194625167612	5.38564678167221	-1.25492846324814
C	-1.51312567760187	4.09222671208221	-1.81381294956273
C	-2.35211092338261	6.07772759157306	-0.58484931566192
C	-2.12286653625286	7.31041384364208	-0.03702660751666
C	-0.85728662911893	7.90770204983059	-0.12731707563251
C	0.16142935358379	7.26117338058774	-0.77022973147382
C	1.85332232257118	3.45712947629655	-3.39292104995866
C	2.21243572237497	4.20250234896399	-4.51622002553727
C	3.16512729851594	3.72446159302403	-5.39471596446239
C	3.78616170529676	2.51559415850595	-5.14837685446473
C	3.46457637322689	1.76628774908130	-4.01643522917277
C	2.47983464264960	2.24339502750615	-3.15108958614541
C	-2.85530103291797	3.51219493641469	-2.00092179944996
C	-3.09262343170551	2.22548963117071	-1.56554592953560
C	-4.27746133241654	1.53793960841477	-1.90831101007477
C	-5.25563646423441	2.22582140765874	-2.58684002579154
C	-5.08342244417694	3.55805209903347	-3.00470951781638
C	-3.84097561060038	4.20201749351439	-2.76563369585205
C	-3.63076187552911	5.47332471540947	-3.34324785325565
C	-4.62382741159586	6.10182349235478	-4.04401873213598
C	-5.87607051301404	5.49327515560348	-4.21399519923562
C	-6.09487064293301	4.24006309471194	-3.71571732620057
O	-2.11926003422207	1.63128224011439	-0.76302489620152
C	-4.47273953030469	0.09290798859134	-1.67222961258787
C	-4.58593298703263	-0.72929240736092	-2.79218552071283
C	-4.80637778820061	-2.08523205697011	-2.64331014956191
C	-4.93182389125787	-2.63240366136174	-1.38084235538622
C	-4.83825714275249	-1.82392093951470	-0.25277735663284
C	-4.60999106759665	-0.46055561276165	-0.41083905119563

O	-1.79147127053463	-2.68842826360953	-1.60578025946189
O	0.33899980239217	-1.69616174067997	-0.83755545575746
O	-1.71924929550106	-0.67060709323605	0.25408852831908
C	-1.91592403255515	-0.71351124230658	2.84839263408873
F	-1.99066014688533	-1.65145114996967	3.81297556915711
F	-1.36245860483381	0.36067914058764	3.44075749334085
F	-3.17644625896660	-0.36947002330386	2.56851263412627
F	0.46705567510686	-1.32834653340896	-4.17715034835460
F	-0.75873823313320	-3.08822456018389	-4.11549544535557
C	-2.26273614010122	4.32626229627825	2.34097558620106
H	-0.27537122900161	3.20718247547239	0.85191058368552
H	1.40892263661687	6.28523916706225	4.32719281655353
H	2.96952906176856	5.25218058568345	2.71708871374776
H	3.99376559229748	-3.43751344698256	3.17983098483774
C	0.76543643694775	-5.34296139344257	-0.59970819521587
H	2.28520798372282	-3.12250927963896	-0.72867302016585
H	4.51094833622595	2.13686871051668	-5.85307095971879
H	1.71878808282531	5.14395170790332	-4.70885838400283
H	2.20864880441309	1.68086870704095	-2.27025836997147
C	4.17637774459318	0.50860836912113	-3.75001528050885
H	-4.47246814280786	-0.30065061086775	-3.77825365025517
H	-4.87908302904742	-2.71808718505256	-3.51605237567435
H	-4.51002530904265	0.16075015764074	0.46482411056068
H	6.06794440574706	1.13492678704026	-1.37261800207336
H	7.29351598064651	2.68236319285067	-2.82293810807832
H	6.61354949555601	5.04658975031628	-2.99151447086334
H	4.70384639309019	5.87580121070106	-1.67467081732756
H	3.00703053871125	5.48991460946010	0.07290269756283
H	6.23336336553415	2.44813470749249	1.18583109111956
H	7.94062255108979	-2.05585993144470	2.27015997953282
H	5.77267608252070	-3.00532985707372	1.77726651147047
H	-3.32217319070119	5.61322012460351	-0.49893046162747

H	-2.92169428280804	7.82955279231525	0.47045646733894
H	-0.69518975931787	8.88036974167228	0.31293273462450
H	1.13888927807923	7.71431341171753	-0.84793451642519
H	1.90005783166052	5.83130410800583	-2.25303843604030
H	-2.66946023873873	5.94956892181153	-3.23548290220166
H	-4.44325155141022	7.07486227067202	-4.47603829095619
H	-6.65301842912493	6.01061707049899	-4.75671348465095
H	-7.04171677764543	3.74202405747783	-3.86576422130962
H	-6.17539146932895	1.71564222179886	-2.83710399618674
H	9.38827682540481	-0.07456466359311	2.54989378496687
H	8.49148961770281	2.17307782501106	2.05615027663932
C	-4.98409430676846	-2.39752521586468	1.09174304223601
H	2.73666501082815	-5.50835086295364	3.56458449751091
H	3.42077793478423	4.29378810218248	-6.27657623163927
H	-0.99851931366843	5.81401176875881	4.17222636596290
H	1.22190140689669	-6.37700602991185	1.81081988511123
H	-5.11329519494498	-3.69084274364447	-1.26342269066395
C	3.55843144965630	-0.56796716272368	-3.12284835441959
C	4.24630576671561	-1.74458827413959	-2.88188318332822
C	5.58019400187140	-1.89726348999382	-3.24061832876969
C	6.19665130942522	-0.81413423676015	-3.86470158502973
C	5.51280574163356	0.35513480662347	-4.12150264224131
H	2.52030348370321	-0.49841517192031	-2.82804506824856
H	3.70805015012383	-2.54881008896581	-2.40909940896711
C	6.37681020524790	-3.16552612250739	-2.97578608255772
H	7.23368945855892	-0.87757051226665	-4.15874380465635
H	6.03192091030450	1.17386814017227	-4.59618070472910
C	1.22655379873674	-5.64155561420508	-1.87461625869337
C	0.37572423269979	-6.15667158054828	-2.83609608710595
C	-0.96693122675211	-6.38822216807610	-2.56237141123829
C	-1.41384652817962	-6.11046259430258	-1.27190741635568
C	-0.56959482982030	-5.60293842212271	-0.30634141239504

H	2.26559512400263	-5.47057649415205	-2.11757971966205
H	0.78214552043842	-6.36265324763347	-3.81343146413142
C	-1.94440996105394	-6.92555300771099	-3.59629108444536
H	-2.44740158060833	-6.28208481900293	-1.01073954065396
H	-0.95294175673991	-5.35649428525207	0.67308373440223
C	-5.82909964239067	-1.82079606871763	2.03611677669051
C	-5.95569699940552	-2.36799565340072	3.29639423514396
C	-5.24547187606604	-3.50706020907227	3.67279471404307
C	-4.39800805513984	-4.07311786898466	2.72562666000181
C	-4.27384126943292	-3.53579778774445	1.45648716127826
H	-6.40115537570013	-0.94366962902246	1.76940626972691
H	-6.62366517828525	-1.89000506227379	3.99763919309885
C	-5.41386824034219	-4.06286640698052	5.07970965386761
H	-3.81125092491243	-4.94622660916157	2.96227336928402
H	-3.58524743560868	-3.97032599378260	0.74628354499494
C	-2.82397757100266	3.11029612807453	1.96970071427573
C	-4.19322523279885	2.97724661304712	1.81906183546154
C	-5.05780278625350	4.04777109802726	2.01203160657155
C	-4.48887056377423	5.26271339644221	2.38956464598365
C	-3.12726997781494	5.39882944121816	2.55885250656356
H	-2.19010776704936	2.25192952250413	1.79558868418714
H	-4.57313091716753	2.00821458444874	1.54221264681670
C	-6.56172429042821	3.95304761215200	1.80640611884683
H	-5.11591713915423	6.12717757124432	2.54845103020580
H	-2.72315640063510	6.36182178952255	2.83307715032160
C	6.95601746267961	-3.68831196470021	-4.29963657177371
C	5.52330688041941	-4.27906402690091	-2.36182147178261
C	7.51733795171448	-2.83417977483624	-2.00118314615045
H	6.15358719614123	-3.87995655144554	-5.00966714493711
H	7.49526731052229	-4.61800718045459	-4.13000989531626
H	7.64244251045831	-2.97460284660953	-4.74600092285545
H	8.09166578991523	-3.73100660485102	-1.77889490071829

H	7.11108966823301	-2.44165563376474	-1.07138555637329
H	8.19026536992384	-2.09130699350403	-2.42081799764311
H	4.69870530034078	-4.54185350893943	-3.02150123933455
H	5.12089764587525	-3.98393765151410	-1.39568112065260
H	6.13376634125159	-5.16834348056869	-2.21477839543108
C	-4.92346945474238	-3.00853144447834	6.08422140657666
C	-4.61639024408698	-5.35078150897394	5.30564907622857
C	-6.89711724026966	-4.37449447869855	5.33154833011310
H	-4.79043646740375	-5.71223033446911	6.31731830480518
H	-3.55069099229245	-5.17423592388054	5.18752571504211
H	-4.92776699744480	-6.12764980164042	4.61107757988671
H	-7.25784909712784	-5.09469249508918	4.59990244964959
H	-7.51095440877151	-3.48131106040499	5.25981084258617
H	-7.02644927838748	-4.79695322631762	6.32586893731253
H	-5.51924097423000	-2.10153710963520	6.02463365491946
H	-3.88798564760061	-2.74656488456426	5.87457793023938
H	-4.98925360317201	-3.39653713176474	7.09865455934726
C	-3.14255056750229	-5.96822567810906	-3.69136597244580
C	-2.41977974153633	-8.31932315505575	-3.15786756985811
C	-1.32072698309990	-7.04174825954564	-4.99066886469270
H	-1.57509166457927	-9.00303897373598	-3.09957956973214
H	-3.13757496581643	-8.71348398939124	-3.87433931867453
H	-2.89416898473616	-8.28012538827125	-2.18088677660340
H	-3.79385046854146	-6.26034887291764	-4.51335520456520
H	-2.79483829127081	-4.95046952461396	-3.85999144661407
H	-3.72809099713693	-5.97200141852854	-2.77639903119412
H	-0.49317505784922	-7.74752011382447	-4.99566085956898
H	-0.96796766479637	-6.07132850495028	-5.33354773436498
H	-2.06969094841827	-7.39854199005766	-5.69518111849794
C	-6.94934382650313	4.86938954893757	0.63531437359724
C	-7.28478836053508	4.40401127213421	3.08499537092973
C	-7.02336571079344	2.52994818627841	1.47868795930985

H	-6.58639158544613	2.17759417359020	0.54686117159950
H	-8.10613714441085	2.51455638389990	1.36692340064185
H	-6.75652683750552	1.84230544239827	2.27880069743485
H	-6.69569856418700	5.90529713040670	0.84427020513144
H	-8.01969466045320	4.80923011503365	0.44962526077771
H	-6.42519181173555	4.56544072372900	-0.26902895897368
H	-6.97602331915015	3.78771313993210	3.92724724949123
H	-8.36092365432327	4.30363910396619	2.95995306455162
H	-7.06739883010419	5.44127568090967	3.32263562127170
H	-0.86117083884275	-5.65958289647986	4.80832726857213
H	-1.24921199300792	-3.88647561140669	4.63370890250776
C	-0.46446407559426	-4.66460606681748	4.53981150859111
H	-0.11740957188969	-4.65424542315580	3.48678817132434
O	0.60673706861972	-4.42305533886041	5.46082612936477
C	1.36559262751847	-3.32247306712072	5.33566735220626
H	0.50593725720251	-2.49687039233839	3.47955009753258
C	1.23582172164991	-2.37256677677743	4.29253591101800
H	2.43749458656875	-3.91801409219654	7.12358124882728
C	2.36386900454427	-3.15401658088583	6.33697503835193
C	2.06976761657574	-1.26043161361580	4.27421661062820
C	3.19483101871875	-2.05061028983721	6.30230933740554
H	1.92552179899889	-0.55932041176347	3.43751728994007
H	3.95527659023340	-1.91378316624476	7.08553351845348
C	3.06649273794212	-1.05545892263202	5.27844738193573
C	3.89421571672105	0.10347642419500	5.31018381662281
H	4.64037730269726	0.15189217573328	6.12322328214750
C	3.77627535794273	1.22654506764082	4.46694191943295
H	3.19322938010641	1.09559330221987	3.53916299883866
H	4.54483115248535	3.19961263491937	3.97059670146870
C	4.88359949818308	2.24588975587170	4.41596487968704
H	5.31062073511542	2.44048877040260	5.41949432180074
H	5.69722652210363	1.85650548581923	3.76619650766057

TS → (1*S*,2*R*,4*R*)

C	1.68336190354995	2.61139653696272	5.89661064373297
C	0.81681598066642	0.93730553322526	7.55142068803318
C	0.64885273364724	0.53173295437764	8.87827710259238
C	1.06529422167194	1.36522687919602	9.93394995227286
C	1.65106208981893	2.61547004184160	9.65664896933439
C	1.82875354215967	3.02361444621339	8.33329461866331
C	1.42411481479332	2.19103708917793	7.24965998896855
C	1.50699126690709	1.82906184182638	4.75110191182910
H	2.16999244949737	3.59322986169026	5.77381513258556
H	1.60761819735855	2.29415480044309	3.75877630215032
H	0.82096335885095	0.96802023423268	4.74548893552684
H	0.46180623703243	0.29416118068921	6.73313426462633
H	0.17849198580619	-0.44044358892389	9.09036622788111
H	0.92435517984387	1.04131090728280	10.97646239221669
H	1.97014164990227	3.26885091137096	10.48242324274753
H	2.29436436546643	3.99542356534426	8.10882000155144
N	0.41201025865146	-0.67183140064426	1.59557018856879
S	-0.91463130207774	-1.38699871946656	1.20213788021508
O	-0.90448389053265	-2.81579183224853	1.31302436574795
P	1.19619918161850	0.16030399895083	0.45468341134795
N	0.47723344141166	1.09689838548236	-0.61576446991037
P	-0.84688159750318	1.01143637937934	-1.49987842179292
N	-1.27042943349297	-0.27661374870301	-2.37145177792487
S	-0.80136941957625	-1.66466189328576	-1.84991959952631
C	0.06701712280223	-2.20503629957015	-3.49343959584663
F	1.15571455536517	-2.96699598108717	-3.31047382601833
O	1.97144579963366	1.21789833547849	1.42641646084825
C	2.82062184695210	2.09344371563695	0.74620884671332
C	3.99181598263567	1.60946840057749	0.19555793444927
C	4.77688649620677	2.44483226569749	-0.64717041815769
C	4.41630906604220	3.80949162859813	-0.78466908894512

C	3.35142778698879	4.30578480237012	-0.01035914375914
C	2.54259320354024	3.47982009653088	0.73365702890432
C	5.88554866808703	1.96093882461513	-1.37205646179999
C	6.60106673532757	2.79247811397693	-2.18910771555043
C	6.24139629105211	4.14066984642681	-2.32879000349631
C	5.17170892114299	4.63901370279690	-1.63934860589114
C	4.46344968326241	0.27665148022494	0.61204451191568
C	3.62926052601361	-0.81783235020771	0.50922431493028
C	4.01929567204255	-2.08675689683456	1.01189787455921
C	5.30186085177791	-2.21664485224229	1.49987392497642
C	6.18336923975822	-1.13031808418562	1.62406304856885
C	5.74096347761737	0.15931961439418	1.23580627724524
C	7.47365088062773	-1.28920985817785	2.17622859382708
C	8.28040719944912	-0.20707379970628	2.38649542133673
C	7.81413566171746	1.07987697802996	2.07810200890797
C	6.57716202962512	1.26134644653353	1.52161845770426
C	3.14624623871561	-3.27304609635787	1.10595604152319
C	2.33845202363332	-3.73236586158264	0.07299856067941
C	1.58977190633442	-4.89648568649568	0.22150296121291
C	1.67316512938414	-5.61959400464667	1.40791979116162
C	2.47811033600062	-5.17343163441380	2.43990412663740
C	3.20069131978393	-4.00589470818503	2.29513798487271
O	2.42108745973544	-0.65787385015413	-0.16316042494825
C	1.55264545716225	4.09721806772561	1.64040757706424
C	0.19491787013765	3.81327933063780	1.61976501940778
C	-0.67674324301805	4.46573055439099	2.49200774863970
C	-0.15884570090602	5.37377735406647	3.41488681911298
C	1.19313958345935	5.65542039451251	3.44318502889102
C	2.04543595999458	5.03217344335856	2.55241046859627
O	-0.61765763369870	2.09182030150450	-2.68508373693437
C	-0.42233722270131	3.40837719999672	-2.26488092085610
C	0.81843118948483	4.03879680195097	-2.51833288564619

C	1.02281721669281	5.31201224033644	-2.04225325416886
C	0.05349905935028	5.98705275534098	-1.27719614893476
C	-1.23285231739951	5.40457656777831	-1.14418595169173
C	-1.47282494376035	4.12586290853042	-1.71838514485021
C	-2.22248146317800	6.10501211136724	-0.42334169639583
C	-1.94861500201112	7.32459988206537	0.13291937810776
C	-0.67575509855167	7.89899769974146	0.00399345046001
C	0.30446657672588	7.24385089959132	-0.68773909283745
C	1.82267337942975	3.45059260551070	-3.42926047885219
C	2.11744922490794	4.17485714688502	-4.58402377142885
C	3.03682171620888	3.69025396106190	-5.49427780226307
C	3.69064503590462	2.49891104936163	-5.24695076704460
C	3.43220400596681	1.77166111142955	-4.08508599154937
C	2.47735306541126	2.25199210587011	-3.18876005496912
C	-2.83260781013641	3.57432906739719	-1.86276812219368
C	-3.07439814694006	2.27885566769854	-1.45635809839317
C	-4.27753689626371	1.61632976031940	-1.78233174994987
C	-5.27348119335290	2.33932541456823	-2.39475907011128
C	-5.09990182428384	3.68380617914570	-2.77115612964088
C	-3.83744028948569	4.30063721975294	-2.56698483362372
C	-3.63045629338802	5.58402134564014	-3.11890297042973
C	-4.64202071751029	6.24885980011702	-3.75674226825498
C	-5.91123883467391	5.66637734974077	-3.88787143345513
C	-6.12950499118378	4.40269453805125	-3.41679186430562
O	-2.08373787307169	1.64406200316263	-0.70700318389005
C	-4.46890972458123	0.16052437994129	-1.61744959086140
C	-4.59040723925331	-0.59636296773032	-2.78186310773564
C	-4.81311332106353	-1.95799739638737	-2.70880343766355
C	-4.93546753175933	-2.57392075462518	-1.47859611767966
C	-4.83015758859042	-1.83220876256498	-0.30538749850779
C	-4.59588137015612	-0.46240020090384	-0.38817489482688
O	-1.78975873253911	-2.69079788083452	-1.74205576726778

O	0.32230246054263	-1.68723805550303	-0.93664637445289
O	-1.74213433476475	-0.73696756123703	0.22027553537539
C	-1.90271130972419	-0.97213806816082	2.82867547058470
F	-2.20829615582147	-2.07217650504194	3.54194340592952
F	-1.23024478427846	-0.17198710006657	3.67762887472416
F	-3.06340285929874	-0.35516332743439	2.58284200126956
F	0.48139611308747	-1.14758835531345	-4.21454457114408
F	-0.75316066575414	-2.90176273882414	-4.29831015720344
C	-2.12748419466675	4.24678546875640	2.42108708231787
H	-0.18446197731278	3.10511590446258	0.89729706499478
H	1.58263286141729	6.36619033894220	4.15734117579342
H	3.10294952997118	5.25294955620680	2.56380943209336
H	3.80170899191072	-3.64605117903617	3.11746405151162
C	0.71562845431220	-5.38052106141410	-0.85751335962662
H	2.26015536189301	-3.17375948447353	-0.84553455468413
H	4.39217793047716	2.11619936767760	-5.97287288977356
H	1.60200567156962	5.10546329788863	-4.77294101374513
H	2.25284159762309	1.70465473651384	-2.28557292250141
C	4.18147610604566	0.53676130775040	-3.81589328760107
H	-4.48396251916239	-0.11189401944902	-3.74248805463104
H	-4.89284527166515	-2.54082413120545	-3.61515864790791
H	-4.48444904569205	0.11127733183175	0.51790880443219
H	6.15356661688539	0.91940533507125	-1.28736844093319
H	7.44843936495698	2.40866057076261	-2.73650763773926
H	6.81490433351768	4.77947264864815	-2.98386649809693
H	4.88751615988516	5.67624563838910	-1.73925491437585
H	3.15857570578575	5.36789863281547	-0.00619997649847
H	6.23054495729626	2.25902181512179	1.30610735019929
H	7.80141119053376	-2.28443544168031	2.43816878412433
H	5.64339427679044	-3.19047508836695	1.81927512092186
H	-3.19701432699258	5.65681516351788	-0.30672147587839
H	-2.71764472395446	7.85195460998546	0.67680821353974

H	-0.47792882299452	8.86101496386891	0.45286512519984
H	1.28727248946279	7.67896967536738	-0.79557470929294
H	1.95736480174798	5.80712840130939	-2.25994897088889
H	-2.65708643323928	6.04114288061766	-3.04177746122827
H	-4.46299931442397	7.23056492787373	-4.16948035419073
H	-6.70208160000856	6.21196350312693	-4.38056753126074
H	-7.09008385535652	3.92391930604571	-3.53984270289062
H	-6.20752223418909	1.84829881428255	-2.62997108362200
H	9.26676287876193	-0.33183197153946	2.80734238566416
H	8.43930158241484	1.93506556474792	2.28692759323281
C	-4.99442032501643	-2.48674980910562	0.99838954271798
H	2.54731861003645	-5.73736246248597	3.35962349680056
H	3.24305351489798	4.24208534217755	-6.39994740504754
H	-0.82081326104648	5.85113823316262	4.12121331206443
H	1.11565622619935	-6.54115141516793	1.50290863036336
H	-5.12679356304335	-3.63550303871674	-1.42226865927742
C	3.61002644985321	-0.53923288537853	-3.14539235631060
C	4.33668475113878	-1.68939642033196	-2.89157922068977
C	5.66495306590830	-1.81402291730527	-3.28105548846738
C	6.23230326020024	-0.73394962441628	-3.95513153021375
C	5.50971563630550	0.40918038072319	-4.22363071288970
H	2.57914585641136	-0.48875434325871	-2.82321413259543
H	3.83460289311261	-2.49425546791335	-2.38124912254012
C	6.50926261973378	-3.04500226781966	-2.98912621880259
H	7.26203071420794	-0.77788721422217	-4.27713177837249
H	5.99196584292044	1.22924560146327	-4.73410830093281
C	1.19140697559445	-5.58817209547878	-2.14489181232842
C	0.35325636873685	-6.04006700647512	-3.14853453088509
C	-0.99078780396856	-6.29668214588778	-2.90606891296438
C	-1.45221428027814	-6.11220938362038	-1.60395372752018
C	-0.62107156538746	-5.66731513323362	-0.59733939452626
H	2.23132227666513	-5.39397296598741	-2.36598343998685

H	0.77101277569391	-6.17493720176731	-4.13346796631404
C	-1.95590162228577	-6.76058854401577	-3.98621356266199
H	-2.48738015111391	-6.30688517190004	-1.36633062584003
H	-1.01645251453078	-5.48890726175111	0.39208972898864
C	-5.77775425453148	-1.91605679291625	1.99894337241202
C	-5.96089483203899	-2.55815682579204	3.20617818347513
C	-5.37806784236991	-3.79641899688443	3.47130202356606
C	-4.58124014817125	-4.35161976439931	2.47489947429460
C	-4.39178136465840	-3.71266290860233	1.26243134260205
H	-6.26525081970983	-0.96975339894928	1.81513510816112
H	-6.57924801840594	-2.08003208413471	3.95143952709691
C	-5.62823004023770	-4.46915771901212	4.81305285103794
H	-4.09086888050892	-5.29979003167144	2.62761178312820
H	-3.74204323781565	-4.14639188623536	0.51575113701934
C	-2.67437306161325	3.00386861194054	2.12503086852340
C	-4.04489157559173	2.83687922596680	2.02814185654083
C	-4.92496179228308	3.89754794132760	2.20534327732198
C	-4.37049004939881	5.13736062121498	2.51772092955219
C	-3.00738841562672	5.30814033189147	2.63206283659710
H	-2.02904193508270	2.14969550045322	1.96924433934205
H	-4.41052910145438	1.84819025788123	1.80816042014204
C	-6.43307132512991	3.76775712908141	2.05732340310125
H	-5.01090737309906	5.99351849100199	2.66806692834362
H	-2.61549316617487	6.28878355131119	2.85563755649629
C	7.08114696951170	-3.59687375515399	-4.30412136649289
C	5.70790458296780	-4.16337224991626	-2.31642441670443
C	7.65592760732953	-2.63770464988977	-2.05070858410208
H	6.27255318401941	-3.84417028147159	-4.98956795766586
H	7.65739478577228	-4.49941878192381	-4.11104107522468
H	7.73222638284655	-2.87673312811704	-4.79124228586715
H	8.26368604546724	-3.50545967064328	-1.80338688003928
H	7.25466483926451	-2.22094474099951	-1.12906958122060

H	8.29621081561471	-1.89129968160063	-2.51303433025748
H	4.88006523250657	-4.48004107928666	-2.94747951606011
H	5.31514674344750	-3.84569222771667	-1.35370819595234
H	6.35260306570972	-5.02445039666965	-2.14951303971245
C	-5.04514412284085	-3.58172352989395	5.92349090463179
C	-4.98080803055747	-5.85362573092354	4.91056226949263
C	-7.14103620077213	-4.64021298094265	5.02115842957684
H	-5.21759985088625	-6.29716781830846	5.87572947942095
H	-3.89987548988438	-5.78458628754778	4.82665813832880
H	-5.35789772109579	-6.51476424398530	4.13357792446678
H	-7.56488469356254	-5.23721903252097	4.21603715541957
H	-7.65296349098139	-3.68246533020694	5.03901425339395
H	-7.33313181293690	-5.14589948762601	5.96520629655227
H	-5.53754157393369	-2.61329804189855	5.95174865285269
H	-3.98334351793490	-3.41487446319327	5.75190877030170
H	-5.17213917488981	-4.05987011177443	6.89254389588774
C	-3.14386685911654	-5.78735541514681	-4.03925225291988
C	-2.45051710433254	-8.17470171990511	-3.64476467628830
C	-1.31120458620245	-6.79594430945193	-5.37538510395312
H	-1.61135579973026	-8.86658743439486	-3.60589821657356
H	-3.15102862335821	-8.51983836222739	-4.40243080461876
H	-2.95131957934918	-8.19166512981529	-2.68050767266795
H	-3.79116423083218	-6.02870041638858	-4.88062883453901
H	-2.78511003891050	-4.76553625269085	-4.15060438718834
H	-3.73638614157876	-5.83344370920187	-3.12997575173356
H	-0.48750901868797	-7.50550144906374	-5.40989161345085
H	-0.94801052462952	-5.80902994552883	-5.65409737444008
H	-2.05100751251207	-7.10481083076620	-6.11151913977376
C	-6.88577862572575	4.65993937820863	0.89091050847595
C	-7.11658432112292	4.21915158133959	3.35735664544093
C	-6.87496469802784	2.33154917633057	1.76297084808413
H	-6.45918161450889	1.97779342754978	0.82204388175713

H	-7.96015897773023	2.29207797233727	1.68556572339355
H	-6.56893105576028	1.65988660804183	2.56269652384083
H	-6.65991235222541	5.70562063589765	1.08159523473563
H	-7.95883912162904	4.56391227508982	0.73919901596596
H	-6.37979028537108	4.36361103015878	-0.02613565374607
H	-6.77317948613234	3.61097146964212	4.19204959613148
H	-8.19531028334108	4.10746758733372	3.26975570998432
H	-6.90065824412603	5.25980730317866	3.58136176200483
H	-1.32600746999022	-6.27752290786273	4.55514395024153
H	-1.61172867030351	-4.54375127733139	4.05633530611033
C	-0.85073756726519	-5.32300554453938	4.26766714939712
H	-0.24351332008746	-5.45140333479697	3.34928660376048
O	-0.03023859868649	-4.95715166783077	5.38533528290957
C	0.71311635725739	-3.84026263645887	5.32735221824198
H	0.19656540164204	-3.17320476392494	3.28583610597717
C	0.76835303021350	-2.97586177761224	4.20535859683997
H	1.41487124650084	-4.25181910580432	7.33688795163660
C	1.48145671529235	-3.55342752614077	6.49068565272323
C	1.56690361757398	-1.83949201695482	4.25876761661253
C	2.27239531269160	-2.41916461890441	6.53196910620131
H	1.55908170901645	-1.19144732353559	3.36807407899156
H	2.85497391110004	-2.18965708817999	7.43686961721202
C	2.33695751759754	-1.51910254495500	5.41854498471710
C	3.13208937698551	-0.33722864582926	5.51014786124576
H	3.66591623779184	-0.18539635664099	6.46514227579732
C	3.25601694427348	0.67613745440614	4.53422970877106
H	2.90536402274274	0.43128815670501	3.51642099302831
H	4.21472543407200	2.54133010092755	3.97266888571036
C	4.39534794975773	1.66006467297926	4.61665706668506
H	4.57265309010973	1.99351444219299	5.65761851333153
H	5.32181118521285	1.16992464705344	4.24798567721664

TS → (1*R*,2*S*,4*R*)

C	3.45343251612774	0.33684733604035	7.89668070122831
C	1.58575268960360	1.17590639877260	6.43880348970974
C	0.26242629390042	1.08769093032719	6.00686437493425
C	-0.60766442309262	0.14939169197951	6.59441997297475
C	-0.14580280988117	-0.69984949743762	7.61965414585164
C	1.17804115337463	-0.61884803482725	8.04891606555562
C	2.08072689893315	0.31599735597515	7.46361491055866
C	4.47183063154904	1.13058089263135	7.35722480880472
H	3.71960008770828	-0.39305268490360	8.67958531937215
H	5.41638743965577	1.19588379657362	7.91762502757454
H	4.20611675475803	2.03010637401157	6.78232961843788
H	2.24837632696507	1.91126835267645	5.96181671429840
H	-0.09953629453493	1.73070747697419	5.19017373487945
H	-1.64772790071067	0.07740399562869	6.24081894918195
H	-0.83291975242490	-1.43208537855013	8.06966981900794
H	1.54332788785783	-1.29461258598974	8.83723026416810
N	0.46810876632719	-0.99275309686293	1.54995065756688
S	-0.96917713203189	-1.48899582232445	1.25281900284447
O	-1.15374524856654	-2.88525185741956	1.51918111285918
P	1.15010393750718	0.02328680787036	0.49534689929661
N	0.34824312115503	0.99132022529496	-0.48340538166683
P	-0.89458549435230	0.84715104809373	-1.47383235908107
N	-1.23204857251227	-0.43517815586494	-2.38956509895479
S	-0.80582743785930	-1.83014998977102	-1.84425184081274
C	-0.04310420261775	-2.45226021142959	-3.51284892372398
F	1.03842154399940	-3.23136699201457	-3.35490047674843
O	1.84828046814885	1.02955614452426	1.56832672550771
C	2.55928640415264	2.07052130545629	0.97537085204609
C	3.74982253887379	1.80803420759714	0.32327869887052
C	4.37494956469301	2.85038124196219	-0.41922928456009
C	3.85017243818374	4.16614471166308	-0.33833964662794
C	2.74984603167801	4.41345431086964	0.50657701297957

C	2.08708684407831	3.39064346491495	1.13607213866518
C	5.48545683808058	2.61688733634277	-1.25756879279661
C	6.05209106162851	3.63781342972573	-1.96956942876579
C	5.54637717875394	4.94196373363768	-1.87339955193214
C	4.47108301064035	5.19980277731383	-1.07043246796686
C	4.39379048056547	0.49927139838241	0.55065171436462
C	3.67229228233974	-0.67038031337579	0.40151726935449
C	4.20104965355180	-1.92035765652130	0.81764613294513
C	5.52187532745959	-1.96181461504645	1.20510052731952
C	6.31294619779680	-0.80677847827586	1.32240179027707
C	5.72066309726183	0.45884406997777	1.07910127008067
C	7.65633776039705	-0.88019380174120	1.75155219391622
C	8.37691139242694	0.25591886328195	1.98916033872784
C	7.76370363974280	1.50956177271071	1.84483919923981
C	6.46989920546156	1.60978425678106	1.40983571037236
C	3.40276706304557	-3.15115381110628	0.98008905620600
C	2.51746291193700	-3.65295892431776	0.03407226680586
C	1.79639901010406	-4.81677793612069	0.28489693054471
C	1.97556101113518	-5.49158724076414	1.48864260721284
C	2.87611296745282	-5.01667467634096	2.42380893441767
C	3.58109287613290	-3.85610848170287	2.17404517165476
O	2.42733553678419	-0.61152260554665	-0.21686803670356
C	1.01497669047882	3.70384278675243	2.11134123024545
C	-0.33365024470162	3.67534261171519	1.79466932244656
C	-1.28726940616115	4.08305153691667	2.72664259493681
C	-0.87038256747222	4.48031128380958	3.99514032750838
C	0.47178960453148	4.47716856244729	4.32599661793416
C	1.41356572140400	4.10458325016148	3.38446547808668
O	-0.61055698846086	1.93202655904678	-2.65048461384953
C	-0.49654311902022	3.25807376620297	-2.24190740221188
C	0.73442855670768	3.93910744401807	-2.40323361011175
C	0.84210987348217	5.22465111551651	-1.92356287270327

C	-0.21591213846875	5.86842714482440	-1.25662403429726
C	-1.48229658056338	5.23154664818424	-1.21839575017741
C	-1.61722753733409	3.93798242856655	-1.79490057642134
C	-2.55147529122682	5.88823150290047	-0.57441633264342
C	-2.37514774441853	7.12206796226523	-0.00980047387608
C	-1.12698512953699	7.75921497465844	-0.06233645034826
C	-0.06921738278250	7.14443898188499	-0.67272445422913
C	1.84256723580212	3.40145489741984	-3.21753984946398
C	2.29659813617441	4.21088941846970	-4.26052894641437
C	3.32817217358995	3.79024251485628	-5.07656462673510
C	3.93262240265059	2.56948309997136	-4.84855683241513
C	3.50624320099536	1.75014790238211	-3.80359276565355
C	2.44570267776594	2.17008731106474	-3.00168149530822
C	-2.93854426950157	3.32608441823228	-2.02406244115804
C	-3.16757652170810	2.03943889031801	-1.58507277050103
C	-4.35013928001593	1.34362654375466	-1.91925771618775
C	-5.32236915938536	2.01156440848637	-2.62510924579986
C	-5.15253007898043	3.33865322041071	-3.06132525385402
C	-3.92256788845156	3.99950888078593	-2.80521212878520
C	-3.72128666827278	5.27347613496122	-3.37981083198187
C	-4.71235375412909	5.88803565071316	-4.09586289070828
C	-5.95257602068218	5.26115769681623	-4.28588508359620
C	-6.16140085213509	4.00495295156924	-3.79045824661163
O	-2.19952771524961	1.45226539530653	-0.77269986407854
C	-4.55758842657545	-0.08424628600058	-1.60631474135403
C	-4.69764868082906	-0.97109635661491	-2.67198647786919
C	-4.93097840742921	-2.31265242530972	-2.43624637498407
C	-5.04046211464649	-2.77960957408381	-1.14055217805242
C	-4.91790562102266	-1.90549099029169	-0.06474140563498
C	-4.67901972610540	-0.55648876149939	-0.30986557124117
O	-1.82075518507548	-2.82213888280081	-1.66834431183422
O	0.35117162522081	-1.86158180817345	-0.97945449634209

O	-1.71689629368533	-0.85259796054746	0.19903472608559
C	-1.83620623359559	-0.70690112652644	2.79269710863490
F	-1.78327442254045	-1.48970601282007	3.88711240561720
F	-1.25006424801967	0.45201514542861	3.13575268133729
F	-3.12839703337638	-0.42532674624305	2.60021918356768
F	0.35273944313115	-1.44222379804196	-4.30865387023765
F	-0.92011645491555	-3.16758374823062	-4.23756509802466
C	-2.71360816088306	4.13716292033716	2.37249883735738
H	-0.64018753951679	3.35621597124863	0.80920898245476
H	0.78299614869052	4.77558223716024	5.31693468642470
H	2.46710387337838	4.12699224463727	3.62463624390454
H	4.26094823876480	-3.47431657410990	2.92109143704011
C	0.85175705026124	-5.36329194441531	-0.70471170747615
H	2.35337336563680	-3.12547397272341	-0.89046482364293
H	4.72696868424468	2.23479931903099	-5.49891512573273
H	1.81622140525794	5.16119132048528	-4.44214769939821
H	2.10214624115578	1.54836424384490	-2.18789984987753
C	4.19081324425874	0.47031175891328	-3.56998126117387
H	-4.59433192671819	-0.60526088223626	-3.68407623511126
H	-5.02514754799541	-2.99663960944963	-3.26721762206883
H	-4.56126917644542	0.11763077664556	0.52481332506350
H	5.87100886685985	1.61285530181046	-1.34201357757347
H	6.89757526859733	3.44421612769872	-2.61231864587993
H	6.01112001439396	5.73663198702387	-2.43768824880887
H	4.07582900659313	6.20162927348680	-0.98539781299520
H	2.41637766845362	5.42935849896039	0.65833295078099
H	6.01432871538314	2.58259877863176	1.32273947037888
H	8.09618349734759	-1.85605911786163	1.89579603634691
H	5.96169577870891	-2.91394375823951	1.46503884931051
H	-3.50795947402014	5.39351903035094	-0.51600829466746
H	-3.20222673881544	7.61011286885223	0.48277054253066
H	-1.00845511704358	8.73524725325764	0.38436149844083

H	0.89722841706024	7.62513144214936	-0.71749053145022
H	1.77379468142379	5.75325723206126	-2.05656971514837
H	-2.76841014679907	5.76302075179101	-3.25682359195436
H	-4.53943493364298	6.86409242674095	-4.52420563212368
H	-6.72837171814367	5.76745338417903	-4.84051434593077
H	-7.09921075766446	3.49417070652967	-3.95390684819425
H	-6.23851217442652	1.49327825330410	-2.87243275786216
H	9.40617638028723	0.19693086211274	2.31035721464890
H	8.31938340894145	2.40393633401075	2.08404179359369
C	-5.03408139952007	-2.39419782096344	1.31538553539270
H	3.02737091565158	-5.55091422888945	3.35076370632564
H	3.65891040896888	4.41406800702206	-5.89419803147966
H	-1.60700535823042	4.77505275744034	4.72847897089011
H	1.41616359598722	-6.39768392481656	1.67442949164878
H	-5.23261259244935	-3.82670946557688	-0.95563447665189
C	3.49832001573285	-0.67520697629402	-3.19574200770442
C	4.15815417669283	-1.87963705974884	-3.02042747110175
C	5.53424264768010	-1.98816737498097	-3.18265048864798
C	6.22507174492479	-0.83246331041744	-3.54272247551222
C	5.57114770875599	0.36469685358111	-3.74308998222801
H	2.42645910624581	-0.63323749946947	-3.05750407512547
H	3.56478613199111	-2.73981201029892	-2.75899022561220
C	6.29764952820621	-3.29143853510775	-3.00350496225579
H	7.29580548308923	-0.86214480930223	-3.67921860058801
H	6.14096871054383	1.23840758442616	-4.02226145559503
C	1.26927052068984	-5.75584388959420	-1.96903295406323
C	0.37450615924309	-6.29164411306298	-2.87829318675318
C	-0.96945892667077	-6.45029958886850	-2.56127118658055
C	-1.37403508173356	-6.07060908985524	-1.28280073025105
C	-0.48666730746929	-5.54181823194490	-0.36841948339729
H	2.30812038277576	-5.64519850997666	-2.24523410757537
H	0.74826932210991	-6.57507566834966	-3.84927509974021

C	-1.98902892201171	-7.02402277087196	-3.53351121818603
H	-2.40757832999249	-6.17641069172707	-0.98934187479071
H	-0.83786163242743	-5.21319617812322	0.59882858260757
C	-5.81816991969648	-1.73173351035701	2.25660224302182
C	-5.89854567022962	-2.18988847935179	3.55539357237965
C	-5.19872244598240	-3.31991239691284	3.97629165695447
C	-4.41717619023204	-3.97558061266612	3.03045999604884
C	-4.34201871554706	-3.52946946808084	1.72305078967392
H	-6.37398813618848	-0.85299714546622	1.96136587787445
H	-6.51713484722357	-1.64486547805387	4.25294403654335
C	-5.29745940659082	-3.76463836224744	5.42846107786623
H	-3.84116168073688	-4.84673597245782	3.29795031266454
H	-3.69778605529312	-4.03226999717041	1.01633922705554
C	-3.32639453938886	3.10760892712611	1.66959510712611
C	-4.65996809472219	3.19002060970666	1.30866247256624
C	-5.43373510869496	4.29886595584280	1.62944335302685
C	-4.81681364183011	5.31840112619929	2.35274980450650
C	-3.48929959623619	5.24078463650927	2.72154343855668
H	-2.75559427575153	2.23016282905559	1.40210421448150
H	-5.08402683954701	2.36770936010655	0.75627689982886
C	-6.88998771631793	4.44112678028134	1.21337240368333
H	-5.37643660888707	6.19966601882568	2.62897717829825
H	-3.03639743146412	6.06041291879561	3.26097998347842
C	6.97183653302238	-3.65970593743219	-4.33454475596467
C	5.38948153540341	-4.45290065390438	-2.58977899112446
C	7.36322019733551	-3.10386209257950	-1.91338910802713
H	6.22312670949937	-3.76770801246041	-5.11721808123873
H	7.50562360311595	-4.60274321038183	-4.23576008431752
H	7.68011569905977	-2.89644706266088	-4.64471545762049
H	7.89134238202655	-4.03985971892877	-1.74397136833931
H	6.88997328780072	-2.79544396548784	-0.98426664560918
H	8.09058219269158	-2.34759246999586	-2.19462659242260

H	4.63194055501361	-4.64354089782349	-3.34742381258535
H	4.89649441236900	-4.24854223040016	-1.64172961926099
H	5.98333427502547	-5.35809686736045	-2.47429949528395
C	-4.69746622936083	-2.66140200402279	6.31422454219670
C	-4.53883262172674	-5.06710964258119	5.70024653733984
C	-6.77123676588263	-3.98979495778979	5.79926726475235
H	-4.66333028488617	-5.34800488431719	6.74430189723578
H	-3.47642247292194	-4.94685402256617	5.50614254510042
H	-4.92377398151264	-5.87651124001390	5.08412302728782
H	-7.21120496998164	-4.74075144831403	5.14611190757930
H	-7.35151735592352	-3.07650180041715	5.70576929635506
H	-6.84864259380463	-4.33725471955814	6.82746156581130
H	-5.25382316613591	-1.73297131518578	6.21511316789042
H	-3.66596131901854	-2.47040052754385	6.02344055772775
H	-4.71768016674430	-2.96494571530437	7.35898216511784
C	-3.16418442863749	-6.04367111246422	-3.66667769909041
C	-2.48999983175276	-8.37057603035102	-2.98830451340950
C	-1.40517041991848	-7.25252898165112	-4.93134962282464
H	-1.66175594706433	-9.07084434368753	-2.89821202302242
H	-3.23319293671085	-8.79502302262441	-3.66023907055229
H	-2.94161213526852	-8.24897765934132	-2.00732345319602
H	-3.86017078458201	-6.39224213408700	-4.42794515835936
H	-2.79878903933021	-5.05737709099964	-3.94673806397782
H	-3.70568471822039	-5.94050563531014	-2.73078769249106
H	-0.60202024152846	-7.98551445227206	-4.90901846370214
H	-1.02917636792915	-6.31934301334383	-5.34551370435846
H	-2.18336276921270	-7.62765171648188	-5.59336030520797
C	-7.01781510560402	5.64760949305345	0.27034647372807
C	-7.75683235125316	4.66206112764484	2.46268218661570
C	-7.41722851827598	3.20349064288415	0.48048596451367
H	-6.86044343212268	3.02177874114042	-0.43611364519376
H	-8.46163843043287	3.35639502800999	0.21484343097021

H	-7.35655665047607	2.31826571534213	1.11052621628687
H	-6.68646464599375	6.56293482799855	0.75330878491461
H	-8.05455308053008	5.77650043988642	-0.03346312656031
H	-6.41584467573618	5.49442188828633	-0.62363503022633
H	-7.63395710820667	3.83201181831135	3.15589095182299
H	-8.80635117388392	4.72521401956206	2.18285360666152
H	-7.48760212272323	5.57976802440753	2.97780545786473
H	-0.76217389923031	-5.57652127248512	4.91903342401107
H	-1.02601406442391	-3.77419228278135	4.86307039493346
C	-0.32973495820634	-4.60427434106676	4.62461033395671
H	-0.14719097950565	-4.57609274630705	3.53092350937578
O	0.88094114901519	-4.48288520367638	5.38284789596073
C	1.68865175135104	-3.42742543205273	5.18917068358773
H	2.96037101426610	-4.17151075354830	6.77770148706808
C	2.82567933778843	-3.36501837219843	6.04313402130315
H	0.64035587550737	-2.47018748838218	3.49998645234892
C	1.47958021434916	-2.42957343389739	4.20713130698407
C	3.72010686577903	-2.31498146591836	5.93178930990557
C	2.38260195577976	-1.38096947261704	4.10369448076608
H	4.59851181566784	-2.29043984070133	6.59107973995650
H	2.18370658580818	-0.61834168553157	3.33338460039770
C	3.51888752368494	-1.27692720163015	4.96487243350214
C	4.37260273115549	-0.14165726886285	4.84926582224110
H	4.10487231847481	0.57242354834317	4.04983725630974
C	5.42975386926600	0.19223902825807	5.72044100345198
H	5.82188146943901	-0.62583269438905	6.34708233918554
H	7.17387353508833	0.79917835446079	4.63273155843894
C	6.43410513820144	1.24466919130626	5.33135583668183
H	5.95563237921714	2.09081414201483	4.80036198379263
H	6.99354393240222	1.63497489437714	6.20291578644671

TS → (1*R*,2*S*,4*S*)

C	0.79777825545886	-0.35616857350874	6.66771409405710

C	2.18091822632763	-0.22495181485190	8.77396758315538
C	2.40544198323659	-0.63438700485942	10.08959321450915
C	1.52378467753877	-1.53974705481610	10.71302153959176
C	0.41010040841612	-2.03373128640897	10.00799247159890
C	0.18208404877811	-1.62993937148079	8.69088561806638
C	1.06409062289522	-0.72001663726473	8.03944076841990
C	1.59117314364875	0.44606750697679	5.84454651344063
H	-0.08985310484799	-0.81913004215309	6.20274225689762
H	1.16843608951726	0.81371928322114	4.89715175575645
H	2.37615600402042	1.07543013332257	6.29038030144403
H	2.86917685987795	0.48962619423817	8.30040639493099
H	3.27256504719987	-0.24379028358739	10.64310065440563
H	1.70415245700967	-1.85790649330622	11.75115460481871
H	-0.28088713148751	-2.74043372631920	10.49175733645726
H	-0.67685338219617	-2.02172042573648	8.12464155947640
N	0.88154504642589	-0.88049792891706	1.30608571633211
S	-0.60956143207264	-1.27501034240946	1.15634264497741
O	-0.86086760928109	-2.64139999705652	1.50513187952929
P	1.46311333947438	0.17943446039253	0.22656563109981
N	0.59437524738158	1.13204646950007	-0.70934885838825
P	-0.68156066976772	0.96147409849528	-1.65192581468959
N	-1.07162755087476	-0.34822256322948	-2.50822277776416
S	-0.57381575671331	-1.71812212120800	-1.96244401202448
C	0.06445220944225	-2.38039776694264	-3.66495372339042
F	1.13944395503464	-3.17804362696836	-3.56574228902310
O	2.16799238531721	1.20419414652441	1.27937641377449
C	2.78337941815848	2.28778250087926	0.65776525951783
C	3.95042496288199	2.10240354263357	-0.05872625611328
C	4.46362438703394	3.18859970163210	-0.82324265053889
C	3.85248019888216	4.46449408178789	-0.70986328245264
C	2.76243985649064	4.63322099546435	0.16847764123695
C	2.21299578902811	3.56641196177681	0.83177161610266

C	5.54211667244302	3.03295199942734	-1.71934235983152
C	6.00659287940729	4.09238545063829	-2.44884946866316
C	5.42876520859201	5.36196192231765	-2.30757554013224
C	4.37763635430020	5.54287881469711	-1.45290221298474
C	4.67720835375298	0.83139595028455	0.12867917666047
C	4.00531213517656	-0.37328267850436	0.03124141332790
C	4.59911332891788	-1.58643105705338	0.46486402000706
C	5.93936201767352	-1.56882783133491	0.78052564293837
C	6.68958599344196	-0.38079334481435	0.81605219221212
C	6.03018675130680	0.85522301548842	0.58831739090230
C	8.05514370067069	-0.38953617099679	1.17562672283768
C	8.73395503052057	0.78009470251616	1.36975080241039
C	8.05804144496488	2.00279568497401	1.24759858954513
C	6.74196813493670	2.04117193086560	0.87304679326192
C	3.83910216285720	-2.82216911711738	0.73532745137362
C	2.91368433425982	-3.38952316541071	-0.13180049834422
C	2.22264637043534	-4.54353891813959	0.22671729482384
C	2.47797172431739	-5.14468988845398	1.45566827151824
C	3.41285580194068	-4.60052631337281	2.31607983915997
C	4.08331045157561	-3.44663774614306	1.96259517409874
O	2.73619911486697	-0.39281048767599	-0.53780175533229
C	1.14788474788850	3.75933822352656	1.84306766696168
C	-0.18028470701061	3.95836990848530	1.50119591723625
C	-1.13398431682231	4.21334836759672	2.48334148064596
C	-0.74201708129848	4.26223044441306	3.81727021543207
C	0.57716825248142	4.03570021029782	4.16485848450154
C	1.52003104701810	3.78700655932701	3.18446303473310
O	-0.43586582064133	1.99274505334781	-2.88424033316580
C	-0.33597470040842	3.34305951280115	-2.56810660533266
C	0.87434228047746	4.03599480295460	-2.81654666487639
C	0.96546952351017	5.35539432000914	-2.43418664307655
C	-0.08724563315278	6.02585798900408	-1.78617003921833

C	-1.33580523475093	5.36607985218163	-1.65830324532490
C	-1.45721646707504	4.03055380374002	-2.13510253702929
C	-2.39811199812169	6.04451343756546	-1.02514217568500
C	-2.23323927152887	7.32024096379274	-0.55885434444493
C	-1.00458695390201	7.98058599283072	-0.70498593892460
C	0.04706141732017	7.34553856027207	-1.30498510816315
C	1.97508531550817	3.46846693001442	-3.61977517161582
C	2.41038065345450	4.23339515626100	-4.70418311652377
C	3.43975768631833	3.78926871961825	-5.51018811630047
C	4.05982057991073	2.58661145714285	-5.23340451758918
C	3.64759539583577	1.80922060685450	-4.15166512916456
C	2.59046743181740	2.25159090372116	-3.35800491262878
C	-2.77297299656057	3.37996176281819	-2.26748028778582
C	-2.96608493748241	2.12937799707543	-1.72125047183775
C	-4.16338462099695	1.40953967520251	-1.92890501734220
C	-5.17538300784821	2.01304410238221	-2.63627070571294
C	-5.03650340429107	3.29919412658880	-3.19049168091015
C	-3.80124820003637	3.98393339939134	-3.04872956357753
C	-3.64025698151412	5.21063462082513	-3.72915192065227
C	-4.67111965790547	5.76322380331682	-4.43941304630880
C	-5.91325541968272	5.11672164622052	-4.51889212478708
C	-6.08570673185853	3.90151218253266	-3.91849809354801
O	-1.95080911658099	1.60528967887458	-0.92125468811569
C	-4.35189987006011	0.02231435803074	-1.46087359119557
C	-4.54227641680491	-0.97573421015397	-2.41348296871023
C	-4.75819690494627	-2.28233546619948	-2.01823042323962
C	-4.79647181183116	-2.60339443848756	-0.67477416964110
C	-4.62353997355182	-1.61579432701966	0.29022630525273
C	-4.40632724288446	-0.30206631043940	-0.11519231909038
O	-1.54905411629398	-2.72240074852638	-1.66930843348651
O	0.64865341000148	-1.69938690002508	-1.19456058809459
O	-1.38669487752819	-0.66663575289532	0.10922389668490

C	-1.29558255183758	-0.33767149459054	2.70031521445013
F	-1.24628026360502	-1.05756422124575	3.83637576559512
F	-0.55982581951767	0.76198548407128	2.93528306245720
F	-2.55772681879543	0.07619769827634	2.56900535607071
F	0.42412759231731	-1.38920109258038	-4.49971658105633
F	-0.87171518359028	-3.09053896693457	-4.31732856315491
C	-2.54319079171478	4.43247746480113	2.11384217039036
H	-0.47531894564736	3.92080628246159	0.46324552067397
H	0.87139264680675	4.05809724896371	5.20440582231370
H	2.55527250482568	3.62602863862831	3.44837238195345
H	4.78906412279103	-3.00869369357472	2.65273578077356
C	1.22220728443994	-5.14616551474921	-0.67041091765049
H	2.69292218402612	-2.91758460626126	-1.07432669600807
H	4.85420791680396	2.23049150367893	-5.87240279661218
H	1.91707245358367	5.16840614494128	-4.92569084712574
H	2.26157730118200	1.65953178160668	-2.51632798143195
C	4.34345195735833	0.54233698035132	-3.88313493493031
H	-4.49112834435918	-0.72481006677700	-3.46373734957929
H	-4.89268488231892	-3.05326666576174	-2.76286652852984
H	-4.25262181389461	0.46378961529305	0.63025884345985
H	5.98645233370832	2.05599017788782	-1.82891054825422
H	6.82725297537147	3.95793543601751	-3.13734549894699
H	5.81870193569552	6.19121193569623	-2.87883081734036
H	3.92887276976481	6.51803190747489	-1.33158391838319
H	2.35280531367916	5.62089778478269	0.32140593720754
H	6.23866444001307	2.99145783605071	0.80212245174481
H	8.54535495149235	-1.34297774656561	1.30804845807839
H	6.42580817070315	-2.49311697874425	1.05825211086523
H	-3.33790298337383	5.53281166216927	-0.89222256682816
H	-3.05394936315013	7.82380701251205	-0.07148169958992
H	-0.89564237128336	8.99005234138180	-0.33716359151468
H	0.99970904914773	7.84209575568929	-1.41856105526423

H	1.88224626980550	5.89090836887368	-2.62840961332268
H	-2.68666684108824	5.71277054632300	-3.69303607653771
H	-4.52831955301429	6.70442047725966	-4.94916769531481
H	-6.72014783076073	5.57460783341181	-5.07124379549673
H	-7.02576367538815	3.37461780560152	-3.99548210282759
H	-6.10246603508057	1.47680771762141	-2.78462635960179
H	9.77846371814412	0.77053195409989	1.64293729925108
H	8.58280012163221	2.92348677763214	1.45492948354470
C	-4.64435578214253	-1.94330005933293	1.72212622577356
H	3.61422617997319	-5.07203712310281	3.26716671891416
H	3.75640702689537	4.38040064536611	-6.35711441997521
H	-1.48164675976121	4.45706562971432	4.58080763871962
H	1.94568604351629	-6.04603625092921	1.72298567225205
H	-4.97124172849039	-3.62450357728692	-0.36702707025475
C	3.65500424921393	-0.60927649805026	-3.52155545444504
C	4.32262516512443	-1.80866433831299	-3.34119598967898
C	5.70213211040389	-1.90406818435480	-3.48335618109289
C	6.38877695694447	-0.74008641224933	-3.82397653772998
C	5.72739320595813	0.45190061310914	-4.03088535609751
H	2.58081023755282	-0.57478967971319	-3.40120686225835
H	3.73195522804839	-2.67535750758273	-3.09490208442930
C	6.47199483669891	-3.20579660199099	-3.31967157853310
H	7.46148649547864	-0.75970146668309	-3.94502609266822
H	6.29281963133559	1.33157641293590	-4.30152530321335
C	1.54929410050852	-5.57172268187203	-1.95045168377921
C	0.59884331392372	-6.15287242581897	-2.77116160097127
C	-0.71397328543470	-6.32381376466221	-2.34811666368189
C	-1.02759631885309	-5.90915378524765	-1.05500465019951
C	-0.08356571790099	-5.33711514778220	-0.22792987837744
H	2.56118482467418	-5.45312207547986	-2.30999276906417
H	0.90380131322352	-6.46028497431025	-3.75875149736673
C	-1.79371913675301	-6.94394300192577	-3.22181285163703

H	-2.03454727027610	-6.01869129571356	-0.68141266982848
H	-0.36866900503381	-4.98124252293984	0.75091427212774
C	-5.35372214088482	-1.16774175572427	2.63589499962911
C	-5.30712718920418	-1.44856270575498	3.98601233263888
C	-4.54018715967896	-2.49936250553490	4.48686088705806
C	-3.86011225989297	-3.28722338786370	3.56469933719733
C	-3.91686744205849	-3.02418945192408	2.20784946031978
H	-5.94734632509092	-0.33740562982265	2.28049125611847
H	-5.87328075282812	-0.82199027953684	4.65913913288052
C	-4.47111863047108	-2.72998860617008	5.98972416811941
H	-3.25370734868389	-4.11661773794320	3.88910540700993
H	-3.33957238871385	-3.62271931934781	1.51826626815888
C	-3.22729253695751	3.48975488827823	1.35804218869127
C	-4.54542269067228	3.69557730663183	0.98987547052180
C	-5.22646387130823	4.85227022930175	1.35145036083469
C	-4.53552856026427	5.78590852149466	2.12237892945559
C	-3.22509358213284	5.57973380282105	2.50687958173726
H	-2.72041817780061	2.58332484461574	1.06087563533172
H	-5.02898275376347	2.93342382313701	0.40041884217264
C	-6.65615466994857	5.13667784739066	0.91462034378438
H	-5.02163666089247	6.70047960440782	2.42742865597997
H	-2.71202931615794	6.32772482985048	3.09501262495027
C	7.09250691658846	-3.58106979841620	-4.67476403595134
C	5.57950160645581	-4.36279023823163	-2.86176004872540
C	7.58123909348070	-3.01606396219314	-2.27472194073863
H	6.31241658005761	-3.70441472023540	-5.42382296377989
H	7.64076062851115	-4.51716339989456	-4.59001792355463
H	7.77739611802934	-2.81178399377299	-5.02119969711876
H	8.09748654201704	-3.95871423547110	-2.10515663726551
H	7.14959451020357	-2.67884119240727	-1.33574094194147
H	8.31341623236247	-2.28183355114585	-2.59820656270973
H	4.80468919422166	-4.57230939182986	-3.59648893446481

H	5.10986659531003	-4.14010283116150	-1.90591934342208
H	6.18005549034495	-5.26320369630117	-2.74351593377818
C	-3.99559210360097	-1.43731520066952	6.67112616532643
C	-3.49544074677414	-3.84998835455778	6.36546826602996
C	-5.86815710337776	-3.10748981414093	6.50459104052232
H	-3.45061785744850	-3.94558594167799	7.44876228322946
H	-2.49590046029497	-3.62755679664139	5.99632650053479
H	-3.82736159667315	-4.80242342747435	5.95865959192425
H	-6.21501076082899	-4.01762305506089	6.01914603473173
H	-6.58770126522979	-2.31961815175686	6.29905564355374
H	-5.84069530746433	-3.27569083692636	7.57923082797311
H	-4.71497618849117	-0.63330637547234	6.54440410172130
H	-3.05065810417755	-1.11478667787411	6.23849915467377
H	-3.85786267831564	-1.60228597648338	7.73787834034210
C	-2.98451182241435	-5.97761258450817	-3.31015264280468
C	-2.24264761005551	-8.27114234494326	-2.59041453152137
C	-1.30798777184334	-7.22513444266456	-4.64724455972887
H	-1.40252449048752	-8.96013130153261	-2.52794056763635
H	-3.02372077577919	-8.72869381478205	-3.19417236167473
H	-2.63042611150393	-8.11341873741915	-1.58763224402904
H	-3.73532541175176	-6.36799347904258	-3.99532630931450
H	-2.65182994654382	-5.00493815665082	-3.66808193030361
H	-3.45031810243289	-5.82833392989366	-2.34023939216423
H	-0.49501830253003	-7.94761740037207	-4.65200203334909
H	-0.97560821021173	-6.30659057680284	-5.12635181248296
H	-2.12605835962045	-7.63638216653660	-5.23566174538849
C	-6.63851260902301	6.32294817534692	-0.06202367980749
C	-7.51417602929191	5.48382639353276	2.14086685378858
C	-7.30345130870078	3.94206351414616	0.20667600116464
H	-6.77454774925616	3.69351951067072	-0.71029953756466
H	-8.33053055345825	4.18862392646662	-0.05584874371452
H	-7.32026051503440	3.06655193772329	0.85264991169831

H	-6.20251405971173	7.20473864334360	0.40025851725088
H	-7.65179080816179	6.56475030363323	-0.37605052571846
H	-6.05549539352492	6.07520890860847	-0.94770061236197
H	-7.47250666426112	4.67611462403170	2.86906068681244
H	-8.55080981032098	5.62573121062023	1.84244125366154
H	-7.17503030619326	6.39646232148910	2.62222633443045
H	-0.92069374402353	-8.07285096157100	6.41869850123987
H	-1.63784546840829	-6.67904116098323	5.51236439797507
C	-0.70224006000996	-7.23185817055854	5.73679261794816
H	-0.27387960484450	-7.61896891618878	4.78835321827641
O	0.23649021157319	-6.40428408440342	6.42947038713283
C	0.69894644357524	-5.28738192669245	5.84272561158073
H	1.94041392009263	-4.92786283513310	7.58401213562831
C	1.65175728293905	-4.54762237008851	6.59399268685110
H	-0.43460540658068	-5.37263438994964	3.95553121254782
C	0.30424726930064	-4.83144347369047	4.56097625140549
C	2.19538894485382	-3.38605511259752	6.07213583824404
C	0.85432131413954	-3.66147907389049	4.04246685323211
H	2.94265838872986	-2.83800940741282	6.66101947039166
H	0.52229972611552	-3.30282175112309	3.05423968894935
C	1.81276942208532	-2.90457507398241	4.78005448132921
C	2.32601195632778	-1.69921626522965	4.20673105592830
H	1.99289429910144	-1.46216377526468	3.17755806143641
C	3.11057382882156	-0.74554267981318	4.88174322004793
H	3.54934709561534	-1.04991790626696	5.84814471130443
H	4.81877881951234	-0.11179800596751	3.75077470137428
C	3.86124700573572	0.31427979207836	4.12231227302083
H	3.28588378614826	0.65780713640140	3.23883948310564
H	4.11611405975802	1.18257496356110	4.76006699447135

TS → (1*S*,2*R*,4*S*), p-CH_3-styrene

C	1.10726272419689	1.61227437544794	5.62360151917435
C	1.74537322355584	0.79266434938850	7.91863636334005

C	1.52846486112428	-0.08057792865531	8.98305865464670
C	0.44716902353736	-0.99324341334595	8.97611243050393
C	-0.43658830992033	-0.96993568202000	7.86743221924111
C	-0.23405765718985	-0.09617437732573	6.80397634762671
C	0.87906676629502	0.79507085451735	6.78609004952028
C	2.24052296721247	2.39551209186684	5.40027039138434
H	0.36813559918488	1.52808599784959	4.80787538548477
H	2.21849951890627	3.08531260462324	4.54387029562194
H	2.88309462827399	2.68476132881254	6.24540199135967
H	2.59488655657637	1.48843402533711	7.95544182965675
H	2.21307442211953	-0.06778686550705	9.84480866456948
H	-1.29358907947542	-1.66160277375401	7.83556304101183
H	-0.91619671817698	-0.10672613283280	5.93952296854046
N	0.43316991100270	-0.61464555190469	1.65961247495046
S	-0.92082016239724	-1.28281974176441	1.28785731313092
O	-0.97056204961247	-2.69930277083402	1.49869912721841
P	1.19784138241811	0.23239851313089	0.51732400786034
N	0.44565032900990	1.14507135712459	-0.55065733518519
P	-0.83827259598492	0.99841257814476	-1.48667911133513
N	-1.19999774778036	-0.31794809728240	-2.34348962022167
S	-0.77934199305660	-1.69278301617527	-1.74880557074246
C	0.06403406805334	-2.34509702425056	-3.36640564775777
F	1.15822622541365	-3.08968170216892	-3.14619682885421
O	1.96192046803619	1.30029821921054	1.48251882324659
C	2.76568164647023	2.20872970825793	0.78885829294329
C	3.93693010343458	1.77130013212039	0.19934026526058
C	4.66702981362257	2.65057316971578	-0.64968640427214
C	4.26657207674603	4.00798972210320	-0.73997065047606
C	3.20143745455197	4.45291567218597	0.06506378304510
C	2.43362137228366	3.58212525783357	0.79981198938166
C	5.76370850544091	2.21656874681111	-1.42348682384621
C	6.43290109893295	3.08911390892339	-2.23675578193519

C	6.03924545053551	4.43227615587384	-2.32249085993244
C	4.97862852568612	4.88203396123387	-1.58768172649045
C	4.46891977238351	0.45017676122511	0.58239531161307
C	3.66522132653434	-0.67078471236765	0.51673034447354
C	4.10937397099359	-1.92345155173089	1.01572869734345
C	5.41592844455412	-2.01782466328637	1.44207535380035
C	6.27507390477263	-0.90897383544401	1.51128474081213
C	5.77905156165966	0.36702260004100	1.14322066043763
C	7.59650717885441	-1.03425768457726	1.99411086030277
C	8.38839507234621	0.06687830685472	2.15664199628616
C	7.87403852561184	1.34036530213742	1.87166038369390
C	6.60288477805632	1.48955183151512	1.38515300046893
C	3.26186198427183	-3.11998877732615	1.18596981370654
C	2.41406133407457	-3.62780965150566	0.20963359572935
C	1.68052695627103	-4.78836297736587	0.43969686894673
C	1.81027339956237	-5.45341806348734	1.65511336579248
C	2.66389630612477	-4.96505873238077	2.62731527071384
C	3.37926692854639	-3.80658837598421	2.39801464685570
O	2.43075563350135	-0.56037218121955	-0.11572842456212
C	1.41828972422942	4.14364102460320	1.71682246031845
C	0.05951108345648	3.88369307239701	1.62117698290567
C	-0.84100056532502	4.50622926270273	2.48589208016330
C	-0.35076281015141	5.34368697733626	3.48756332088405
C	1.00314824209429	5.59413668413677	3.59652747894903
C	1.88367845451066	5.01485120228970	2.70219944653281
O	-0.57994129536343	2.04852400656440	-2.69662838257165
C	-0.42803326812837	3.37985808790481	-2.30918940634137
C	0.80830919313451	4.03322641899728	-2.52797797644210
C	0.96400595613281	5.32043038392299	-2.06980292829618
C	-0.05110850106951	5.99048028868797	-1.36210944755292
C	-1.33020904065515	5.38417994323464	-1.27404424411775
C	-1.51614790363024	4.08829396217617	-1.82873407328003

C	-2.36498946480840	6.07728008041023	-0.61200000358064
C	-2.14094628128553	7.31262600561068	-0.06841291456149
C	-0.87613016585905	7.91225515143886	-0.15549818368843
C	0.14698828025239	7.26480024827797	-0.79021246132791
C	1.85916706258622	3.45462388061391	-3.38997254732954
C	2.22570090475095	4.20046513223690	-4.51056694621296
C	3.18421772032483	3.72323577684416	-5.38309055912620
C	3.80349517322901	2.51407869195118	-5.13366716822742
C	3.47388189483876	1.76397465068131	-4.00474907053843
C	2.48379592717283	2.24041323957078	-3.14528115614146
C	-2.85647282102411	3.50423159122146	-2.01851539122856
C	-3.09362565930552	2.21986735279869	-1.57673393225423
C	-4.27709098843738	1.52906974201782	-1.91831255655822
C	-5.25326897439330	2.21175009829672	-2.60486755079476
C	-5.08060190509244	3.54124023290684	-3.03118049441813
C	-3.83998591836489	4.18823086252532	-2.79094102819251
C	-3.62918496636982	5.45670990381259	-3.37465345512378
C	-4.62009989570588	6.07974145934698	-4.08315127821029
C	-5.87069690993967	5.46821828805678	-4.25516172989094
C	-6.08989687016543	4.21768616270136	-3.75054811679675
O	-2.12135189442153	1.63091177426296	-0.76870732054224
C	-4.47456785000890	0.08637069803256	-1.67013596990405
C	-4.59398318218077	-0.74518267451614	-2.78241754357054
C	-4.81890978782859	-2.09888605795590	-2.62096945867834
C	-4.94345042000567	-2.63446877842329	-1.35334094331200
C	-4.84292645776454	-1.81681695300921	-0.23273312215242
C	-4.60933770007193	-0.45601878255872	-0.40347164524763
O	-1.79414646656471	-2.68717311758564	-1.59253522348202
O	0.34117324804139	-1.69888792678802	-0.83215543275768
O	-1.71600026038661	-0.66488478965952	0.25877799609221
C	-1.90733784073695	-0.69269947495770	2.85274370473001
F	-1.97702801377421	-1.61997454548203	3.82803394763705

F	-1.35073116305761	0.38823352052421	3.42997923243900
F	-3.16936308375051	-0.35169599510185	2.57594297801111
F	0.46247968237679	-1.34678696691412	-4.17543021111033
F	-0.76764598233732	-3.10302117542400	-4.10127725521145
C	-2.29195741097978	4.33503053295156	2.32929715311462
H	-0.29729132250974	3.22354725019539	0.84376596130880
H	1.37181649593499	6.24772996848932	4.37358841850669
H	2.94108041403373	5.23003980417018	2.76136638855998
H	4.02428576379895	-3.41601777201551	3.17126032358462
C	0.78019184479796	-5.33556088487402	-0.58805375973042
H	2.29359957443321	-3.11089400632638	-0.72810870469859
H	4.53328850521447	2.13569511935276	-5.83342625596907
H	1.73328256609249	5.14200945951764	-4.70595801668971
H	2.20701926459739	1.67677901298346	-2.26681787431385
C	4.18322129033003	0.50532999081711	-3.73557662310208
H	-4.48171732080494	-0.32567122551092	-3.77258948507597
H	-4.89601217656470	-2.73939892511922	-3.48780153105639
H	-4.50352957859747	0.17240193460102	0.46647618897571
H	6.05873019292489	1.17989311205781	-1.37758659140885
H	7.27165073664020	2.74341607349409	-2.82163845971015
H	6.57844097412981	5.10480531224639	-2.97283323983735
H	4.66703816196981	5.91456238743185	-1.64624351726061
H	2.97321446611155	5.50758264980121	0.09875838367160
H	6.22369950111771	2.47863410216386	1.18534108771491
H	7.96136355816503	-2.02059148125650	2.23999581444460
H	5.79466791049655	-2.97907845584874	1.75797099189835
H	-3.33456407755355	5.61129080767915	-0.52888322631002
H	-2.94315328873190	7.83223316435897	0.43327068741936
H	-0.71825653026961	8.88736618247230	0.28095238153502
H	1.12394303610471	7.71955548250566	-0.86532238540491
H	1.89651726265097	5.83136428036407	-2.25648167479130
H	-2.66899905048847	5.93489721912791	-3.26533819282139

H	-4.43917765935640	7.05065055622085	-4.51988160902164
H	-6.64598969985347	5.98127745962684	-4.80431309064520
H	-7.03545284479022	3.71746342100370	-3.90167881957175
H	-6.17208546687593	1.69943088070404	-2.85434951390993
H	9.39959501025610	-0.03263449128012	2.52126776096650
H	8.48740324830464	2.21235379547984	2.04323286080590
C	-4.98658353366873	-2.37796805221380	1.11759997619282
H	2.77420362283854	-5.48940449581603	3.56577543900225
H	3.44572855482415	4.29335793248591	-6.26273861961591
H	-1.03702704509540	5.79240635783607	4.18975552981730
H	1.25200718725663	-6.36413183779910	1.82117628404279
H	-5.12887628745445	-3.69118576312473	-1.22636586800255
C	3.55811446386428	-0.57472928396048	-3.12199761807116
C	4.24355991768266	-1.75257665100790	-2.87963274417963
C	5.58188687986343	-1.90240850926496	-3.22236033192253
C	6.20544895215441	-0.81565294194312	-3.83275120976470
C	5.52398867743778	0.35459401426843	-4.09179252657210
H	2.51645566396529	-0.50668691787271	-2.83929581103322
H	3.69988892933625	-2.55995482300262	-2.41856476195296
C	6.37589457537206	-3.17185882598611	-2.95503778655413
H	7.24607942940959	-0.87693076220179	-4.11440038382258
H	6.04792109929399	1.17618456299248	-4.55617118843300
C	1.23923429725114	-5.65425706860783	-1.85856273425630
C	0.38428819764940	-6.17457696412875	-2.81382839322380
C	-0.96018647775773	-6.39145995297336	-2.53766696232402
C	-1.40513158658140	-6.09312552656424	-1.25136659777472
C	-0.55693126595953	-5.58000409746709	-0.29202264683731
H	2.27963908857029	-5.49443078752910	-2.10355568635983
H	0.78928560346701	-6.39632659499749	-3.78832569367395
C	-1.94148231466388	-6.93581681183955	-3.56453282764605
H	-2.44016405231085	-6.25246196323348	-0.98829668796092
H	-0.93885215184210	-5.31675459833809	0.68357160881106

C	-5.82485392302368	-1.78864461843501	2.05998060807544
C	-5.94772556510211	-2.32251112005775	3.32645871112605
C	-5.24099263672758	-3.46087657158932	3.71087030050101
C	-4.40174464704306	-4.04078519342607	2.76497036719012
C	-4.28056038840485	-3.51607414488312	1.49014201111996
H	-6.39395068514597	-0.91132436949225	1.78745658239581
H	-6.60984493933668	-1.83394957703058	4.02603500308845
C	-5.40270871995782	-4.00022135910524	5.12515786084438
H	-3.81882392785413	-4.91513624076776	3.00657988946969
H	-3.59735745604536	-3.96121901554775	0.78123056239550
C	-2.85807918062211	3.12419159445496	1.94918727848360
C	-4.22689681804128	3.00126355730662	1.78637266606283
C	-5.08566554485685	4.07725508588439	1.97458888152817
C	-4.51161279798271	5.28733570965359	2.35988516149278
C	-3.15062509555777	5.41322627882994	2.54205211715585
H	-2.22839692102530	2.26223017453993	1.77778885202610
H	-4.61151303749728	2.03593852466250	1.50322219339779
C	-6.58820712616997	3.99306920197226	1.75436384244414
H	-5.13379578080145	6.15606377443984	2.51470568758247
H	-2.74167325629164	6.37248377932571	2.82243394643102
C	6.96831625439234	-3.68881338917844	-4.27530382348363
C	5.51630170063132	-4.28802375472218	-2.35445394046215
C	7.50638489366086	-2.84478815060690	-1.96742891043055
H	6.17294765526412	-3.87816036353253	-4.99386614500022
H	7.50670856022201	-4.61878062625736	-4.10428368019277
H	7.65831513402819	-2.97257419805671	-4.71207212303984
H	8.07743633268231	-3.74291723125510	-1.74181818679021
H	7.09033965709717	-2.45455966925672	-1.04100963942418
H	8.18450723536025	-2.10127407734395	-2.37748798277140
H	4.69895650439190	-4.54870683805086	-3.02397363636059
H	5.10322360631084	-3.99656940824297	-1.39168175714521
H	6.12554426965298	-5.17762716645455	-2.20410151582155

C	-4.89517976483946	-2.93890921260656	6.11374736480379
C	-4.61379009062351	-5.29211212825073	5.35868652190009
C	-6.88614933120302	-4.29716853798485	5.39280275021565
H	-4.78471028823992	-5.64273166433466	6.37475039202256
H	-3.54755872699588	-5.12491092238497	5.23245615243793
H	-4.93527263440632	-6.07303281022333	4.67330551662795
H	-7.25899329251941	-5.02149888301133	4.67130279297072
H	-7.49343929293389	-3.39978022169319	5.31744559304804
H	-7.01058315459017	-4.70881940189271	6.39234472867599
H	-5.48400697720978	-2.02772198948450	6.04863793085783
H	-3.85953185479240	-2.68765027967385	5.89196212758679
H	-4.95572656260017	-3.31499391440137	7.13302277809837
C	-3.13719772513735	-5.97629694021198	-3.66787847322209
C	-2.42001773978765	-8.32358092170588	-3.11075389553706
C	-1.32041128204660	-7.06803651076771	-4.95865978038116
H	-1.57731584508365	-9.00937750474750	-3.04782320724436
H	-3.14120770602497	-8.72282971189809	-3.82099365445463
H	-2.89124381761555	-8.27250370837823	-2.13277305472182
H	-3.79031785569577	-6.27500961004274	-4.48605869829285
H	-2.78725307346653	-4.96112452505726	-3.84729564201628
H	-3.72170420893160	-5.96889295140899	-2.75230346760870
H	-0.49632650627255	-7.77785405705247	-4.95835223625804
H	-0.96332226344831	-6.10253613906941	-5.31077360388639
H	-2.07207261462181	-7.42759126584318	-5.65891157846992
C	-6.95684239742449	4.90800725506976	0.57609684961622
C	-7.32120871554048	4.45400801009790	3.02367752235639
C	-7.05728032017812	2.57222993969120	1.42708922818247
H	-6.61407456448077	2.21300531826746	0.50085358338331
H	-8.13898256569834	2.56451787503903	1.30484628677909
H	-6.80332610773954	1.88558200538179	2.23221218782285
H	-6.69412967935133	5.94218243592263	0.78263897808693
H	-8.02622399462061	4.85794080994734	0.38179082905568

H	-6.42852614249977	4.59461155061504	-0.32255999683057
H	-7.02427026523134	3.83985491879085	3.87170379242011
H	-8.39659064330582	4.35914017441264	2.88839638925732
H	-7.10042414005518	5.49117179360564	3.25868835900305
H	-0.87438560495150	-5.66060171835428	4.77964275552039
H	-1.25439837502624	-3.88424482663929	4.61811792240998
C	-0.47316717145220	-4.66541046304437	4.51842431881537
H	-0.12885708236427	-4.64849978429272	3.46451047549522
O	0.59982570206578	-4.43502521853965	5.43902076883956
C	1.36327888026476	-3.33671743021676	5.31879533490089
H	0.50540461604106	-2.49741248677693	3.46821989794134
C	1.23576337917545	-2.38013513574547	4.28183823883955
H	2.43942609584002	-3.94879752015362	7.09859565567054
C	2.36400891455830	-3.17752403367264	6.31916676151820
C	2.07141524158162	-1.26908026647953	4.27087570346810
C	3.19768768729129	-2.07547927821272	6.29078112968202
H	1.92747472082259	-0.56184864899272	3.43920024825388
H	3.96112686808848	-1.94702369057318	7.07243745328295
C	3.06913369584781	-1.07215303468027	5.27559879614206
C	3.90300835724056	0.08404277976063	5.31341384107278
H	4.65204463925007	0.12107575194000	6.12443126323367
C	3.79373212700433	1.21132732019511	4.47878439046223
H	3.19677907294444	1.09952414121345	3.55729154369728
H	4.56573488808668	3.18974391092144	4.00781084771001
C	4.90366203117656	2.22705766621617	4.43425523317428
H	5.34193460129062	2.40300232719717	5.43634331807656
H	5.70937153058542	1.84571876245210	3.76982573848160
C	0.24027357734074	-1.97410657316338	10.09461766016163
H	-0.80630656019422	-1.94993368273817	10.46171634382973
H	0.42125385371117	-3.00959336624441	9.73374159522452
H	0.91970463820490	-1.78311436501500	10.94622203720487

TS → (1*R*,2*S*,4*R*), p-CH_3-styrene

C	2.92269975875274	2.01558081836969	7.40024153338675
C	0.76446943092581	2.05965426569375	6.10782703454169
C	-0.56178243636690	1.65801086077770	5.97219768176135
C	-1.17419575259142	0.81829324322727	6.93316181190406
C	-0.40639286823324	0.40949022025159	8.05173371178532
C	0.91740893528922	0.81084297973493	8.19837333046874
C	1.54596658816505	1.64226986026411	7.22579836327413
C	3.69706233388828	2.75184455949476	6.49932062810732
H	3.41206623875054	1.62949792598541	8.31056264104883
H	4.65480735954654	3.15476372111962	6.85995191130568
H	3.20345934862490	3.33863170498101	5.71044679715341
H	1.19834607678265	2.69826730043614	5.32485800305006
H	-1.13808497361015	1.97982113825823	5.09166517383469
H	-0.87074044943429	-0.23933152089958	8.81049019319147
H	1.49955879901883	0.47450546920940	9.06983318808997
N	0.34822101695694	-0.84102295153940	1.60098400108574
S	-0.99576815722018	-1.50057858581182	1.16476772977655
O	-1.05324669884347	-2.92718250526964	1.24597984814108
P	1.13461571515671	0.03406988370885	0.49463299671947
N	0.40453452538467	1.00637854004671	-0.53675029562916
P	-0.88896739060556	0.93113174455593	-1.46887607250308
N	-1.27143027167266	-0.33226704305807	-2.39548875477799
S	-0.87688026941164	-1.74228095052922	-1.86791076632683
C	-0.04576955157406	-2.34041759901201	-3.51258573385610
F	1.01044764171487	-3.14624615250499	-3.32040777831340
O	1.94016228698233	1.04800628774998	1.48703741875293
C	2.81036603558952	1.92074560189253	0.82815563771485
C	3.97009395714930	1.43621000083832	0.24701124222299
C	4.74948218574692	2.29118291654399	-0.58146425808890
C	4.42192004407603	3.66927987732140	-0.64965881004398
C	3.38254988159344	4.15541419119749	0.16491184368027
C	2.56144367004415	3.31274549183121	0.87509220562732

C	5.82149366659630	1.81118362106607	-1.36287889061426
C	6.53607886731762	2.65935381889213	-2.16323729418050
C	6.21290895971051	4.02244860476302	-2.22869777430136
C	5.17815054554760	4.51706145010277	-1.48558209770437
C	4.42003912932850	0.08214015714911	0.62022754757471
C	3.53239223301085	-0.97118233592245	0.52947564857139
C	3.83160202675483	-2.23803798362614	1.08665330441877
C	5.09109696029216	-2.43334454195482	1.60628212292947
C	6.05220556754850	-1.40993438383736	1.66253455731113
C	5.70224274429694	-0.10835294654644	1.21809688972071
C	7.33751467922272	-1.64533732643017	2.19850847867546
C	8.24058392311425	-0.62837967134426	2.32508814792073
C	7.87745792032560	0.67204521259450	1.94605779747085
C	6.64046480572006	0.92866296811801	1.41748735785256
C	2.85806119971601	-3.33745760108362	1.23002813743778
C	2.11645564768070	-3.86268955780999	0.18188769922075
C	1.25068592873093	-4.93409847583520	0.39007274290143
C	1.14623513505195	-5.48906315730007	1.66299304167259
C	1.88845173655136	-4.97683508554455	2.70957627119587
C	2.73835793381340	-3.90895208878525	2.49847365408861
O	2.34867033265471	-0.76144650435156	-0.17028278057979
C	1.57481921885780	3.90327916865169	1.80387240150546
C	0.21388183047246	3.64195123051754	1.74949548281005
C	-0.66341698249217	4.26221203092408	2.63941378977149
C	-0.14617563515040	5.11203518866096	3.61706939144233
C	1.20938163630570	5.37174432215390	3.67871124467664
C	2.06628676336654	4.78507127973309	2.76693944425970
O	-0.61991314331622	2.04329502143198	-2.62113081717461
C	-0.41312257084999	3.34061515013638	-2.15153922422486
C	0.84301993102309	3.96051457604805	-2.35422538794951
C	1.05778829696440	5.20976465082653	-1.82250080446904
C	0.08523337957496	5.86753310447364	-1.04634934571146

C	-1.21330278522857	5.30310085137892	-0.96565847043856
C	-1.46342971919598	4.05640427369288	-1.60232878041508
C	-2.20449695778465	5.98494693087408	-0.22943391319513
C	-1.92070277865051	7.17073562128704	0.39115134130244
C	-0.63654317843607	7.72868396269109	0.31170618161152
C	0.34535431262432	7.09072037369197	-0.39372274217018
C	1.85328160454153	3.39017378905866	-3.26916724194225
C	2.20618407854627	4.16429480708423	-4.37426845787984
C	3.12593944430762	3.69148896195758	-5.29023666413610
C	3.72083368748962	2.45954611162849	-5.09890996408903
C	3.40615023294086	1.68114088654942	-3.98500842249874
C	2.45402710015720	2.15428117545324	-3.08187136892535
C	-2.83019076383912	3.54269412549221	-1.80495490838552
C	-3.11005658401273	2.23623304242704	-1.46392515020253
C	-4.32261787821469	1.61883498446793	-1.84408237640409
C	-5.28732177198210	2.39308515285467	-2.44330743130751
C	-5.07358303112989	3.74845919467371	-2.75481836021302
C	-3.80173968464918	4.32424559025036	-2.49673364661141
C	-3.55286868063428	5.62613330843686	-2.98407709346847
C	-4.53470718210698	6.34414383663332	-3.61066345950576
C	-5.81433429412578	5.79971677885393	-3.79363723945894
C	-6.07223645535884	4.52142385231087	-3.38618769613871
O	-2.15814294049071	1.54820989694155	-0.71459356077370
C	-4.54728414359217	0.16170837770574	-1.74798086562372
C	-4.67331200899465	-0.53803559270227	-2.94722940266939
C	-4.90218988171059	-1.90065454746891	-2.94092942577479
C	-5.01978010912766	-2.57694470233605	-1.74186799993077
C	-4.91382359760142	-1.89131288235395	-0.53672157276489
C	-4.68151325620042	-0.51986109378501	-0.55036017966846
O	-1.91121187135640	-2.72315860622530	-1.76022737731142
O	0.24717805214624	-1.81127381582292	-0.95905367689798
O	-1.79137691259875	-0.78869059072926	0.19503488614906

C	-1.97175329310293	-1.06532282760097	2.77974423594534
F	-1.92270454651879	-2.04782703586450	3.70198043792125
F	-1.48546107543190	0.02448514258289	3.39582239345795
F	-3.27022796198029	-0.81887227230484	2.57025212115225
F	0.40658562116156	-1.32081706889206	-4.26423352436985
F	-0.90137298344014	-3.02163874701949	-4.29442272561125
C	-2.11537185253391	4.05895818888645	2.54124692978059
H	-0.16192353379642	2.97683699819932	0.98603933612871
H	1.59764863370872	6.03551133712689	4.43787317534937
H	3.12496317540165	4.99839640133967	2.80084408533351
H	3.30437660525394	-3.49698254279140	3.32182906693543
C	0.44983376293446	-5.47551577309881	-0.71379612361754
H	2.17730883678534	-3.41987159426900	-0.79939632930042
H	4.41932142062414	2.08705501972656	-5.83295416163503
H	1.73413237045862	5.12476327173526	-4.52260575748395
H	2.18800893661991	1.57001337664876	-2.21374229007640
C	4.09388238062357	0.40099609457349	-3.76671220269575
H	-4.56133994210679	-0.00885965348949	-3.88348452953791
H	-4.98172733996767	-2.43857341322557	-3.87490563933945
H	-4.57124736718052	0.00297887333757	0.38666595561806
H	6.06038699142341	0.75950893445608	-1.33205225913391
H	7.35481666864236	2.27816112855079	-2.75428809348010
H	6.78578187292287	4.67504314853395	-2.87053991596247
H	4.92042366745869	5.56498397686602	-1.53034217072078
H	3.21472078642689	5.22032696519507	0.22058722088471
H	6.38109062344604	1.93771626500279	1.14249711772051
H	7.58719731330922	-2.64978616200254	2.50734026124373
H	5.34999902450629	-3.40706740786355	1.99677756071875
H	-3.18828950463105	5.54833830423935	-0.15179690331449
H	-2.69032724150101	7.68314398120886	0.94834363446699
H	-0.43110101611289	8.66447229319003	0.80993550880599
H	1.33681337493875	7.51381209038686	-0.46306516003419

H	2.00398499961017	5.69799290032334	-2.00125762619153
H	-2.57081216206340	6.05522090582903	-2.86610842903887
H	-4.32404208604011	7.33934438710347	-3.97305390855880
H	-6.58157267655783	6.38681392952740	-4.27588321404097
H	-7.04105891698419	4.07251837380740	-3.55066030136763
H	-6.22791731338781	1.93686114613727	-2.71892832870305
H	9.22503251998140	-0.81410463217681	2.72736715745647
H	8.58327015265871	1.47960947020084	2.07453732808161
C	-5.01463241622650	-2.60878694591840	0.74157470999165
H	1.81429450511586	-5.41851402205715	3.69360982403255
H	3.37702205412851	4.28367292708400	-6.15833620457761
H	-0.80886305662234	5.55909312209843	4.34250392559964
H	0.48925609141615	-6.33208728071546	1.82134690554863
H	-5.19757130907553	-3.64240583036444	-1.73401284370441
C	3.46838055975304	-0.67118209513768	-3.13953997656345
C	4.14046322328933	-1.86073302164316	-2.91928076427117
C	5.46469263564040	-2.03320916197253	-3.30357569922958
C	6.08429076971495	-0.95949740285843	-3.94069402762504
C	5.41677554915011	0.22423471417924	-4.17384126473503
H	2.43788284994001	-0.58769146434603	-2.82303185621337
H	3.59686931684921	-2.65696490469431	-2.43860195654920
C	6.25061742556729	-3.30958104919586	-3.04534244712096
H	7.11263842297426	-1.04047493132493	-4.26004104208112
H	5.94024644329189	1.03629396355141	-4.65526911776067
C	0.99711746185338	-5.72713734227302	-1.96620337810696
C	0.21406084141053	-6.20044841989228	-3.00316149167775
C	-1.14517093313914	-6.43852711965590	-2.83285152375559
C	-1.67692041134873	-6.22413951325434	-1.56198155554527
C	-0.90130618245718	-5.75482122017237	-0.52365311052110
H	2.05011707199913	-5.55074944411693	-2.13313001170025
H	0.68573888159787	-6.36581852476064	-3.95864854609881
C	-2.05244461369857	-6.91430142655540	-3.95643689587038

H	-2.72434444660736	-6.41084031121266	-1.37726050455830
H	-1.34791144088543	-5.54248067634628	0.43571946220384
C	-5.86885149341542	-2.18907677098735	1.75668414091917
C	-5.91045085208430	-2.86042014746053	2.96219128709986
C	-5.09651649134740	-3.96448370432910	3.21111805718949
C	-4.25939452156478	-4.38629973133895	2.18348388569485
C	-4.22325434029675	-3.72901539076802	0.96705731316868
H	-6.50956187924493	-1.33429064661620	1.59208597928549
H	-6.58846068990246	-2.50482090579276	3.72415590945080
C	-5.14429394592095	-4.64164931867890	4.57365054800891
H	-3.60649585503777	-5.23328455013832	2.31804283760462
H	-3.53504263132529	-4.04119803628960	0.19552232260110
C	-2.66214345506454	2.84555285978750	2.13888404650262
C	-4.03235337352097	2.68264784284544	2.03330459030119
C	-4.91448825022450	3.72145165180941	2.30489472307201
C	-4.36050829107007	4.93336154954569	2.71380660729999
C	-2.99721356062340	5.09805120111101	2.83831174498412
H	-2.01630523279207	2.01034970155881	1.90632579772564
H	-4.39620815992804	1.71561505941410	1.72797507491971
C	-6.42291925967722	3.59765564958772	2.15517813794897
H	-5.00187245126123	5.77363381408158	2.93394249308014
H	-2.60847581596356	6.05912066590519	3.13932197699022
C	6.80264651989091	-3.84789756074708	-4.37435919725229
C	5.39507158264514	-4.40892326254116	-2.40904253834189
C	7.40989778948366	-2.98381390701290	-2.09072562234019
H	5.98646328113688	-4.03754920516147	-5.06910757308648
H	7.33630505368000	-4.78117161678086	-4.20659655110355
H	7.48773352826280	-3.14389154837957	-4.83785536321103
H	7.97662974662754	-3.88550114967847	-1.86862274581353
H	7.02322927043678	-2.57838136001576	-1.15773062991738
H	8.08527808616238	-2.25298886513174	-2.52724158900394
H	4.55436987522479	-4.66493947457165	-3.05091714425340

H	5.01648007931151	-4.10262133634408	-1.43666718562117
H	5.99702526113951	-5.30507121782116	-2.26861636747388
C	-4.78304576235953	-3.60673573665387	5.65012466262317
C	-4.15482243205029	-5.80557938511416	4.68771483419552
C	-6.55981115699578	-5.18394600538492	4.82103345721061
H	-4.21560305463337	-6.23772278602219	5.68476844624002
H	-3.13494315587939	-5.46315216277163	4.52566759147226
H	-4.38644231612846	-6.58750858719174	3.96809376744096
H	-6.82599212806038	-5.90267334730258	4.04859116527411
H	-7.29638006290022	-4.38545393501234	4.80889053279615
H	-6.60771589313788	-5.68067691911566	5.78789906347216
H	-5.50789592332860	-2.79810401550325	5.68222144311515
H	-3.80581313298737	-3.18158499811174	5.43192179491573
H	-4.75092104666875	-4.07824636450004	6.63010791064384
C	-3.26398927673685	-5.97280891366124	-4.05132905165079
C	-2.52347634805748	-8.34371111479174	-3.64646785513661
C	-1.34835983142692	-6.91685474476571	-5.31694296962961
H	-1.67045308343608	-9.01734960934053	-3.59308303805805
H	-3.19580555539008	-8.69447226045777	-4.42672424490465
H	-3.04817698396292	-8.38269132617114	-2.69542088789839
H	-3.84623610737969	-6.19845987273167	-4.94294562818360
H	-2.93170065302198	-4.93696846183353	-4.10029329678093
H	-3.91566227308994	-6.07489355467471	-3.18808144987266
H	-0.51418549579451	-7.61463562515071	-5.32929319595031
H	-0.98707635904856	-5.92008465909249	-5.56116692162424
H	-2.05111972216779	-7.22214387909586	-6.08989299455862
C	-6.88343236309539	4.57062244804867	1.05848477094135
C	-7.10226408125762	3.95260191097422	3.48700571274081
C	-6.86217391871246	2.18465159691874	1.76000302785972
H	-6.45762357875401	1.90361580281695	0.79011138118841
H	-7.94813054724730	2.14574091610874	1.69331990565808
H	-6.54307600936043	1.45505800353438	2.50185573805759

H	-6.65630117216613	5.60004924258433	1.32252988491257
H	-7.95749211342284	4.48486516480627	0.90742303680816
H	-6.38379950586135	4.34127212739887	0.11886358748829
H	-6.75468630046657	3.28688195147491	4.27468824080100
H	-8.18100724793970	3.84463890066225	3.39534336948294
H	-6.88844300809508	4.97515814483011	3.78435186750773
H	-0.49887063682936	-4.89980988709343	7.10072992639551
H	-0.95670558125851	-3.29940290517974	6.37465942624569
C	-0.19154832198644	-4.10299304944822	6.40054580744300
H	-0.09287525902433	-4.51392224132824	5.37432870705415
O	1.05527720660417	-3.60818129563793	6.89922294864768
C	1.67554509017812	-2.60174608800346	6.25936388996979
H	3.19114269462818	-2.59761683459010	7.80902328266012
C	2.85947522898648	-2.11432899939518	6.87920244216405
H	0.34462305840758	-2.41985720905317	4.51397253986297
C	1.22922662186273	-2.03694430884148	5.03940880340822
C	3.54830664453551	-1.05309033779131	6.31685442695238
C	1.93325794431008	-0.97941099525549	4.47441561430808
H	4.46134741741901	-0.68832598808663	6.80668823813787
H	1.56853787933351	-0.55765524744098	3.52184294145927
C	3.09253547331823	-0.43501716686536	5.10804368016459
C	3.70547140908018	0.72094192599228	4.53764797244494
H	3.24534223892738	1.08201613794737	3.59986218558605
C	4.73146659359375	1.48592229455303	5.12420800221187
H	5.30786555550817	1.00119912503577	5.92967180393560
H	6.29322369660182	1.99388390272829	3.75453110844095
C	5.48128751336760	2.50695924404556	4.31301444610235
H	4.82286653096487	2.98811765362990	3.56361575310183
H	5.95137891700072	3.28677573120706	4.94248404883325
C	-2.59089395593130	0.34553503919379	6.75713616364748
H	-3.18162456420682	1.04123024641029	6.12955012981305
H	-2.59885383962517	-0.63222443283305	6.22419082296580

H -3.10239494599775 0.19919012326510 7.72882916333402
